# Trifluoromethylated 4,5-Dihydro-1,2,4-triazin-6(1*H*)-ones via (3+3)-Annulation of Nitrile Imines with α-Amino Esters

**DOI:** 10.3390/ma16020856

**Published:** 2023-01-16

**Authors:** Anna Kowalczyk, Kamil Świątek, Małgorzata Celeda, Greta Utecht-Jarzyńska, Agata Jaskulska, Katarzyna Gach-Janczak, Marcin Jasiński

**Affiliations:** 1 Department of Organic and Applied Chemistry, Faculty of Chemistry, University of Lodz, Tamka 12, 91403 Lodz, Poland; 2 Doctoral School of Exact and Natural Sciences, University of Lodz, Banacha 12/16, 90237 Lodz, Poland; 3 Institute of Organic Chemistry, Faculty of Chemistry, Lodz University of Technology, Żeromskiego 116, 90924 Lodz, Poland; 4 Department of Biomolecular Chemistry, Medical University of Lodz, Mazowiecka 6/8, 92215 Lodz, Poland

**Keywords:** triazinones, nitrile imines, amino acids, (3+3)-annulation, fluorinated heterocycles, anticancer activity

## Abstract

The synthesis of two series of monocyclic and bicyclic trifluoromethylated 4,5-dihydro-1,2,4-triazin-6(1*H*)-one derivatives based on (3+3)-annulation of methyl esters derived from natural α-amino acids with in situ generated trifluoroacetonitrile imines has been described. The devised protocol is characterized by a wide scope, easily accessible substrates, remarkable functional group tolerance, and high chemical yield. In reactions with chiral starting materials, no racemization at the stereogenic centers was observed and the respective enantiomerically pure products were obtained. Selected functional group interconversions carried out under catalytic hydrogenation and mild PTC oxidation conditions were also demonstrated.

## 1. Introduction

The functionalization of organic molecules with fluorine atom(s) and/or with fluoroalkyl groups has been recognized as an efficient method for the tuning of their physico-chemical behavior and biological activity [1,2,3,4]. The introduction of fluorine atoms into the parent non-fluorinated compound enables control on properties such as the metabolic stability, reactivity, acidity, oleophilicity, and conformational effects, among others, which are of general significance for the search of new advanced materials [5,6,7], and compounds of potential medicinal [8,9,10] and agrochemical applications [11]. For this reason, the development of new, efficient synthetic methods leading to fluorinated products, in particular, fluoromethylated *N*-heterocycles [12,13,14], is highly desirable.

Several synthetic strategies towards F-containing heterocycles, including cycloadditions and cyclocondensation reactions, functional group interconversions, and catalytic C–H functionalizations, have been developed in recent decades and are nicely summarized elsewhere [15,16,17,18]. In this context, there is increasing interest in the chemistry of fluoroalkylated 1,3-dipoles, which are recognized as highly useful building blocks for Huisgen (3+2)-cycloaddition reactions [19,20,21,22,23,24]. For example, despite some limitations and the difficult handling of 2,2,2-trifluorodiazoethane, this highly reactive intermediate has been extensively explored, not only as 1,3-dipolar reagent, but also as a valuable source of the respective carbene employed in (2+1)-annulation reactions [22,23,24].

On the other hand, a number of more recent publications reported on nitrile imines functionalized at the *C*-termini with either CF_3_ [25,26,27,28,29,30] or CF_2_H [31,32] groups. They were successfully applied for the synthesis of various 5-membered *N*-heterocyclic systems formed via (3+2)-cycloadditions, notably, with most of the cases proceeding in a fully regioselective manner. The presented protocols also revealed fluorinated nitrile imines as readily available building blocks, which can be generated in situ under mild conditions, i.e., via the base-mediated dehydrohalogenation of bench-stable hydrazonoyl halides (or pseudohalides).

**Scheme 1:**
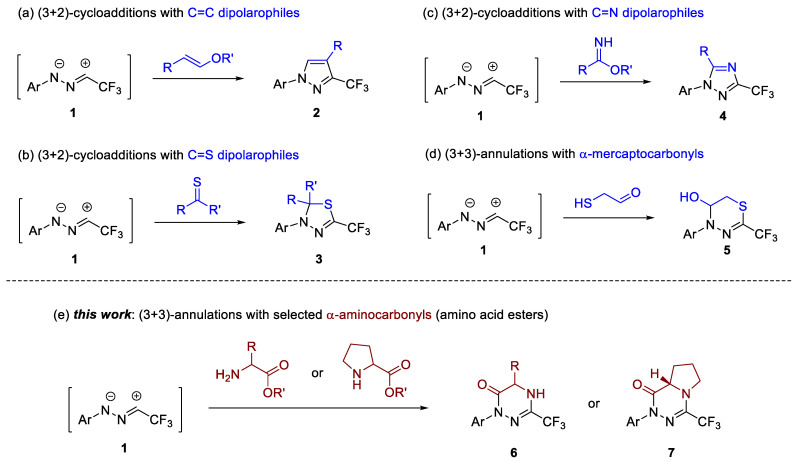
Exemplary applications of in situ generated trifluoroacetonitrile imines (**1**) in synthesis of: (**a**–**c**) 5-membered *N*-heterocycles available via (3+2)-cycloadditions with C=C and C=X dipolarophiles leading to pyrazole, 1,3,4-thiadiazole, and 1,2,4-triazole derivatives **2**–**4**, respectively; (**d**) 1,3,4-thiadiazines **5** available through (3+3)-annulation with α-mercaptoacetaldehyde; (**e**) monocyclic and bicyclic 1,3,4-triazin-6(1*H*)-ones **6** and **7** reported herein.

For example, in 2018 we have demonstrated that the trapping of in situ generated trifluoroacetonitrile imines (**1**) with electron-rich vinyl ethers leads to 3-CF_3_-pyrazole derivatives **2** (Scheme 1a) [27]. In this case, the final product is produced via the spontaneous elimination of an alcohol molecule from the initially formed (3+2)-cycloadduct. Further studies on 1,3-dipolar cycloadditions of **1** onto C=C and C≡C bonds using nitroolefins [33], cyanoalkenes [34], enones [35,36,37], alkoxyallenes [28], and benzynes [30] as dipolarophiles also evidenced the high synthetic potential of CF_3_-nitrile imines in the synthesis of pyrazoline and pyrazole derivatives. The addition of **1** with hetero-dipolarophiles are also known (Scheme 1b); a series of thiocarbonyl compounds including (cyclo)aliphatic [26,38], aromatic, and ferrocenyl thioketones [25], as well as thiochalcones [39] and thioamides [40], smoothly reacted with trifluoroacetonitrile imines **1** to afford 1,3,4-thidiazole derivatives of type **3** formed as the exclusive products. More recently, Chen and Wang disclosed an elegant method employing imidates as suitable C=N dipolarophiles en route to 1,2,4-triazoles **4** (Scheme 1c) [41].

Despite the remarkable progress in (3+2)-cycloaddition reactions in recent years, the chemistry of higher formal (3+*n*)-cycloadditions of fluorinated nitrile imines, such as **1** with bifunctional reagents, are explored to a limited extent. Some time ago, we demonstrated that nitrile imines **1** can be smoothly trapped with α-mercaptoacetaldehyde, and also with other α-mercaptocarbonyl compounds, to give 1,3,4-thiadiazolines **5** as the exclusive products (Scheme 1d) [42]. Nevertheless, to the best of our knowledge, no reports on cyclocondesations of **1** with other bifunctional compounds, such as α-aminocarbonyls, have been published thus far. Hence, we turned the attention to methyl esters derived from natural amino acids as easily available substrates for the preparation of hitherto unknown trifluoromethylated 1,3,4-triazin-6(1*H*)-one derivatives **6** and **7** (Scheme 1e).

**Figure 1:**
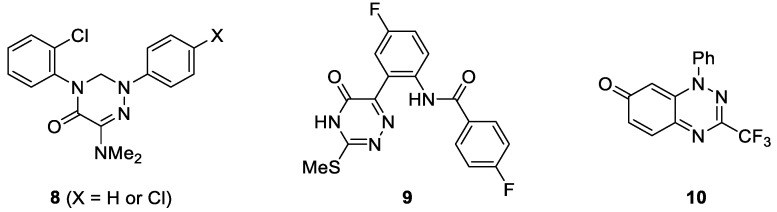
Structures of selected biologically active halogenated 1,2,4-triazinones.

In recent years, monocyclic and bicyclic 1,2,4-triazine derivatives, including their oxo analogues, attracted considerable attention, as the compounds of that type exhibit a wide range of pharmacological properties, in particular antimicrobial and anticancer activity [43,44]. For example, in 2010 Krauth et al. reported on 1,2,4-triazin-5-ones of type **8**, which showed distinct antiproliferative effects against the chronic myeloid leukemia cell line (K-562) combined with remarkably low cytotoxicity (Figure 1) [45]. Later on, Khan et al. evidenced that the introduction of fluorine atom into aryl substituent improves the biological activity of the resulting 1,2,4-triazinones (such as **9**), which were recognized as potent CDK2 and anti-HIV-1 inhibitors [46]. Furthermore, a promising thioredoxin reductase (TrxR) inhibition at submicromolar concentration by trifluoromethylated bicyclic triazinone **10** has also been discovered [47]. Thus, despite the reported progress in the synthesis and biological evaluation of fluorinated 1,2,4-triazinones, further development of synthetic protocols and the evaluation of biological properties of this group of *N*-heterocycles is of general importance.

Here we report on our results aimed at the application of nitrile imines **1** in the synthesis of a series of monocyclic and bicyclic 1,2,4-triazin-6(1*H*)-ones **6** and **7**, respectively, by using selected natural amino acid esters as suitable reaction partners. Particularly, achiral (glycine) and the selected chiral substrates bearing either primary or secondary amino group (proline) were examined. Furthermore, the subsequent transformations of the target products, particularly the oxidations of the core heterocyclic ring and interconversions of selected functional group under catalytic hydrogenation conditions, were studied. Finally, taking into account the well-documented biological activity of some fluorinated and non-fluorinated 1,2,4-triazinones and related 1,2,4-triazine-based analogues [43,44,45,46,47,48,49,50,51], the cytotoxic properties of representative final compounds were examined against MCF-7 and HL-60 cancer cell lines.

## 2. Materials and Methods

### 2.1. General Information

All commercially available chemicals (solvents, reagents) were used as received. If not stated otherwise, reactions were performed in flame dried flasks under the atmosphere of inert gas with the addition of the reactants using a syringe; subsequent manipulations were conducted in the air. NMR spectra were measured with Bruker AVIII instrument (^1^H NMR (600 MHz); ^13^C NMR (151 MHz); ^19^F NMR (565 MHz) Bruker BioSpin AG, Fällanden, Switzerland); chemical shifts are given relative to the residual undeuterated solvent peaks (for CDCl_3_: ^1^H NMR δ = 7.16, ^13^C NMR δ = 77.16; for CD_3_OD: ^1^H NMR δ = 3.31, ^13^C NMR δ = 49.00; for DMSO-*d*_6_: ^1^H NMR δ = 2.50, ^13^C NMR δ = 39.52) or to CFCl_3_ (^19^F NMR δ = 0.00) used as the external standard. Integrals in accordance with the assignments and coupling constants *J* are given in Hz. For detailed peak assignments, 2D spectra were measured (i.e., COSY, HMQC). Mass spectra (ESI) were performed with a Varian 500-MS LC Ion Trap (Varian Inc., Palo Alto, CA, USA); high resolution measurements were performed with a Waters Synapt G2-Si mass spectrometer (Waters Corporation, Milford, MA, USA). IR spectra were obtained with a Cary 630 FTIR (Agilent Technologies, Santa Clara, CA, USA) spectrometer, in neat. Elemental analyses were performed with a Vario EL III (Elementar Analysensysteme GmbH, Langenselbold, Germany) instrument. The melting points were determined in the capillaries with a Melt-Temp II (Laboratory Devices, Holliston, MA, USA) apparatus or with a polarizing optical microscope (Opta-Tech, Warsaw, Poland), and they are uncorrected. The ball-milling apparatus was a MM 400 mixer mill (Retsch GmbH, Haan, Germany). The mechanochemical reactions were performed in 5 mL stainless steel jars, at 25 Hz, with three stainless steel balls (ø 7 mm). The optical rotations were determined with a MCP 500 (Anton Paar, Graz, Austria) polarimeter at the temperatures indicated. The enantiopurity was analyzed with 1260 Infinity HPLC (Agilent Technology, Germany) using a column with chiral support (CHIRALPAK AD-H). The required known nitrile imine precursors, i.e., hydrazonoyl bromides **11**, were prepared starting with readily available trifluoroacetaldehyde arylhydrazones **14** [52], by NBS-mediated bromination of the latter, as described [25].

### 2.2. Synthetic Protocols

Synthesis of 1,2,4-triazin-6(1*H*)-ones **6** and **7**: An excess Et_3_N (8.0 mmol, 1.12 mL) was added under inert atmosphere to a suspension of amino ester hydrochloride **12** (1.0 mmol) in dry THF (3.0 mL). Then, a solution of hydrazonoyl bromide **11** (1.1 mmol) in dry THF (3.0 mL) was added, and the stirring was continued overnight (the consumption of **11** was confirmed by TLC). The resulting solution was filtered and the precipitate was washed with Et_2_O (2 × 4.0 mL). After the filtrates were combined and the solvents were removed under reduced pressure, the crude product **6** or **7** was purified by standard column chromatography (CC). In certain cases of glycine derivatives, the resulting material was additionally recrystallized from hexane-dichloromethane mixtures by the slow evaporation of the solvents.

1-(4-Nitrophenyl)-3-trifluoromethyl-4,5-dihydro-1,2,4-triazin-6(1*H*)-one (**6a**): CC (SiO_2_, CH_2_Cl_2_ gradient CH_2_Cl_2_/EtOAc 9:1), 216 mg (75%). Colorless solid, m.p. 224–225 °C (CH_2_Cl_2_/hexanes). ^1^H NMR (CDCl_3_, 600 MHz): δ 4.30 (d_br_, *J* ≈ 1.5 Hz, 2 H, CH_2_), 5.31 (s_br_, H, NH), 7.92, 8.27 (2 d_br_, *J* ≈ 9.2 Hz, 2 H each). ^13^C NMR (CDCl_3_, 151 MHz): δ 44.0 (t, CH_2_), 118.2 (q, ^1^*J*_C-F_ = 275.2 Hz, CF_3_), 123.7, 124.2 (2 d, 4 CH), 137.3 (q, ^2^*J*_C-F_ = 37.8 Hz, C(3)), 145.0, 145.5 (2 s, 2 *i*-C), 158.1 (s, C=O). ^19^F NMR (CDCl_3_, 565 MHz): δ −70.6 (s, CF_3_). IR (neat): *ν* 3295 (NH), 1685 (C=O), 1584, 1487, 1312, 1144 (CF_3_), 1059, 854 cm^−1^. ESI-MS (*m*/*z*): 311.1 (100, [*M*+Na]^+^), 289.2 (31, [*M*+H]^+^). C_10_H_7_F_3_N_4_O_3_ (288.0): calcd. C 41.68, H 2.45, N 19.44; found: C 41.50, H 2.46, N 19.61.

1-(3-Nitrophenyl)-3-trifluoromethyl-4,5-dihydro-1,2,4-triazin-6(1*H*)-one (**6b**): CC (SiO_2_, 3% MeOH in CH_2_Cl_2_), 248 mg (86%). Yellow crystals, m.p. 144–146 °C (CH_2_Cl_2_/hexanes). ^1^H NMR (CD_3_OD, 600 MHz): δ 4.19 (s, 2 H, CH_2_), 4.61 (s_br_, 1 H, NH), 7.63 (t, *J* = 8.2 Hz, 1H), 8.04 (ddd, *J* = 1.0, 2.2, 8.2 Hz, 1 H), 8.12 (ddd, *J* = 1.0, 2.2, 8.2 Hz, 1 H), 8.53 (t, *J* = 2.2 Hz, 1 H). ^13^C NMR (CD_3_OD, 151 MHz): δ 44.2 (t, CH_2_), 119.9 (q, ^1^*J*_C-F_ = 274.4 Hz, CF_3_), 119.8, 121.9, 130.5, 130.8 (4 d, 4 CH), 139.7 (q, ^2^*J*_C-F_ = 37.1 Hz, C(3)), 142.6, 149.4 (2 s, 2 *i*-C), 160.8 (s, C=O). ^19^F NMR (CD_3_OD, 565 MHz): δ −72.2 (s, CF_3_). IR (neat): *ν* 3290 (NH), 1682 and 1647 (C=O), 1536, 1472, 1349, 1271, 1197–1129 (CF_3_), 977 cm^−1^. ESI-MS (*m*/*z*): 289.2 (100, [*M*+H]^+^). C_10_H_7_F_3_N_4_O_3_ (288.0): calcd. C 41.68, H 2.45, N 19.44; found: C 41.44, H 2.36, N 19.48.

4-(3-Trifluoromethyl-4,5-dihydro-6(1*H*)-oxo-1,2,4-triazin-1-yl)benzonitrile (**6c**): CC (SiO_2_, 4% MeOH in CH_2_Cl_2_), 247 mg (92%). Colorless solid, m.p. 212–214 °C. ^1^H NMR (DMSO-d_6_, 600 MHz): δ 4.17 (s, 2 H, CH_2_), 7.79, 7.88 (2 d_br_, *J* ≈ 8.8 Hz, 2 H each), 8.67 (s_br_, 1 H, NH). ^13^C NMR (DMSO-d_6_, 151 MHz): δ 43.0 (t, CH_2_), 108.1 (s, CN), 118.4 (q, ^1^*J*_C-F_ = 275.2 Hz, CF_3_), 118.7 (s, *i*-C), 123.8, 132.7 (2 d, 4 CH), 137.4 (q, ^2^*J*_C-F_ = 36.2 Hz, C(3)), 143.8 (s, *i*-C), 159.2 (s, C=O). ^19^F NMR (DMSO-d_6_, 565 MHz): δ −69.3 (s, CF_3_). IR (neat): *ν* 3261 (NH), 2236 (CN), 1700 and 1681 (C=O), 1334, 1200–1126 (CF_3_), 1066, 839 cm^−1^. ESI-MS (*m*/*z*): 291.2 (100, [*M*+Na]^+^), 269.2 (12, [*M*+H]^+^). C_11_H_7_F_3_N_4_O (268.2): calcd. C 49.26, H 2.63, N 20.89; found: C 49.36, H 2.89, N 20.61.

1-(4-Chlorophenyl)-3-trifluoromethyl-4,5-dihydro-1,2,4-triazin-6(1*H*)-one (**6d**): CC (SiO_2_, 4% MeOH in CH_2_Cl_2_), 233 mg (84%). Yellow crystals, m.p. 159–160 °C (CH_2_Cl_2_/hexanes). ^1^H NMR (CDCl_3_, 600 MHz): δ 4.18 (d_br_, *J* ≈ 1.5 Hz, 2 H, CH_2_), 5.39 (s_br_, 1 H, NH), 7.36–7.38, 7.51–7.53 (2 m, 2 H each). ^13^C NMR (CDCl_3_, 151 MHz): δ 43.8 (t, CH_2_), 118.2 (q, ^1^*J*_C-F_ = 275.2 Hz, CF_3_), 125.7, 128.9 (2 d, 4 CH), 132.7 (s, *i*-C), 136.9 (q, ^2^*J*_C-F_ = 37.5 Hz, C(3)), 138.5 (s, *i*-C), 157.8 (s, C=O). ^19^F NMR (CDCl_3_, 565 MHz): δ −70.6 (s, CF_3_). IR (neat): *ν* 3294 (NH), 1689 and 1655 (C=O), 1491, 1349, 1316, 1194–1133 (CF_3_), 1085, 828 cm^−1^. ESI-MS (*m*/*z*): 278.2 (100, [*M*+H]^+^), 244.2 (16, [*M*–Cl+H]^+^). C_10_H_7_ClF_3_N_3_O (277.6): calcd. C 43.26, H 2.54, N 15.14; found: C 43.30, H 2.53, N 15.24.

1-(2,4-Dichlorophenyl)-3-trifluoromethyl-4,5-dihydro-1,2,4-triazin-6(1*H*)-one (**6e**): The crude reaction mixture was additionally refluxed for 2h in order to accomplish the second cyclization step (see the text), and the product was isolated after the standard work-up; CC (SiO_2_, CH_2_Cl_2_/EtOAc 95:5), 289 mg (93%). Colorless solid, m.p. 138–139 °C. ^1^H NMR (CDCl_3_, 600 MHz): δ 4.24 (s_br_, 2 H, CH_2_), 5.33 (s_br_, 1 H, NH), 7.34, 7.51 (2 m_c_, 2 H, 1 H). ^13^C NMR (CDCl_3_, 151 MHz): δ 43.7 (t, CH_2_), 118.2 (q, ^1^*J*_C-F_ = 275.2 Hz, CF_3_), 128.3, 130.3, 130.4 (3 d, 3 CH), 133.4, 135.6, 136.3 (3 s, 3 *i*-C), 136.8 (q, ^2^*J*_C-F_ = 37.6 Hz, C(3)), 157.9 (s, C=O). ^19^F NMR (CDCl_3_, 565 MHz): δ −70.5 (s, CF_3_). IR (neat): *ν* 3284 (NH), 1685 and 1665 (C=O), 1525, 1402, 1357, 1323, 1189–1129 (CF_3_), 1103, 1050 cm^−1^. ESI-MS (*m*/*z*): 312.1 (100, [*M*+H]^+^). C_10_H_6_Cl_2_F_3_N_3_O (312.1): calcd. C 38.49, H 1.94, N 13.47; found: C 38.72, H 2.19, N 13.54.

Amidrazone **13e**: The spectroscopically pure sample of intermediate **13e** (75 mg, 22%; as ca. 87:13 mixture of *E*/*Z*-isomers) was obtained in reaction of **11e** with **12a** and was isolated from the mother liquor by preparative thin layer chromatography (PTLC; SiO_2_, petroleum ether/CH_2_Cl_2_ 3:2) followed by recrystallisation from a hexanes/CH_2_Cl_2_ mixture. Colorless solid, m.p. 79–81 °C. ^1^H NMR (CDCl_3_, 600 MHz); major isomer: δ 3.82 (s, 3 H, Me), 4.05 (d, *J* = 5.7 Hz, 2 H), 4.57 (t_br_, *J* ≈ 5.7 Hz, 1 H, NH), 7.20 (dd, *J* = 2.3, 8.8 Hz, 1 H), 7.21 (s_br_, 1 H, NH), 7.29 (d, *J* = 2.3 Hz, 1 H), 7.39 (d, *J* = 8.8 Hz, 1 H); diagnostic signals for minor isomer: δ 3.80 (s, 3 H, Me), 4.00 (d, *J* = 5.2 Hz, 2 H), 4.95 (t_br_, *J* ≈ 5.2 Hz, 1 H, NH), absorptions in the aromatic region could not be seen due to overlap of the signals. ^13^C NMR (CDCl_3_, 151 MHz); major isomer: δ 44.8 (t, CH_2_), 53.0 (q, Me), 116.4 (d, CH), 119.20 (q, ^1^*J*_C-F_ = 274.5 Hz, CF_3_), 119.23, 125.7 (2 s, 2 *i*-C), 128.2, 128.7 (2 d, 2 CH), 136.7 (q, ^2^*J*_C-F_ = 34.9 Hz), 140.4 (s, *i*-C), 170.8 (s, C=O); diagnostic signals for minor isomer: δ 43.8 (t, CH_2_), 52.6 (q, Me), 114.1 (d, CH), 117.8, 123.8 (2 s, 2 *i*-C), 128.0, 128.6 (2 d, 2 CH), 140.7 (s, *i*-C), 170.4 (s, C=O); absorptions of the CF_3_ group and of the neighboring C atom could not be found due to overlap and low intensity, respectively.

1-Phenyl-3-trifluoromethyl-4,5-dihydro-1,2,4-triazin-6(1*H*)-one (**6f**): CC (SiO_2_, 4% MeOH in CH_2_Cl_2_), 192 mg (79%). Colorless crystals, m.p. 146–148 °C (CH_2_Cl_2_/hexanes). ^1^H NMR (CDCl_3_, 600 MHz): δ 4.13 (d_br_, *J* ≈ 1.6 Hz, 2 H, CH_2_), 5.39 (s_br_, 1 H, NH), 7.28–7.31, 7.40–7.43, 7.52–7.54 (3 m, 1 H, 2 H, 2 H). ^13^C NMR (CDCl_3_, 151 MHz): δ 43.8 (t, CH_2_), 118.3 (q, ^1^*J*_C-F_ = 275.1 Hz, CF_3_), 124.7, 127.5, 128.9 (3 d, 5 CH), 136.7 (q, ^2^*J*_C-F_ = 37.6 Hz, C(3)), 140.0 (s, *i*-C), 157.9 (s, C=O). ^19^F NMR (CDCl_3_, 565 MHz): δ −70.6 (s, CF_3_). IR (neat): *ν* 3288 (NH), 1689 and 1655 (C=O), 1495, 1394, 1353, 1320, 1193–1137 (CF_3_), 1092, 1057 cm^−1^. ESI-MS (*m*/*z*): 244.2 (100, [*M*+H]^+^). C_10_H_8_F_3_N_3_O (243.2): calcd. C 49.39, H 3.32, N 17.28; found: C 49.23, H 3.47, N 17.30.

1-(4-Tolyl)-3-trifluoromethyl-4,5-dihydro-1,2,4-triazin-6(1*H*)-one (**6g**): CC (SiO_2_, 5% MeOH in CH_2_Cl_2_), 195 mg (76%). Colorless crystals, m.p. 153–154 °C (CH_2_Cl_2_/hexanes). ^1^H NMR (CDCl_3_, 600 MHz): δ 2.35 (s, 3 H, Me), 4.14 (d_br_, *J* ≈ 1.5 Hz, 2 H, CH_2_), 5.37 (s_br_, 1 H, NH), 7.21, 7.39 (2 d_br_, *J* ≈ 8.2 Hz, 2 H each). ^13^C NMR (CDCl_3_, 151 MHz): δ 21.2 (q, Me), 43.8 (t, CH_2_), 118.3 (q, ^1^*J*_C-F_ = 275.0 Hz, CF_3_), 124.7, 129.5 (2 d, 4 CH), 136.6 (q, ^2^*J*_C-F_ = 37.4 Hz, C(3)), 137.45, 137.53 (2 s, 2 *i*-C), 157.8 (s, C=O). ^19^F NMR (CDCl_3_, 565 MHz): δ −70.6 (s, CF_3_). IR (neat): *ν* 3243 (NH), 1681 and 1648 (C=O), 1513, 1402, 1357, 1316, 1189–1133 (CF_3_), 1085, 820 cm^−1^. ESI-MS (*m*/*z*): 258.2 (100, [*M*+H]^+^). C_11_H_10_F_3_N_3_O (257.2): calcd. C 51.37, H 3.92, N 16.34; found: C 51.33, H 3.93, N 16.80.

1-(4-Benzyloxyphenyl)-3-trifluoromethyl-4,5-dihydro-1,2,4-triazin-6(1*H*)-one (**6h**): CC (SiO_2_, 2% MeOH in CH_2_Cl_2_), 286 mg (82%). Light orange crystals, m.p. 130–132 °C (CH_2_Cl_2_/hexanes). ^1^H NMR (CDCl_3_, 600 MHz): δ 4.15 (d_br_, *J* ≈ 1.4 Hz, 2 H, NCH_2_), 5.07 (s, 2 H, OCH_2_), 5.33 (s_br_, 1 H, NH), 6.98–7.01, 7.32–7.34, 7.37–7.43 (3 m, 2 H, 1 H, 6 H). ^13^C NMR (CDCl_3_, 151 MHz): δ 43.8 (t, NCH_2_), 70.3 (t, OCH_2_), 115.1 (d, 2 CH), 118.3 (q, ^1^*J*_C-F_ = 275.1 Hz, CF_3_), 126.3, 127.6, 128.2, 128.7 (4 d, 7 CH), 133.3 (s, *i*-C), 136.5 (q, ^2^*J*_C-F_ = 37.3 Hz, C(3)), 136.8, 157.8, 157.9 (3 s, 2 *i*-C, C=O). ^19^F NMR (CDCl_3_, 565 MHz): δ −70.5 (s, CF_3_). IR (neat): *ν* 3276 (NH), 1692–1651 (C=O), 1506, 1349, 1327, 1185–1141 (CF_3_), 1081, 1051 cm^−1^. ESI-MS (*m*/*z*): 372.3 (100, [*M*+Na]^+^), 350.4 (52, [*M*+H]^+^). C_17_H_14_F_3_N_3_O_2_ (349.3): calcd. C 58.45, H 4.04, N 12.03; found: C 58.23, H 4.12, N 12.20.

(*S*)-5-Methyl-1-(4-nitrophenyl)-3-trifluoromethyl-4,5-dihydro-1,2,4-triazin-6(1*H*)-one (**6i**): CC (SiO_2_, CH_2_Cl_2_), 233 mg (77%). Light orange solid, m.p. 132–133 °C. [α]_D_^20^ = −31.7 (*c* 0.26, CHCl_3_). ^1^H NMR (CD_3_OD, 600 MHz): δ 1.51 (d, *J* = 6.8 Hz, 3 H, CH_3_), 4.33 (q, *J* = 6.8 Hz, 1 H, 5-H), 7.91–7.93, 8.26–8.28 (2 m, 2 H each). ^13^C NMR (CD_3_OD, 151 MHz): δ 19.2 (q, Me), 50.8 (d, C(5)), 120.0 (q, ^1^*J*_C-F_ = 274.5 Hz, CF_3_), 124.8, 125.0 (2 d, 4 CH), 139.7 (q, ^2^*J*_C-F_ = 37.2 Hz, C(3)), 146.5, 147.0 (2 s, 2 *i*-C), 164.2 (s, C=O). ^19^F NMR (CD_3_OD, 565 MHz): δ −68.5 (s, CF_3_). IR (neat): *ν* 3247 (NH), 1685 (C=O), 1588, 1517, 1331, 1197, 1140–1082 (CF_3_), 855 cm^−1^. ESI-MS (*m*/*z*): 325.2 (100, [*M*+Na]^+^). C_11_H_9_F_3_N_4_O_3_ (302.2): calcd. C 43.72, H 3.00, N 18.54; found: C 43.60, H 3.17, N 18.66.

(*S*)-5-(1-Methylethyl)-1-(4-nitrophenyl)-3-trifluoromethyl-4,5-dihydro-1,2,4-triazin-6(1*H*)-one (**6j**): CC (SiO_2_, petroleum ether/EtOAc 4:1), 271 mg (82%). Pale yellow solid, m.p. 143–144 °C. [α]_D_^20^ = −213.4 (*c* 0.27, CHCl_3_). ^1^H NMR (CDCl_3_, 600 MHz): δ 1.02 (d, *J* = 6.8 Hz, 3 H, CH_3_), 1.07 (d, *J* = 7.0 Hz, 3 H, CH_3_), 2.36–2.44 (m, 1 H), 4.14 (dd_br_, *J* ≈ 2.4, 3.9 Hz, 1 H, 5-H), 5.44 (s_br_, 1 H, NH), 7.88–7.90, 8.24–8.27 (2 m, 2 H each). ^13^C NMR (CDCl_3_, 151 MHz): δ 16.7, 18.3 (2 q, 2 Me), 33.2 (d, *C*HMe_2_), 59.5 (d, C(5)), 117.4 (q, ^1^*J*_C-F_ = 275.4 Hz, CF_3_), 123.9, 124.2 (2 d, 4 CH), 137.4 (q, ^2^*J*_C-F_ = 37.5 Hz, C(3)), 145.3, 145.4 (2 s, 2 *i*-C), 160.9 (s, C=O). ^19^F NMR (CDCl_3_, 565 MHz): δ −70.8 (s, CF_3_). IR (neat): *ν* 3284 (NH), 1689 and 1659 (C=O), 1524, 1495, 1334, 1193, 1148–1111 (CF_3_), 1081, 850 cm^−1^. ESI-MS (*m*/*z*): 353.2 (100, [*M*+Na]^+^), 331.3 (20, [*M*+H]^+^). C_13_H_13_F_3_N_4_O_3_ (330.3): calcd. C 47.28, H 3.97, N 16.96; found: C 47.38, H 4.12, N 16.83.

(*S*)-5-(2-Methylpropyl)-1-(4-nitrophenyl)-3-trifluoromethyl-4,5-dihydro-1,2,4-triazin-6(1*H*)-one (**6k**): CC (SiO_2_, petroleum ether/CH_2_Cl_2_ 1:3), 186 mg (54%). Light orange solid, m.p. 107–108 °C (hexanes). [α]_D_^20^ = −190.6 (*c* 0.23, CHCl_3_). ^1^H NMR (CDCl_3_, 600 MHz): δ 0.98, 1.01 (2 d, *J* = 6.1 Hz, 3 H each, 2 CH_3_), 1.69–1.83 (m, 3 H), 4.27 (ddd, *J* = 2.2, 4.6, 8.4 Hz, 1 H, 5-H), 5.59 (s_br_, 1 H, NH), 7.88–7.90, 8.23–8.25 (2 m, 2 H each). ^13^C NMR (CDCl_3_, 151 MHz): δ 21.8, 22.9 (2 q, 2 Me), 24.2 (d, *C*HMe_2_), 41.9 (t, CH_2_), 52.7 (d, C(5)), 118.2 (q, ^1^*J*_C-F_ = 275.3 Hz, CF_3_), 123.6, 124.2 (2 d, 4 CH), 137.4 (q, ^2^*J*_C-F_ = 37.5 Hz, C(3)), 145.2, 145.4 (2 s, 2 *i*-C), 161.7 (s, C=O). ^19^F NMR (CDCl_3_, 565 MHz): δ −70.8 (s, CF_3_). IR (neat): *ν* 3295 (NH), 1685 and 1659 (C=O), 1521, 1331, 1193–1123 (CF_3_), 1081, 849 cm^−1^. ESI-MS (*m*/*z*): 345.3 (100, [*M*+H]^+^). C_14_H_15_F_3_N_4_O_3_ (344.3): calcd. C 48.84, H 4.39, N 16.27; found: C 48.83, H 4.40, N 16.53.

(*S*)-1-(4-Nitrophenyl)-5-phenyl-3-trifluoromethyl-4,5-dihydro-1,2,4-triazin-6(1*H*)-one (**6l**): CC (SiO_2_, CH_2_Cl_2_/hexanes 3:1), 211 mg (58%). Pale yellow solid, m.p. 116–117 °C. [α]_D_^20^ = +20.7 (*c* 0.28, CHCl_3_). ^1^H NMR (CDCl_3_, 600 MHz): δ 5.32 (d_br_, *J* ≈ 2.0 Hz, 1 H, 5-H), 5.83 (s_br_, 1 H, NH), 7.41–7.46, 7.86–7.89, 8.21–8.24 (3 m, 5 H, 2 H, 2 H each). ^13^C NMR (CDCl_3_, 151 MHz): δ 58.2 (d, C(5)), 118.3 (q, ^1^*J*_C-F_ = 275.5 Hz, CF_3_), 123.8, 124.2, 126.8, 129.6, 129.8 (5 d, 9 CH), 136.8 (q, ^2^*J*_C-F_ = 37.7 Hz, C(3)), 137.2, 145.2, 145.5 (3 s, 3 *i*-C), 159.6 (s, C=O). ^19^F NMR (CDCl_3_, 565 MHz): δ −70.4 (s, CF_3_). IR (neat): *ν* 3291 (NH), 1688 (C=O), 1592, 1517, 1320, 1193–1141 (CF_3_), 1081, 854 cm^−1^. ESI-MS (*m*/*z*): 387.2 (100, [*M*+Na]^+^), 365.4 (20, [*M*+H]^+^). C_16_H_11_F_3_N_4_O_3_ (364.3): calcd. C 52.75, H 3.04, N 15.38; found: C 52.69, H 3.13, N 15.21.

(*S*)-5-(Hydroxymethyl)-1-(4-nitrophenyl)-3-trifluoromethyl-4,5-dihydro-1,2,4-triazin-6(1*H*)-one (**6m**): CC (SiO_2_, CH_2_Cl_2_/EtOAc 6:1), 239 mg (75%). Yellow solid, m.p. 127–128 °C. [α]_D_^20^ = −96.5 (*c* 0.28, MeCN). ^1^H NMR (CD_3_OD, 600 MHz): δ 3.72 (dd, *J* = 2.8, 11.6 Hz, 1 H, CH_2_), 4.08 (dd, *J* = 3.1, 11.6 Hz, 1 H, CH_2_), 4.33 (pseudo-t, *J* ≈ 2.9 Hz, 5-H), 7.93–7.95, 8.25–8.27 (2 m, 2 H each). ^13^C NMR (CD_3_OD, 151 MHz): δ 57.8 (d, C(5)), 64.7 (t, CH_2_), 119.9 (q, ^1^*J*_C-F_ = 274.5 Hz, CF_3_), 124.7, 125.2 (2 d, 4 CH), 139.7 (q, ^2^*J*_C-F_ = 36.9 Hz, C(3)), 146.5, 147.1 (2 s, 2 *i*-C), 162.5 (s, C=O). ^19^F NMR (CD_3_OD, 565 MHz): δ −72.1 (s, CF_3_). IR (neat): *ν* 3411 (OH), 3198 (NH), 1692 and 1659 (C=O), 1513, 1327, 1193–1088 (CF_3_), 1036 cm^−1^. ESI-MS (*m*/*z*): 341.1 (100, [*M*+Na]^+^), 319.2 (13, [*M*+H]^+^). C_11_H_9_F_3_N_4_O_4_ (318.1): calcd. C 41.52, H 2.85, N 17.61; found: C 41.60, H 3.04, N 17.33.

(*S*)-5-[2-(Methylthio)ethyl]-1-(4-nitrophenyl)-3-trifluoromethyl-4,5-dihydro-1,2,4-triazin-6(1*H*)-one (**6n**): CC (SiO_2_, petroleum ether/EtOAc 3:1), 329 mg (91%). Thick yellow oil. [α]_D_^20^ = −93.0 (*c* 0.25, CHCl_3_). ^1^H NMR (CDCl_3_, 600 MHz): δ 2.14 (s, 3 H, Me), 2.15–2.20, 2.32–2.37, 2.68–2.79 (3 m, 1 H, 1 H, 2 H), 4.45 (ddd, *J* = 1.9, 4.1, 7.9 Hz, 1 H, 5-H), 6.07 (s_br_, 1 H, NH), 7.88–7.90, 8.24–8.27 (2 m, 2 H each). ^13^C NMR (CDCl_3_, 151 MHz): δ 15.3 (q, Me), 30.4, 31.2 (2 t, 2 CH_2_), 54.0 (d, C(5)), 118.2 (q, ^1^*J*_C-F_ = 275.5 Hz, CF_3_), 123.8, 124.2 (2 d, 4 CH), 137.3 (q, ^2^*J*_C-F_ = 37.5 Hz, C(3)), 145.2, 145.4 (2 s, 2 *i*-C), 161.0 (s, C=O). ^19^F NMR (CDCl_3_, 565 MHz): δ −70.8 (s, CF_3_). IR (neat): *ν* 3302 (NH), 1689 and 1657 (C=O), 1592, 1517, 1327, 1193–1108 (CF_3_), 1081, 854 cm^−1^. (-)-ESI-MS (*m*/*z*): 360.9 (100, [*M*–H]^−^), 350.1 (13). C_13_H_13_F_3_N_4_O_3_S (362.1): calcd. C 43.09, H 3.62, N 15.46, S 8.85; found: C 42.99, H 3.73, N 15.67, S 8.78.

Methyl (*S*)-2-[1-(4-nitrophenyl)-3-trifluoromethyl-6(1*H*)-oxo-4,5-dihydro-1,2,4-triazin-5-yl]acetate (**6o**): CC (SiO_2_, petroleum ether/CH_2_Cl_2_ 2:1), 335 mg (93%). Yellow solid, m.p. 114–115 °C (hexanes). [α]_D_^20^ = −82.0 (*c* 0.24, CHCl_3_). ^1^H NMR (CDCl_3_, 600 MHz): δ 2.90 (dd, *J* = 10.3, 17.6 Hz, 1 H, CH_2_), 3.24 (dd, *J* = 2.8, 17.6 Hz, 1 H, CH_2_), 3.79 (s, 3 H, Me), 4.66 (ddd, *J* = 1.8, 2.8, 10.3 Hz, 1 H, 5-H), 6.19 (s_br_, 1 H, NH), 7.90, 8.26 (2 d_br_, *J* ≈ 9.2 Hz, 2 H each). ^13^C NMR (CDCl_3_, 151 MHz): δ 37.1 (t, CH_2_), 50.8 (d, C(5)), 52.8 (q, Me), 118.1 (q, ^1^*J*_C-F_ = 275.4 Hz, CF_3_), 123.8, 124.2 (2 d, 4 CH), 137.3 (q, ^2^*J*_C-F_ = 37.8 Hz, C(3)), 145.0, 145.6 (2 s, 2 *i*-C), 159.8, 171.6 (2 s, 2 C=O). ^19^F NMR (CDCl_3_, 565 MHz): δ −70.8 (s, CF_3_). IR (neat): *ν* 3396 (NH), 1715 (C=O), 1681 (C=O), 1588, 1521, 1491, 1390, 1323, 1193–1139 (CF_3_), 1107, 857 cm^−1^. ESI-MS (*m*/*z*): 383.2 (100, [*M*+Na]^+^). C_13_H_11_F_3_N_4_O_5_ (360.2): calcd. C 43.34, H 3.08, N 15.55; found: C 43.07, H 2.95, N 15.61.

(*S*)-5-[(Indol-3-yl)methyl]-1-(4-nitrophenyl)-3-trifluoromethyl-4,5-dihydro-1,2,4-triazin-6(1*H*)-one (**6p**): CC (SiO_2_, EtOAc), 346 mg (83%). Pale yellow solid, m.p. 140–141 °C. [α]_D_^20^ = −143.6 (*c* 0.24, CHCl_3_). ^1^H NMR (CDCl_3_, 600 MHz): δ 3.24 (dd, *J* = 9.4, 14.6 Hz, 1 H, CH_2_), 3.53 (dd, *J* = 3.4, 14.6 Hz, 1 H, CH_2_), 4.54 (ddd, *J* = 1.7, 3.4, 9.4 Hz, 1 H, 5-H), 5.32 (s_br_, 1 H, NH), 7.13–7.17, 7.25–7.28 (2 m, 2 H, 1 H), 7.43 (d_br_, *J* ≈ 8.2 Hz, 1 H), 7.63 (d_br_, *J* ≈ 7.9 Hz, 1 H), 7.77–7.80, 8.22–8.24 (2 m, 2 H each) °8.24 (s_br_, 1 H, NH). ^13^C NMR (CDCl_3_, 151 MHz): δ 30.1 (t, CH_2_), 54.4 (d, C(5)), 108.5 (s, *i*-C), 111.7 (d, CH), 118.2 (q, ^1^*J*_C-F_ = 275.3 Hz, CF_3_), 118.6, 120.4, 123.1, 123.8, 123.9, 124.1 (6 d, 8 CH), 126.7, 136.6 (2 s, 2 *i*-C), 137.0 (q, ^2^*J*_C-F_ = 37.6 Hz, C(3)), 145.2, 145.4 (2 s, 2 *i*-C), 161.39 (s, C=O). ^19^F NMR (CDCl_3_, 565 MHz): δ −70.6 (s, CF_3_). IR (neat): *ν* 3351 (NH), 1689 and 1662 (C=O), 1592, 1517, 1327, 1200–1144 (CF_3_), 1085, 854 cm^−1^. (-)-ESI-MS (*m*/*z*): 416.0 (88, [*M*–H]^−^). C_19_H_14_F_3_N_5_O_3_ (417.3): calcd. C 54.68, H 3.38, N 16.78; found: C 54.43, H 3.49, N 16.74.

(*S*)-5-Phenyl-1-(4-tolyl)-3-trifluoromethyl-4,5-dihydro-1,2,4-triazin-6(1*H*)-one ((*S*)-**6q**): CC (SiO_2_, CH_2_Cl_2_), 250 mg (75%). Colorless crystals, m.p. 153–154 °C. [α]_D_^20^ = +171.6 (*c* 0.28, CHCl_3_). ^1^H NMR (CD_3_OD, 600 MHz): δ 2.35 (s, 3 H, CH_3_), 5.28 (s_br_, 1 H, NH), 7.22, 7.32 (d_br_, *J* ≈ 8.3 Hz, 2 H each), 7.37–7.40, 7.42–7.45 (2 m, 1 H, 4 H). ^13^C NMR (CD_3_OD, 151 MHz): δ 21.1 (q, Me), 58.9 (d, C(5)), 120.1 (q, ^1^*J*_C-F_ = 274.4 Hz, CF_3_), 126.1, 127.9, 129.9, 130.1, 130.2 (5 d, 9 CH), 138.5 (s, *i*-C), 138.6 (q, ^2^*J*_C-F_ = 37.0 Hz, C(3)), 139.4, 140.7 (2 s, 2 *i*-C), 161.6 (s, C=O). ^19^F NMR (CD_3_OD, 565 MHz): δ −72.1 (s, CF_3_). IR (neat): *ν* 3336 (NH), 1677 and 1655 (C=O), 1510, 1338, 1189–1144 (CF_3_), 1084, 820 cm^−1^. ESI-MS (*m*/*z*): 372.1 (57, [*M*+K]^+^), 356.1 (47, [*M*+Na]^+^), 334.2 (100, [*M*+H]^+^). C_17_H_14_F_3_N_3_O (333.3): calcd. C 61.26, H 4.23, N 12.61; found: C 61.08, H 4.37, N 12.86. A sample of *rac*-**6q** (276 mg, 83%) was prepared in an analogous manner starting with hydrazonoyl bromide **11g** and racemic methyl phenylglycinate (*rac*-**12e**). The obtained ^1^H and ^13^C NMR data perfectly matched those obtained for (*S*)-**6q**.

(*S*)-2-(4-Nitrophenyl)-4-trifluoromethyl-6,7,8,8a-tetrahydropyrrolo [1,2-*d*][1,2,4]-triazin-1(2*H*)-one (**7a**): CC (SiO_2_, CH_2_Cl_2_), 269 mg (82%). Yellow solid, m.p. 90–92 °C (CH_2_Cl_2_/hexanes). [α]_D_^20^ = +79.2 (*c* 0.17, CHCl_3_). ^1^H NMR (CDCl_3_, 600 MHz): δ 2.08–2.14 (m, 2 H, 7-H_2_), 2.28–2.34 (m, 1 H, 8-H), 2.46–2.51 (m, 1 H, 8-H), 3.75 (t_br_, *J* ≈ 7.4 Hz, 2 H, 6-H_2_), 4.17 (dd, *J* = 6.9, 8.9 Hz, 1 H, 8a-H), 7.92–7.94, 8.24–8.26 (2 m, 2 H each). ^13^C NMR (CDCl_3_, 151 MHz): δ 24.1 (t, C(7)), 28.3 (t, C(8)), 48.8 (q, ^4^*J*_C-F_ = 2.3 Hz, C(6)), 58.4 (d, C(8a)), 118.5 (q, ^1^*J*_C-F_ = 276.0 Hz, CF_3_), 123.5, 124.2 (2 d, 4 CH), 138.1 (q, ^2^*J*_C-F_ = 36.5 Hz, C(4)), 145.2* (s, 2 *i*-C), 161.5 (s, C=O); *higher intensity. ^19^F NMR (CDCl_3_, 565 MHz): δ −68.6 (s, CF_3_). IR (neat): *ν* 1698 (C=O), 1519, 1452, 1344, 1191, 1135 (CF_3_) cm^−1^. ESI-MS (*m*/*z*): 329.3 (100, [*M*+H]^+^). C_13_H_11_F_3_N_4_O_3_ (328.2): calcd. C 47.57, H 3.38, N 17.07; found: C 47.78, H 3.48, N 17.19.

(*S*)-2-(3-Nitrophenyl)-4-trifluoromethyl-6,7,8,8a-tetrahydropyrrolo [1,2-*d*][1,2,4]-triazin-1(2*H*)-one (**7b**): CC (SiO_2_, CH_2_Cl_2_), 213 mg (65%). Thick yellow oil. [α]_D_^20^ = +27.4 (*c* 0.15, CHCl_3_). ^1^H NMR (CDCl_3_, 600 MHz): δ 2.06–2.14 (m, 2 H, 7-H_2_), 2.27–2.33 (m, 1 H, 8-H), 2.45–2.51 (m, 1 H, 8-H), 3.71–3.77 (m, 2 H, 6-H_2_), 4.17 (dd, *J* = 6.9, 8.9 Hz, 1 H, 8a-H), 7.55 (t, *J* = 8.2 Hz, 1H), 8.01 (ddd, *J* = 1.0, 2.2, 8.2 Hz, 1 H), 8.10 (ddd, *J* = 1.0, 2.2, 8.2 Hz, 1 H), 8.56 (t, *J* = 2.2 Hz, 1 H). ^13^C NMR (CDCl_3_, 151 MHz): δ 24.1 (t, C(7)), 28.3 (t, C(8)), 48.8 (q, ^4^*J*_C-F_ = 2.4 Hz, C(6)), 58.3 (d, C(8a)), 118.5 (q, ^1^*J*_C-F_ = 276.0 Hz, CF_3_), 119.0, 121.2, 129.4, 129.6 (4 d, 4 CH), 138.0 (q, ^2^*J*_C-F_ = 36.5 Hz, C(4)), 141.0, 148.4 (2 s, 2 *i*-C), 161.3 (s, C=O). ^19^F NMR (CDCl_3_, 565 MHz): δ −68.5 (s, CF_3_). IR (neat): *ν* 1696 and 1648 (C=O), 1528, 1454, 1349, 1193–1118 (CF_3_) cm^−1^. ESI-MS (*m*/*z*): 329.3 (64, [*M*+H]^+^), 327.3 (100, [*M*–H]^+^), 299.2 (36). C_13_H_11_F_3_N_4_O_3_ (328.2): calcd. C 47.57, H 3.38, N 17.07; found: C 47.75, H 3.43, N 16.84.

(*S*)-4-[4-Trifluoromethyl-1(2*H*)-oxo-6,7,8,8a-tetrahydropyrrolo [1,2-*d*][1,2,4]triazin-2-yl]benzonitrile (**7c**): CC (SiO_2_, CH_2_Cl_2_), 286 mg (93%). Light orange solid, m.p. 103–105 °C. [α]_D_^20^ = +68.3 (*c* 0.16, CHCl_3_). ^1^H NMR (CDCl_3_, 600 MHz): δ 2.07–2.13 (m, 2 H, 7-H_2_), 2.26–2.33 (m, 1 H, 8-H), 2.45–2.50 (m, 1 H, 8-H), 3.71–3.76 (m, 2 H, 6-H_2_), 4.16 (dd, *J* = 6.9, 8.9 Hz, 1 H, 8a-H), 7.66–7.69, 7.84–7.86 (2 m, 2 H each). ^13^C NMR (CDCl_3_, 151 MHz): δ 24.1 (t, C(7)), 28.3 (t, C(8)), 48.8 (q, ^4^*J*_C-F_ = 2.3 Hz, C(6)), 58.4 (d, C(8a)), 109.7 (s, CN), 118.5 (q, ^1^*J*_C-F_ = 275.8 Hz, CF_3_), 118.8 (s, *i*-C), 123.8, 132.7 (2 d, 4 CH), 138.0 (q, ^2^*J*_C-F_ = 36.3 Hz, C(4)), 143.7 (s, *i*-C), 161.3 (s, C=O). ^19^F NMR (CDCl_3_, 565 MHz): δ −68.5 (s, CF_3_). IR (neat): *ν* 2223 (CN), 1690 and 1655 (C=O), 1603, 1452, 1315, 1126 (CF_3_) cm^−1^. ESI-MS (*m*/*z*): 331.3 (29, [*M*+Na]^+^), 309.3 (100, [*M*+H]^+^). C_14_H_11_F_3_N_4_O (308.3): calcd. C 54.55, H 3.60, N 18.18; found: C 54.61, H 3.73, N 18.13.

(*S*)-2-(4-Chlorophenyl)-4-trifluoromethyl-6,7,8,8a-tetrahydropyrrolo [1,2-*d*][1,2,4]triazin-1(2*H*)-one (**7d**): Reaction time: 2d; CC (SiO_2_, petroleum ether/EtOAc 4:1), 219 mg (69%). Pale yellow solid, m.p. 86–87 °C. [α]_D_^20^ = +35.1 (*c* 0.19, CHCl_3_). ^1^H NMR (CDCl_3_, 600 MHz): δ 2.03–2.12 (m, 2 H, 7-H_2_), 2.24–2.31 (m, 1 H, 8-H), 2.43–2.48 (m, 1 H, 8-H), 3.68–3.75 (m, 2 H, 6-H_2_), 4.13 (dd, *J* = 6.9, 8.9 Hz, 1 H, 8a-H), 7.34–7.36, 7.53–7.55 (2 m, 2 H each). ^13^C NMR (CDCl_3_, 151 MHz): δ 24.1 (t, C(7)), 28.4 (t, C(8)), 48.7 (q, ^4^*J*_C-F_ = 2.3 Hz, C(6)), 58.3 (d, C(8a)), 118.6 (q, ^1^*J*_C-F_ = 275.7 Hz, CF_3_), 125.5, 128.8 (2 d, 4 CH), 132.3 (s, *i*-C), 137.6 (q, ^2^*J*_C-F_ = 36.2 Hz, C(4)), 138.7 (s, *i*-C), 160.9 (s, C=O). ^19^F NMR (CDCl_3_, 565 MHz): δ −68.5 (s, CF_3_). IR (neat): *ν* 1692 and 1644 (C=O), 1491, 1446, 1331, 1193, 1122 (CF_3_) cm^−1^. ESI-MS (*m*/*z*): 320.3 (31, [*M*{^37^Cl}+H]^+^), 318.2 (100, [*M*{^35^Cl}+H]^+^). C_13_H_11_ClF_3_N_3_O (317.7): calcd. C 49.15, H 3.49, N 13.23; found: C 49.22, H 3.68, N 13.10.

(*S*)-2-(2,4-Dichlorophenyl)-4-trifluoromethyl-6,7,8,8a-tetrahydropyrrolo [1,2-*d*][1,2,4]triazin-1(2*H*)-one (**7e**): In order to accelerate the second cyclization step, the crude reaction mixture was heated in an oil bath (60 °C) for three days; CC (SiO_2_, petroleum ether/EtOAc 4:1), 176 mg (50%). Yellow solid, m.p. 107–108 °C. [α]_D_^20^ = −6.8 (*c* 0.13, CHCl_3_). ^1^H NMR (CDCl_3_, 600 MHz): δ 2.04–2.12 (m, 2 H, 7-H_2_), 2.25–2.32 (m, 1 H, 8-H), 2.43–2.48 (m, 1 H, 8-H), 3.68–3.78 (m, 2 H, 6-H_2_), 4.17 (pseudo-t, *J* ≈ 7.9 Hz, 1 H, 8a-H), 7.32 (m_c_, 2 H), 7.47 (t, *J* = 1.2 Hz, 1 H). ^13^C NMR (CDCl_3_, 151 MHz): δ 24.0 (t, C(7)), 28.2 (t, C(8)), 48.8 (q, ^4^*J*_C-F_ = 2.5 Hz, C(6)), 58.1 (d, C(8a)), 118.5 (q, ^1^*J*_C-F_ = 275.7 Hz, CF_3_), 128.2, 130.2, 130.4 (3 d, 3 CH), 133.3, 135.2, 136.5 (3 s, 3 *i*-C), 137.6 (q, ^2^*J*_C-F_ = 36.2 Hz, C(4)), 161.0 (s, C=O). ^19^F NMR (CDCl_3_, 565 MHz): δ −68.4 (s, CF_3_). IR (neat): *ν* 1689 and 1640 (C=O), 1480, 1443, 1228, 1189, 1160–1118 (CF_3_) cm^−1^. ESI-MS (*m*/*z*): 353.5 (24), 352.4 (100). C_13_H_10_Cl_2_F_3_N_3_O (352.1): calcd. C 44.34, H 2.86, N 11.93; found: C 44.42, H 2.96, N 12.04.

(*S*)-2-Phenyl-4-trifluoromethyl-6,7,8,8a-tetrahydropyrrolo [1,2-*d*][1,2,4]triazin-1(2*H*)-one (**7f**): CC (SiO_2_, petroleum ether/EtOAc 4:1), 170 mg (60%). Yellow solid, m.p. 61–63 °C. [α]_D_^20^ = +22.6 (*c* 0.18, CHCl_3_). ^1^H NMR (CDCl_3_, 600 MHz): δ 2.02–2.11 (m, 2 H, 7-H_2_), 2.25–2.32 (m, 1 H, 8-H), 2.42–2.47 (m, 1 H, 8-H), 3.67–3.75 (m, 2 H, 6-H_2_), 4.14 (dd, *J* = 6.9, 8.8 Hz, 1 H, 8a-H), 7.25–7.28, 7.38–7.41, 7.54–7.56 (3 m, 1 H, 2 H, 2 H). ^13^C NMR (CDCl_3_, 151 MHz): δ 24.1 (t, C(7)), 28.4 (t, C(8)), 48.7 (q, ^4^*J*_C-F_ = 2.3 Hz, C(6)), 58.2 (d, C(8a)), 118.7 (q, ^1^*J*_C-F_ = 275.6 Hz, CF_3_), 124.5, 127.0, 128.7 (3 d, 5 CH), 137.3 (q, ^2^*J*_C-F_ = 36.1 Hz, C(4)), 140.2 (s, *i*-C), 160.9 (s, C=O). ^19^F NMR (CDCl_3_, 565 MHz): δ −68.4 (s, CF_3_). IR (neat): *ν* 1685 and 1640 (C=O), 1457, 1312, 1199, 1133 (CF_3_), 1102 cm^−1^. ESI-MS (*m*/*z*): 284.2 (32, [*M*+H]^+^), 205.2 (100, [*M*–Ph]^+^). C_13_H_12_F_3_N_3_O (283.2): calcd. C 55.12, H 4.27, N 14.84; found: C 55.41, H 4.44, N 14.59.

(*S*)-2-(4-Tolyl)-4-trifluoromethyl-6,7,8,8a-tetrahydropyrrolo [1,2-*d*][1,2,4]triazin-1(2*H*)-one (**7g**): CC (SiO_2_, petroleum ether/CH_2_Cl_2_ 1:3), 166 mg (56%). Light gray solid, m.p. 78–80 °C. [α]_D_^20^ = +22.7 (*c* 0.16, CHCl_3_). ^1^H NMR (CDCl_3_, 600 MHz): δ 2.03–2.11 (m, 2 H, 7-H_2_), 2.25–2.32 (m, 1 H, 8-H), 2.35 (s, 3 H, Me), 2.42–2.47 (m, 1 H, 8-H), 3.67–3.76 (m, 2 H, 6-H_2_), 4.13 (dd, *J* = 6.9, 8.8 Hz, 1 H, 8a-H), 7.18–7.20, 7.39–7.41 (2 m, 2 H each). ^13^C NMR (CDCl_3_, 151 MHz): δ 21.2 (q, Me), 24.1 (t, C(7)), 28.5 (t, C(8)), 48.7 (q, ^4^*J*_C-F_ = 2.4 Hz, C(6)), 58.2 (d, C(8a)), 118.7 (q, ^1^*J*_C-F_ = 275.6 Hz, CF_3_), 124.5, 129.4 (2 d, 4 CH), 137.0 (s, *i*-C), 137.2 (q, ^2^*J*_C-F_ = 36.1 Hz, C(4)), 137.7 (s, *i*-C), 160.9 (s, C=O). ^19^F NMR (CDCl_3_, 565 MHz): δ −68.4 (s, CF_3_). IR (neat): *ν* 1687 and 1644 (C=O), 1511, 1444, 1198, 1120 (CF_3_), 820 cm^−1^. ESI-MS (*m*/*z*): 299.2 (100, [*M*+2H]^+^). C_14_H_14_F_3_N_3_O (297.1): calcd. C 56.56, H 4.75, N 14.14; found: C 56.67, H 4.84, N 13.90.

(*S*)-2-(4-Benzyloxyphenyl)-4-trifluoromethyl-6,7,8,8a-tetrahydropyrrolo [1,2-*d*][1,2,4]triazin-1(2*H*)-one (**7h**): CC (SiO_2_, petroleum ether/EtOAc 4:1), 183 mg (47%). Colorless solid, m.p. 97–99 °C. [α]_D_^20^ = +35.0 (*c* 0.21, CHCl_3_). ^1^H NMR (CDCl_3_, 600 MHz): δ 2.02–2.10 (m, 2 H, 7-H_2_), 2.24–2.31 (m, 1 H, 8-H), 2.42–2.47 (m, 1 H, 8-H), 3.66–3.74 (m, 2 H, 6-H_2_), 4.13 (dd, *J* = 6.9, 8.9 Hz, 1 H, 8a-H), 5.07 (s, 2 H, OCH_2_), 6.97–7.00, 7.31–7.34, 7.37–7.39, 7.41–7.44 (4 m, 2 H, 1 H, 2 H, 4 H). ^13^C NMR (CDCl_3_, 151 MHz): δ 24.1 (t, C(7)), 28.5 (t, C(8)), 48.7 (q, ^4^*J*_C-F_ = 2.6 Hz, C(6)), 58.2 (d, C(8a)), 70.3 (t, OCH_2_), 115.0 (d, 2 CH), 118.7 (q, ^1^*J*_C-F_ = 275.6 Hz, CF_3_), 126.1, 127.6, 128.1, 128.7 (4 d, 7 CH), 133.5, 137.0 (2 s, 2 *i*-C), 137.2 (q, ^2^*J*_C-F_ = 36.2 Hz, C(4)), 157.6 (s, *i*-C), 160.9 (s, C=O). ^19^F NMR (CDCl_3_, 565 MHz): δ −68.4 (s, CF_3_) ppm. IR (neat): *ν* 1685 and 1644 (C=O), 1506, 1450, 1338, 1241, 1133 (CF_3_), 1018 cm^−1^. ESI-MS (*m*/*z*): 412.4 (77, [*M*+Na]^+^), 390.4 (100, [*M*+H]^+^). C_20_H_18_F_3_N_3_O_2_ (389.4): calcd. C 61.69, H 4.66, N 10.79; found: C 61.50, H 4.83, N 10.56.

Synthesis of (*S*)-4-Methyl-5-phenyl-1-(4-tolyl)-3-trifluoromethyl-4,5-dihydro-1,2,4-triazin-6(1*H*)-one ((*S*)-**6r**)): A solution of 1,2,4-triazin-6(1*H*)-one (*S*)-**6q** (0.5 mmol, 167 mg) in anhydrous MeOH (5.0 mL) was added dropwise to a vigorously stirred solution of sodium methoxide (5.0 mmol, 270 mg) in dry MeOH (25 mL) under argon, at room temperature. Then MeI (5.0 mmol, 705 mg) was added to the resulting mixture and the stirring was continued for 24 **h**. After the solvents were removed in vacuo, the residue was washed with EtOAc (3 × 20 mL). The organic layers were combined, the solvent was removed, and the crude product was purified by CC (SiO_2_, DCM) to give (*S*)-**6r** (101 mg, 58%). Colorless solid, m.p. 92–93 °C. [α]_D_^20^ = +5.2 (*c* 0.24, CHCl_3_). ^1^H NMR (CDCl_3_, 600 MHz): δ 2.34 (s, 3 H, Me), 3.05 (s, 3 H, NMe), 4.94 (s, 1 H, 5-H), 7.17–7.19, 7.36–7.44 (2 m, 2 H, 7 H). ^13^C NMR (CDCl_3_, 151 MHz): δ 21.2 (q, Me), 36.2* (q, *J*_C-F_ = 3.4 Hz, NMe), 66.0 (d, C(5)), 118.9 (q, ^1^*J*_C-F_ = 275.9 Hz, CF_3_), 124.4, 126.9, 129.36, 129.43, 129.5 (5 d, 9 CH), 135.9 (s), 136.5 (q, ^2^*J*_C-F_ = 34.3 Hz, C(3)), 137.1, 137.6 (2 s), 158.6 (s, C=O); *through-space C–F coupling observed. ^19^F NMR (CDCl_3_, 565 MHz): δ −66.1 (s, CF_3_) ppm. IR (neat): *ν* 1681 and 1655 (C=O), 1513, 1416, 1364, 1238, 1182, 1126 (CF_3_), 1077 cm^−1^. ESI-MS (*m*/*z*): 370.1 (100, [*M*+Na]^+^), 348.1 (80, [*M*+H]^+^), 318.2 (94). C_18_H_16_F_3_N_3_O (347.1): calcd. C 62.24, H 4.64, N 12.10; found: C 61.99, H 4.51, N 12.08. A sample of *rac*-**6r** (111 mg, 64%; 0.5 mmol scale) was obtained in an analogous manner starting with *rac*-**6q**. Colorless crystals, m.p. 93–95 °C. The NMR spectra (^1^H and ^13^C) of *rac*-**6r** were in accordance with those of (*S*)-**6r**.

Synthesis of 1-(4-tolyl)-3-trifluoromethyl-1,2,4-triazin-6(1*H*)-one (**6s**): A mixture of 4,5-dihydro-1,2,4-triazinone **6g** (0.5 mmol, 128.5 mg), K_3_Fe(CN)_6_ (3.0 mmol, 987 mg), aqueous solution of Na_2_CO_3_ (0.5M, 10 mL), and Et_4_NBr (15 mol%) in CH_2_Cl_2_ (10 mL) was vigorously stirred at room temperature for 4 h (monitored on TLC). The resulting mixture was extracted with CH_2_Cl_2_ (3 × 10 mL), the combined organic layers were dried over anh. Na_2_SO_4_, filtered and the solvents were removed under reduced pressure. The crude product was purified by standard CC (SiO_2_, CH_2_Cl_2_) to give **6s** (99 mg, 78%). Colorless solid, m.p. 80–82 °C. ^1^H NMR (CDCl_3_, 600 MHz): δ 2.36 (s, 3 H, Me), 7.25, 7.57 (2 d_br_, *J* ≈ 8.3 Hz, 2 H each), 8.47 (s, 1 H, 5-H). ^13^C NMR (CDCl_3_, 151 MHz): δ 21.4 (q, Me), 119.2 (q, ^1^*J*_C-F_ = 273.6 Hz, CF_3_), 123.9, 129.9 (2 d, 4 CH), 136.6, 140.2 (2 s, 2 *i*-C), 140.6 (q, ^2^*J*_C-F_ = 39.0 Hz, C(3)), 152.6 (s, C=O), 161.0 (d, C(5)). ^19^F NMR (CDCl_3_, 565 MHz): δ −69.7 (s, CF_3_). IR (neat): *ν* 1674 (C=O), 1391, 1346, 1156, 1088 (CF_3_) cm^−1^. ESI-MS (*m*/*z*): 256.1 (100, [*M*+H]^+^). C_11_H_8_F_3_N_3_O (255.1): calcd. C 51.77, H 3.16, N 16.47; found: C 51.59, H 3.23, N 16.43.

General procedure for catalytic hydrogenation reactions: A solution of the corresponding triazinone (**7a** or **7h**, 0.5 mmol) in EtOH (10 mL) was added Pd/C (5.0 mmol), and the resulting mixture was vigorously shaken in the atmosphere of H_2_ (3 atm) for the required time. The mixture was filtered through Celite, washed with EtOH (5 mL), and the solvents were removed under reduced pressure. The resulting mixture was filtered through a short plug of silica (CC) to give the spectroscopically pure product.

(*S*)-2-(4-Aminophenyl)-4-trifluoromethyl-6,7,8,8a-tetrahydropyrrolo [1,2-*d*][1,2,4]-triazin-1(2*H*)-one (**7i**): Reaction time: 3 h; CC (SiO_2_, silica was washed with 5% Et_3_N in EtOAc prior to use; petroleum ether/EtOAc 4:1), 132 mg (89%). Thick light orange oil. [α]_D_^20^ = +43.1 (*c* 0.15, CHCl_3_). ^1^H NMR (CDCl_3_, 600 MHz): δ 2.03–2.10 (m, 2 H, 7-H_2_), 2.23–2.32 (m, 1 H, 8-H), 2.41–2.46 (m, 1 H, 8-H), 3.65–3.74 (m, 2 H, 6-H_2_), 3.70 (s_br_, 2 H, NH_2_), 4.12 (dd, *J* = 6.9, 8.8 Hz, 1 H, 8a-H), 6.66–6.96, 7.24–7.27 (2 m, 2 H each). ^13^C NMR (CDCl_3_, 151 MHz): δ 24.1 (t, C(7)), 28.5 (t, C(8)), 48.7 (q, ^4^*J*_C-F_ = 2.1 Hz, C(6)), 58.2 (d, C(8a)), 115.1 (d, 2 CH), 118.7 (q, ^1^*J*_C-F_ = 275.9 Hz, CF_3_), 126.1 (d, 2 CH), 131.5 (s, *i*-C), 137.0 (q, ^2^*J*_C-F_ = 36.1 Hz, C(4)), 145.6 (s, *i*-C), 160.8 (s, C=O). ^19^F NMR (CDCl_3_, 565 MHz): δ −68.3 (s, CF_3_). IR (neat): *ν* 3362 (NH), 1674 and 1633 (C=O), 1513, 1446, 1193, 1140–1095 (CF_3_) cm^−1^. ESI-MS (*m*/*z*): 299.3 (100, [*M*+H]^+^), 279.3 (30).

(*S*)-2-(4-Hydroxyphenyl)-4-trifluoromethyl-6,7,8,8a-tetrahydropyrrolo [1,2-*d*][1,2,4]-triazin-1(2*H*)-one (**7j**): Reaction time: 16 h; CC (SiO_2_, hexanes/EtOAc 2:3), 100 mg (67%). Colorless solid, m.p. 94–96 °C. [α]_D_^20^ = +23.2 (*c* 0.18, CHCl_3_). ^1^H NMR (CDCl_3_, 600 MHz): δ 2.02–2.10 (m, 2 H, 7-H_2_), 2.24–2.31 (m, 1 H, 8-H), 2.42–2.47 (m, 1 H, 8-H), 3.67–3.76 (m, 2 H, 6-H_2_), 4.15 (dd, *J* = 6.9, 8.9 Hz, 1 H, 8a-H), 5.69 (s_br_, 1 H, OH), 6.74–6.77, 7.28–7.30 (2 m, 2 H each). ^13^C NMR (CDCl_3_, 151 MHz): δ 24.1 (t, C(7)), 28.5 (t, C(8)), 48.7 (q, ^4^*J*_C-F_ = 2.5 Hz, C(6)), 58.2 (d, C(8a)), 115.8 (d, 2 CH), 118.7 (q, ^1^*J*_C-F_ = 275.6 Hz, CF_3_), 126.5 (d, 2 CH), 133.0 (s, *i*-C), 137.4 (q, ^2^*J*_C-F_ = 36.1 Hz, C(4)), 155.0 (s, *i*-C), 161.1 (s, C=O). ^19^F NMR (CDCl_3_, 565 MHz): δ −68.4 (s, CF_3_). IR (neat): *ν* 3317 (OH), 1666 and 1636 (C=O), 1513, 1446, 1341, 1189–1122 (CF_3_), 835 cm^−1^. ESI-MS (*m*/*z*): 300.2 (100, [*M*+H]^+^), 298.2 (59).

General procedure for synthesis of hydrazonoyl bromides **11**: Following the general literature protocol [25], arylhydrazone **14** (1.0 mmol) was dissolved in dry DMF (3 mL), the solution was cooled to 0 °C, then solid NBS (1.05 mmol, 187 mg) was added and stirring was continued at this temperature. After the starting hydrazone was fully consumed (TLC monitoring, typically ca. 2 h), the resulting mixture was extracted with H_2_O/Et_2_O 1:1 mixture (20 mL), the organic layer was washed with H_2_O (3 × 10 mL), dried over anh. Na_2_SO_4_, filtered and the solvents were removed in vacuo. Crude products were purified by column chromatography.

*N*-(3-Nitrophenyl)-trifluoroacetohydrazonoyl bromide (**11b**): reaction time: 2h; CC (SiO_2_, petroleum ether/CH_2_Cl_2_ 3:2), 265 mg (85%). Yellow solid, m.p. 110–111 °C. ^1^H NMR (CDCl_3_, 600 MHz): δ 7.49–7.52, 7.87–7.91, 7.99–8.01 (3 m, 2 H, 1 H, 1 H), 8.24 (s_br_, 1 H, NH). ^13^C NMR (CDCl_3_, 151 MHz): δ 107.1 (q, ^2^*J*_C-F_ = 44.0 Hz, =*C*CF_3_), 109.2, 117.7 (2 d, 2 CH), 118.2 (q, ^1^*J*_C-F_ = 272.1 Hz, CF_3_), 120.0, 130.7 (2 d, 2 CH), 142.7, 149.4 (2 s, 2 *i*-C). ^19^F NMR (CDCl_3_, 565 MHz): δ −66.6 (s, CF_3_). IR (neat): *ν* 3258 (NH), 1614, 1524, 1346, 1304, 1238, 1121–1075 (CF_3_) cm^−1^. (-)-ESI-MS (*m*/*z*): 311.9 (100, [*M*{^81^Br}–H]^−^), 309.9 (99, [*M*{^79^Br}–H]^−^). C_8_H_5_BrF_3_N_3_O_2_ (312.0): calcd. C 30.79, H 1.62, N 13.47; found: C 30.95, H 1.88, N 13.65.

*N*-(2,4-Dichlorophenyl)-trifluoroacetohydrazonoyl bromide (**11e**): Reaction time: 3 h; CC (SiO_2_, hexanes), 332 mg (99%). Thick yellow oil. ^1^H NMR (CDCl_3_, 600 MHz): δ 7.24 (dd, *J* = 2.3, 8.8 Hz, 1 H), 7.35 (d, *J* = 2.3 Hz, 1 H), 7.42 (d, *J* = 8.8 Hz, 1 H), 8.52 (s_br_, 1 H, NH). ^13^C NMR (CDCl_3_, 151 MHz): δ 107.8 (q, ^2^*J*_C-F_ = 43.9 Hz, =*C*CF_3_), 116.5 (d, CH), 118.3 (q, ^1^*J*_C-F_ = 271.9 Hz, CF_3_), 119.2, 127.8 (2 s, 2 *i*-C), 128.6, 129.2 (2 d, 2 CH), 136.6 (s, *i*-C). ^19^F NMR (CDCl_3_, 565 MHz): δ −66.6 (s, CF_3_). IR (neat): *ν* 3314 (NH), 1595, 1506, 1327, 1282, 1124, 1208, 1133 (CF_3_), 969 cm^−1^. (-)-ESI-MS (*m*/*z*): 336.7 (38), 335.6 (11), 334.7 (100), 332.8 (63). C_8_H_4_BrCl_2_F_3_N_2_ (335.9): calcd. C 28.60, H 1.20, N 8.34; found: C 28.42, H 1.36, N 8.02.

General procedure for synthesis of trifluoroacetaldehyde arylhydrazones **14**: Following the literature protocol [52], a mixture of arylhydrazine hydrochloride (1.0 mmol), excess fluoral hydrate (ca. 3.0 mmol), and freshly activated powdered molecular sieves 4Å (ca. 450 mg) in MeOH (3.5 mL) was heated in a closed ampoule in an oil bath (75 °C) overnight. The solution was cooled to room temperature and filtered through a short pad of Celite, which was washed with several portions of CH_2_Cl_2_ (4 × 5 mL). The combined organic layers were washed with H_2_O (10 mL), then with 5%-aqueous solution of NaHCO_3_ (10 mL), and dried over Na_2_SO_4_. The solid inorganics were filtered off and the solvents were removed under reduced pressure (cold bath). The crude products were purified by standard CC to give spectroscopically pure materials, which were used for the next step without further purification.

Trifluoroacetaldehyde 3-nitrophenylhydrazone (**14b**): CC (SiO_2_, petroleum ether/CH_2_Cl_2_ 3:2), 152 mg (65%). Yellow solid, m.p. 156–158 °C. ^1^H NMR (CDCl_3_, 600 MHz): δ 7.08 (qd, *J*_H-H_ = 1.4 Hz, *J*_H-F_ = 3.9 Hz, 1 H, =CHCF_3_), 7.43 (ddd, *J* = 1.2, 2.2, 8.2 Hz, 1 H), 7.47 (t_br_, *J* ≈ 8.0 Hz, 1 H), 7.83 (ddd, *J* = 1.2, 2.2, 7.9 Hz, 1 H), 7.91 (t, *J* = 2.2 Hz, 1 H), 8.18 (s_br_, 1 H, NH). ^13^C NMR (CDCl_3_, 151 MHz): δ 108.3, 116.9, 119.3 (3 d, 3 CH), 120.7 (q, ^1^*J*_C-F_ = 269.8 Hz, CF_3_), 125.2 (q, ^2^*J*_C-F_ = 39.6 Hz, =*C*CF_3_), 130.5 (d, CH), 144.0, 149.4 (2 s, 2 *i*-C). ^19^F NMR (CDCl_3_, 565 MHz): δ −65.9 (d, *J*_H-F_ = 3.9 Hz, CF_3_). IR (neat): *ν* 3302 (NH), 1610, 1558, 1342, 1290, 1245, 1118 (CF_3_), 1074 cm^−1^. (-)-ESI-MS (*m*/*z*): 231.8 (100, [*M*–H]^−^). C_8_H_6_F_3_N_3_O_2_ (233.0): calcd. C 41.21, H 2.59, N 18.02; found: C 41.21, H 2.72, N 18.03.

Trifluoroacetaldehyde 2,4-dichlorophenylhydrazone (**14e**): CC (SiO_2_, petroleum ether/CH_2_Cl_2_ 3:2), 202 mg (79%). Light orange oil. ^1^H NMR (CDCl_3_, 600 MHz): δ 7.12 (qd, *J*_H-H_ = 1.4 Hz, *J*_H-F_ = 3.8 Hz, 1 H, =C*H*CF_3_), 7.22 (dd, *J* = 2.3, 8.8 Hz, 1 H), 7.31 (d, *J* = 2.3 Hz, 1 H), 7.46 (d, *J* = 8.8 Hz, 1 H), 8.33 (s_br_, 1 H, NH). ^13^C NMR (CDCl_3_, 151 MHz): δ 115.9 (d, CH), 118.2 (s, *i*-C), 120.8 (q, ^1^*J*_C-F_ = 269.7 Hz, CF_3_), 125.6 (q, ^2^*J*_C-F_ = 39.3 Hz, =*C*CF_3_), 126.8 (s, *i*-C), 128.5, 129.0 (2 d, 2 CH), 137.7 (s, *i*-C). ^19^F NMR (CDCl_3_, 565 MHz): δ −65.9 (d, *J*_H-F_ = 3.8 Hz, CF_3_). IR (neat): *ν* 3354 (NH), 1591, 1521, 1357, 1279, 1234, 1115 (CF_3_), 1051, 913, 816 cm^−1^. (-)-ESI-MS (*m*/*z*): 254.8 (100, [*M*–H]^−^). C_8_H_5_Cl_2_F_3_N_2_ (256.0): calcd. C 37.38, H 1.96, N 10.90; found: C 37.36, H 2.20, N 10.97.

## 3. Results and Discussion

**Scheme 2:**
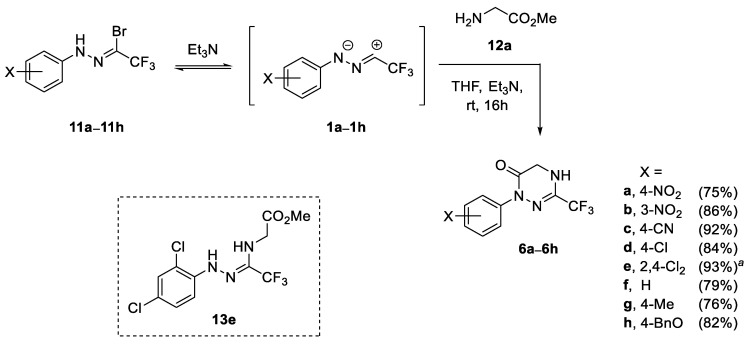
Synthesis of 1,2,4-triazin-6(1*H*)-ones **6a**–**6h** derived from methyl glycinate (scope of nitrile imines **1**) and the structure of intermediate **13e**; *^a^* crude reaction mixture was refluxed for 2 h.

Based on our experience in the chemistry of (3+2)-cycloaddition reactions of nitrile imines **1**, the first experiments were carried out in dry THF solutions, under inert atmosphere, at room temperature, using hydrazonoyl bromides **11** as the source of nitrile imines and excess Et_3_N as a base triggering the dehydrohalogenation reaction [25,26,27,28]. For a test experiment, *N*-(4-nitrophenyl)-trifluoroacetohydrazonoyl bromide (**11a**) and methyl glycinate (**12a**, as hydrochloride) were selected as reaction partners, and after the addition of a base (3.0 equiv.), the slow consumption of the nitrile imine precursor was observed (according to TLC monitoring) to give after 16 h the expected (3+3)-cycloadduct **6a**, which was isolated by flash chromatography in a fair 59% yield (Scheme 2). Brief optimization of the reaction conditions revealed that increasing the amount of Et_3_N (8.0 equiv.) enhanced the yield of isolated product **6a** (75%) formed as the only product. On the other hand, neither the change of the base (Cs_2_CO_3_, pyridine, DBU), nor the type of solvent used (CH_2_Cl_2_, toluene) resulted in remarkable change of the chemical yield of the studied reaction. Prompted by the often-observed positive effects of mechanochemical activation on reaction outcomes, (e.g., remarkable shortening of reaction times, higher yields, etc.) [53,54,55], the (3+3)-annulation reaction of **11a** and **12a** was also tested under ball-mill conditions (steel balls, ø 7 mm, 25 Hz). However, in this case, the formation of viscous mixture was observed upon the reaction progress, which enabled effective ball-milling and the reaction could not be completed. Moreover, the use of three-fold excess of glycinate **12a** (with respect to bromide **11a**) was necessary to obtain the desired product **6a** in a comparable yield (77%) and the remarkably shorter reaction time of 2 h, and for these reasons we waved on the mechanochemical approach.

The structure of the isolated product **6a** was confirmed based on NMR methods; for example, in ^1^H NMR spectrum, a broadened doublet (*J* ≈ 1.5 Hz) attributed to 5-H_2_ was found at δ 4.30, along with absorption (s_br_) of the NH (δ 5.31), and the characteristic set of signals (two d_br_ located at δ 7.92 and 8.27, *J* ≈ 9.2 Hz each) of the C_6_H_4_NO_2_ group. Furthermore, two diagnostic quartets attributed to the CF_3_ group, and the C-3 atom were found at δ 118.2 (^1^*J*_C-F_ = 275.2 Hz) and δ 137.3 (^2^*J*_C-F_ = 37.8 Hz), respectively, whereas the absorption (s) of the amide-type C=O group was found at δ 158.1. Finally, the ^19^F NMR indicated the presence of a single CF_3_ group (singlet at δ −70.6), while the ESI-MS, supplemented by combustion analysis, confirmed the molecular formula of C_10_H_7_F_3_N_4_O_3_. Based on the collected data, the structure of isolated product was established as the hitherto unknown 1-(4-nitrophenyl)-3-trifluoromethyl-4,5-dihydro-1,2,4-triazin-6(1*H*)-one (**6a**).

**Scheme 3:**
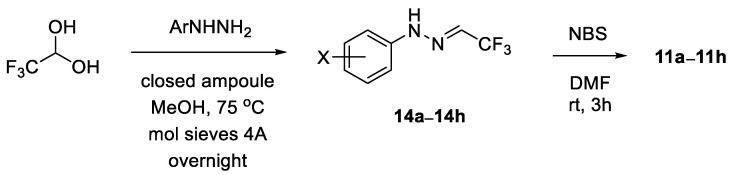
Synthesis of hydrazonoyl bromides **11a**–**11h**.

With the optimized reaction conditions in hand, a series of variously substituted nitrile imines **1b**–**1h** were checked in the reaction with glycinate **12a** (Scheme 2). The required nitrile imine precursors of type **11** were prepared according to the general literature protocols by condensation of arylhydrazines with fluoral hydrate [52], followed by NBS-mediated bromination of the first formed hydrazones **14** (Scheme 3) [25]. As shown in Scheme 2, the expected products were formed in high yields (74–93%), irrespective of the electronic nature of the substituent X. These observations nicely correspond to the previous results reported by Dalloul for reactions of some non-fluorinated nitrile imines with methyl glycinate and other amino acid esters [56,57]. Notably, in the case of highly electron-deficient nitrile imine **1e** functionalized at the *N*-termini with 2,4-dichlorophenyl group, the formation of a mixture of the target material **6e** and the first formed acyclic adduct **13e** in ca. 3:2 ratio was observed, which nicely evidenced the stepwise character of the studied (3+3)-annulation reaction. Therefore, in the next attempt, the resulting crude mixture was additionally refluxed for 2 h in order to accelerate the second ring-closure step, and after standard work-up, the product **6e** was isolated in excellent yield (93%).

**Scheme 4:**
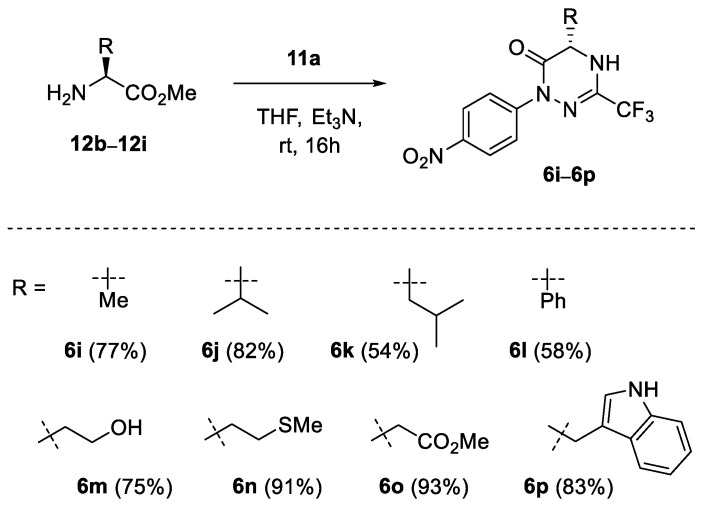
Synthesis of chiral 1,2,4-triazin-6(1*H*)-ones **6i**–**6q** derived from nitrile imine **1a**; scope of α-amino esters.

**Scheme 5:**
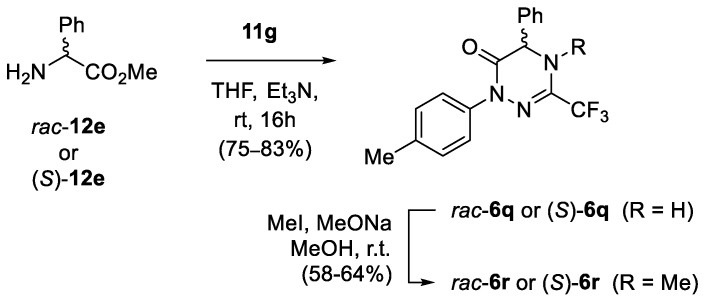
Synthesis of racemic and enantiopure phenylglycine-derived 1,2,4-triazin-6-ones *rac*-**6q** and (*S*)-**6q**, and their transformation into *N*-methylated analogues **6r**.

Next, a series of enantiopure α-substituted (*S*)-amino esters **12b**–**12i** derived from alanine, valine, leucine, phenylglycine, serine, methionine, aspartic acid, and tryptophan, respectively, were examined in reaction with selected hydrazonoyl bromides **11a** and **11g** to afford the expected optically active products **6i**–**6q** (Scheme 4 and Scheme 5). Thus, along with simple alkyl and aryl substituents in **6i**–**6l**, functional groups such as hydroxy (**6m**), thioether (**6n**) and secondary amine (**6p**) could also be efficiently introduced. Notably, in the case of methyl aspartate (**12h**), the presence of the additional CO_2_Me group did not interfere with the subsequent cyclisation step, and the 6-membered product **6o** was formed exclusively in excellent yield (93%).

In order to check the optical purity of the resulting products, phenylglycine-derived product **6q** was selected for a more detailed examination, and a sample of racemic 1,2,4-triazinone *rac*-**6q** was prepared as a reference compound (Scheme 5). Unfortunately, neither chiral-HPLC analysis of pure samples of **6q** nor ^1^H NMR measurements of their diastereomeric 1:1 mixtures with (+)-(*S*)-mandelic acid and with (+)-(*R*)-(*tert*-butyl)(phenyl)phosphonothioic acid [58] selected as chiral solvating agents was successful. In addition, the attempted derivatization of 1,2,4-triazin-6-ones **6q** using enantiopure acid chloride derived from (*S*)-naproxen was in vain. Nevertheless, the treatment of **6q** with strongly electrophilic MeI under basic conditions enabled fully regioselective methylation at N(4) to afford the expected products (*S*)-**6r** and *rac*-**6r** isolated in 58% and 64% yield, respectively. Subsequent HPLC analysis of both samples proved the optical purity of the product derived from chiral methyl phenylglycinate (*S*)-**12e** (for details, see Supporting Information). Hence, taking into account the high susceptibility for epimerization of the phenylglycine-derived compounds under basic conditions, we assumed that all the other products in the series (**6i**–**6q**) were optically pure as well.

The ^13^C NMR spectrum of *N*-methylated product **6r** also deserves a brief comment. Along with the expected diagnostic quartets located at δ 118.9 (^1^*J*_C-F_ = 275.9 Hz) and δ 136.5 (^2^*J*_C-F_ = 34.3 Hz) attributed to the CF_3_ group and the C(3) atom, respectively, an additional quartet of the *N*-Me at δ 36.2 (*J*_C-F_ = 3.4 Hz) was found. Apparently, the close proximity of the CF_3_ and the Me groups results in the observed C–F through-space coupling.

**Scheme 6:**
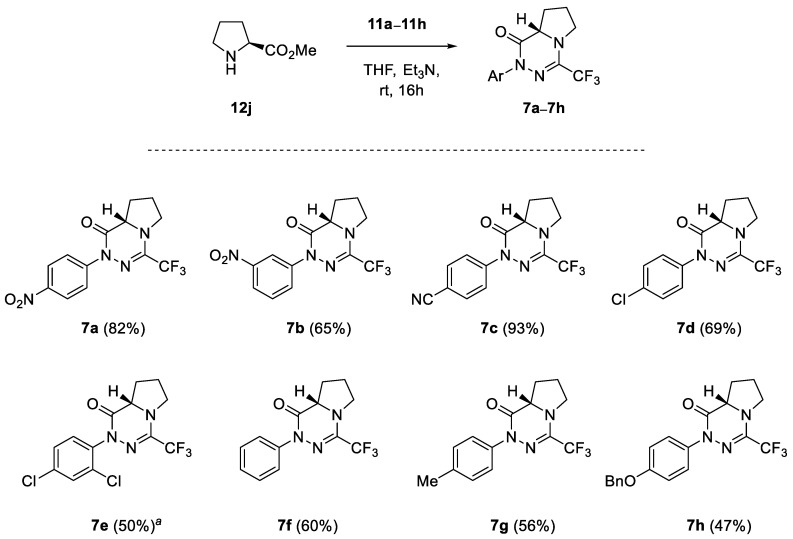
Synthesis of l-proline-derived bicyclic 1,2,4-triazinones **7a**–**7h**; crude reaction mixture was heated at 60 °C for 3 d.

In addition to a series of experiments performed with the primary α-amino esters **12a**–**12i**, (S)-proline methyl ester (**12j**) was also involved in the study as a model secondary amino-compound. As shown in Scheme 6, (3+3)-annulation of **12j** with nitrile imines of type **1** carried out under the analogous reaction conditions provided the expected bicyclic products **7a**–**7h**, which were generally isolated in fair yield (47–93%). Similar to the cyclo-condensation of 2,4-dichlorophenyl-functionalized nitrile imine **1e** with methyl glycinate (Scheme 1), the reaction of **12j** with **1e** also provided the respective amidrazone intermediate, which required prolonged heating in THF (3 d, 60 °C) to afford the target bicyclic material **7e** (50%).

**Scheme 7:**
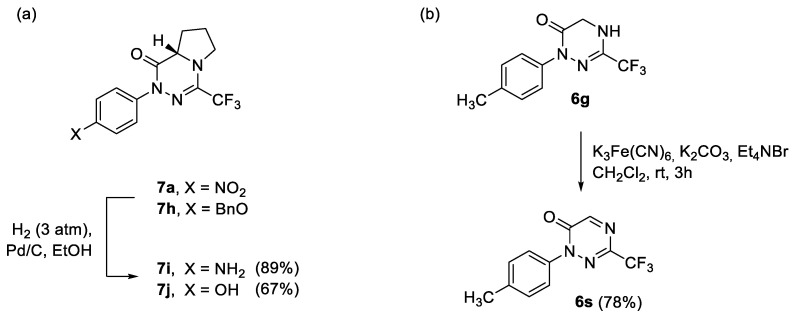
Selected transformations of title heterocycles: (**a**) synthesis of amino- and hydroxy-functionalized products **7i** and **7j** through catalytic hydrogenation of **7a** and **7h**, respectively; (**b**) selective oxidation of **6g** with K_3_Fe(CN)_6_ under PTC-conditions leading to 1,2,4-triazin-6-one **6s**.

Having in hand two series of 3-trifluoromethylated 1,2,4-triazin-6-ones **6** and **7**, selected functional group interconversions were carried out to demonstrate the robustness of the core heterocycle. Thus, NH_2_ and OH groups were easily accessed by medium-pressure (3 atm) catalytic hydrogenation of the NO_2_ and BnO groups in **7a** and **7h**, respectively, and the expected products **7i** (89%) and **7j** (67%) were isolated in high yields (Scheme 7). On the other hand, with the treatment of the 4,5-dihydro-1,2,4-triazine derivative **6g** with an aqueous solution of K_3_Fe(CN)_6_, in the presence of tetraethylammonium bromide as a phase transfer catalyst, the smooth oxidation of the N(4)–C(5) bond was observed to yield fairly stable 1,2,4-triazin-6-one **6s** (78%) identified as the only product. The structure of **6s** was confirmed based on ^1^H and ^13^C NMR spectra supplemented by 2D measurements (HMQC); particularly, characteristic low-field shifted absorptions attributed to C(5)-H unit, i.e., a singlet at δ 8.47 in ^1^H NMR and a signal at δ 161.0 in ^13^C NMR, were observed.

Finally, prompted by recent reports on anticancer properties of some fluorinated and non-fluorinated 1,2,4-triazinone-based materials [43,44,45,46,47,48,49,50,51], the cytotoxicity of 3-CF_3_-1,2,4-triazin-6(1*H*)-ones **6a**–**6q** and **7a**–**7h** obtained in this study was checked against human promyelocytic leukemia (HL-60) and breast cancer adenocarcinoma (MCF-7) cell lines (for details, see Supplementary Materials). However, most of the examined samples showed no mentionable cytotoxicity; for example, all compounds in glycine-derived series **6a**–**6h** proved inactive irrespective of the type of substituent X, whereas in the case of chiral representatives **6i**–**6q** only for the analogue functionalized with H-donor/acceptor 2-hydroxyethyl group (compound **6m**), a moderate (IC_50_ = 23.0 μM) activity against HL-60 line was observed. For the proline-derived analogues **7a**–**7h,** the best result was noticed for benzonitrile-functionalized analogue **7c** with only low cytotoxicity of IC_50_ = 45.4 μM against HL-60 cell line.

## 4. Conclusions

In the presented study, the synthesis of a series of 4,5-dihydro-1,2,4-triazin-6(1*H*)-ones functionalized with the CF_3_ group is reported. The devised protocol is based on the (3+3)-annulation of methyl esters derived from natural α-amino acids with in situ generated trifluoroacetonitrile imines applied as reactive 1,3-dipolar reaction partners. Notably, starting with chiral α-amino esters, no racemization occurred under the optimized reaction conditions, and the expected enantiopure materials were isolated as the only products. Furthermore, with the application of methyl l-prolinate as a model secondary amino ester, the respective fused 1,2,4-triazinones were obtained. The selected functional group interconversions performed under catalytic hydrogenation or mild PTC-oxidation conditions demonstrated remarkable stability of the core heterocycle. Thus, the presented method offers straightforward access to the desired heterocyclic system functionalized not only with simple alkyl and aryl substituents, but also with such functional groups as nitro, cyano, hydroxy, amino, methoxycarbonyl, and sulfide, as well as 1*H*-indol-3-yl and halogen(s). Taking into account the easy accessibility of the starting materials and the exceptionally mild reaction conditions, the presented approach can be recommended for the synthesis of title 3-trifluoromethylated heterocycles, and nicely supplements previous reports on the synthesis of 1,2,4-triazin-6(1*H*)-ones exploiting amino acids and their derivatives as key building blocks [56,57,59,60,61,62,63,64].

## Data Availability

Raw electronic experimental NMR data (FIDs), as well as samples of all new compounds, are available from the authors.

## Supplementary Materials

The following supporting information can be downloaded at: https://www.mdpi.com/article/10.3390/ma16020856/s1: Copies of ^1^H and ^13^C NMR spectra of all new compounds, HPLC analyses, technical details, and results on biological activity screening. Ref. [65] cited in Supplementary Materials.
