# Supplementary material for: Trifluoromethylated 4,5-Dihydro-1,2,4-triazin-6(1H)-ones via (3+3)-Annulation of Nitrile Imines with α-Amino Esters
Source: Materials (Basel). 2023 Jan 16;16(2):856. doi: 10.3390/ma16020856 (PMC9864844; doi:10.3390/ma16020856)
Supplement: Supplementary file 1 [file materials-16-00856-s001.zip › materials-2140018-supplementary.pdf]

Supporting Information

for

Trifluoromethylated 4,5-dihydro-1,2,4-triazin-6(1*H*)-ones *via* (3+3)-annulation of nitrile imines with α-amino esters

Anna Kowalczyk,[a,b] Kamil Świątek,[a,b] Małgorzata Celeda,[a] Greta Utecht-Jarzyńska,[a]

Agata Jaskulska,[c] Katarzyna Gach-Janczak,[d] Marcin Jasiński[a]*

[a] *Faculty of Chemistry, University of Lodz, Tamka 12, 91403 Łódź, Poland*

[b] *Doctoral School of Exact and Natural Sciences, University of Lodz, Banacha 12/16, 90237 Łódź, Poland*

[c] *Institute of Organic Chemistry, Faculty of Chemistry, Lodz University of Technology, Żeromskiego 116, 90 924, Łódź, Poland*

[d] *Department of Biomolecular Chemistry, Medical University of Lodz, Mazowiecka 6/8, 92215 Łódź, Poland*

***** *Corresponding author: Marcin Jasiński – University of Lodz, Faculty of Chemistry, Łódź, Poland*

https://orcid.org/0000-0002-8789-9690; phone: +48 43 635 5766; e-mail: mjasinski@uni.lodz.pl

Content

Copies of ^{1}H and ^{13}C NMR spectra of new compounds S2

HPLC analyses S36

Cytotoxicity tests S37

References S39

Copies of ^{1}H and ^{13}C NMR spectra of new compounds

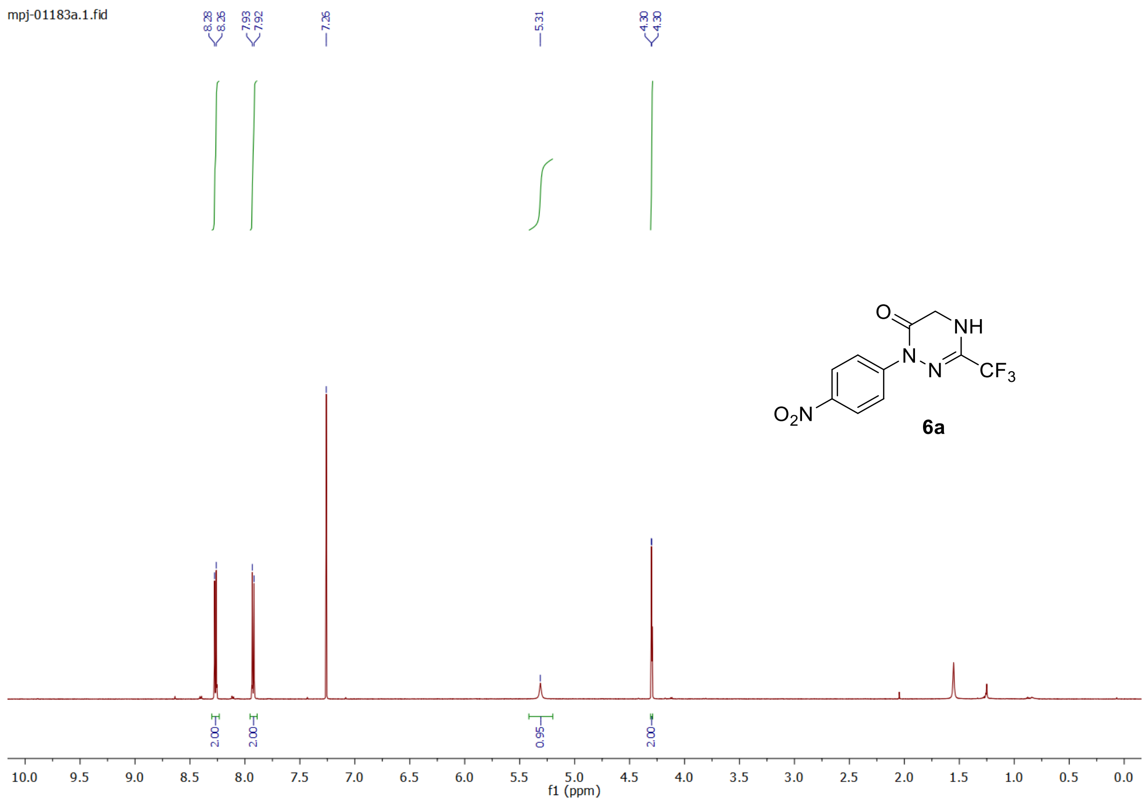

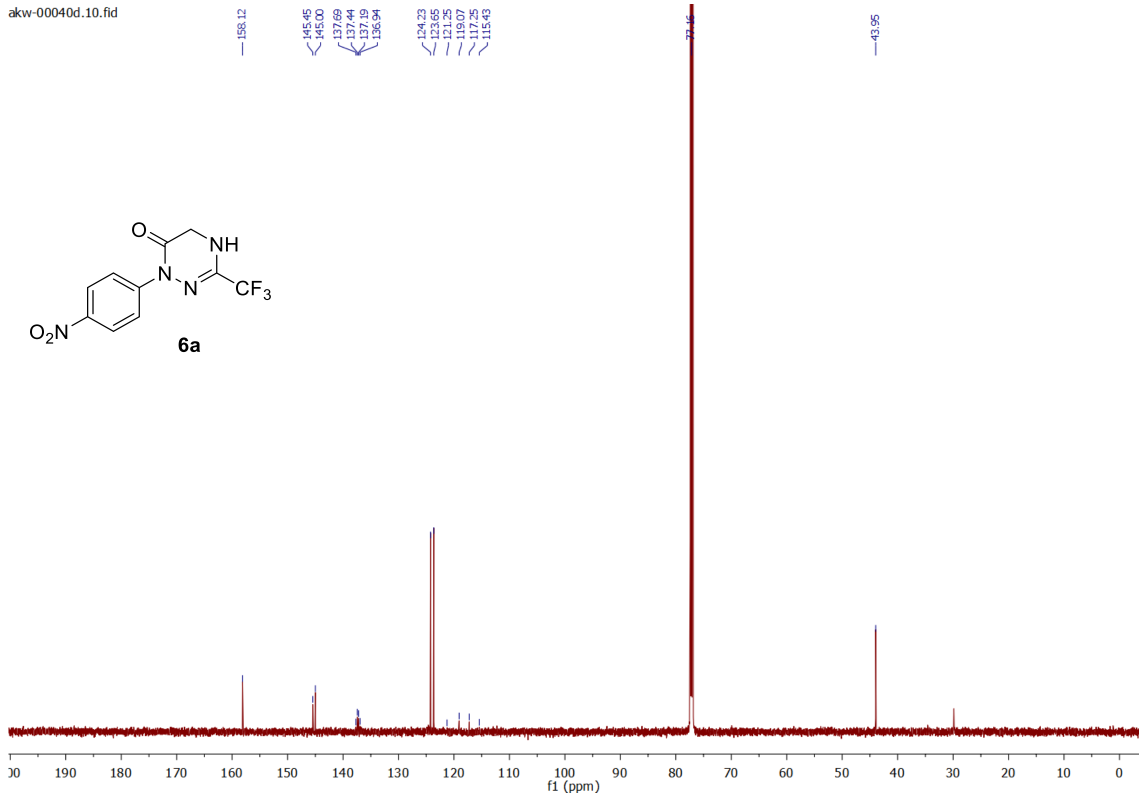

Figure S1. ^{1}H NMR (600 MHz, $CDCl_3$) and ^{13}C NMR (151 MHz, $CDCl_3$) spectra for compound **6a**.

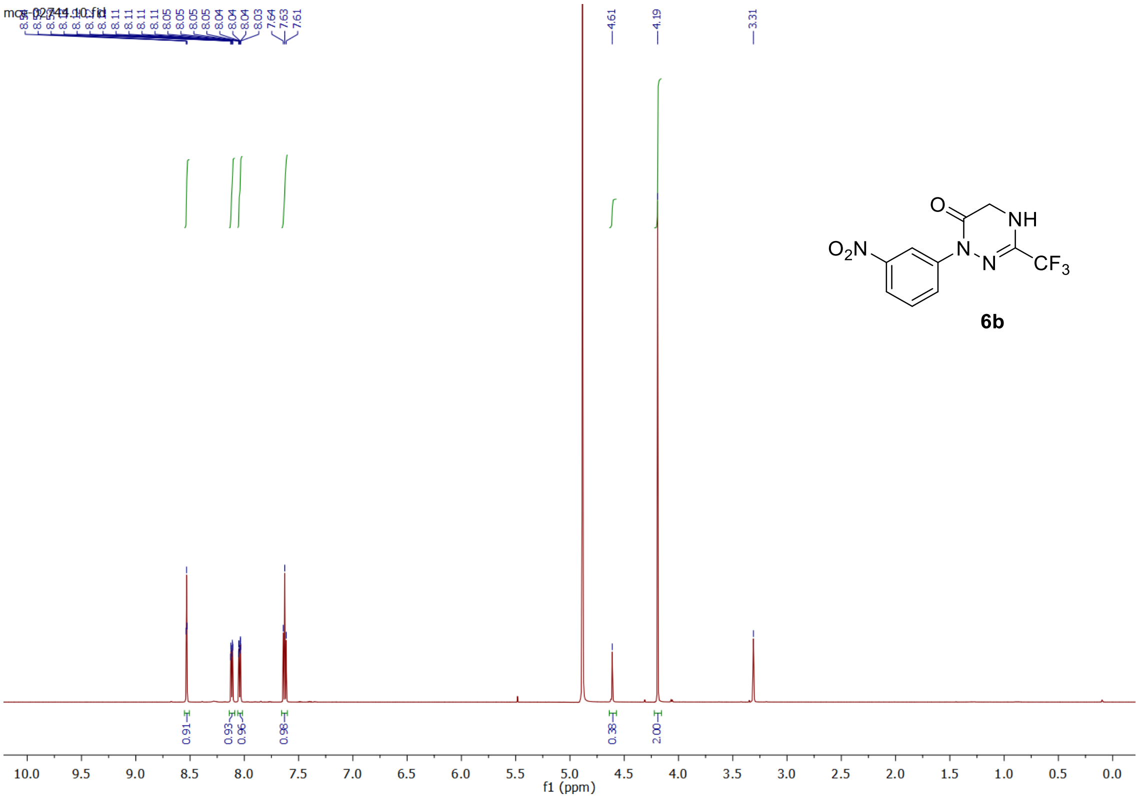

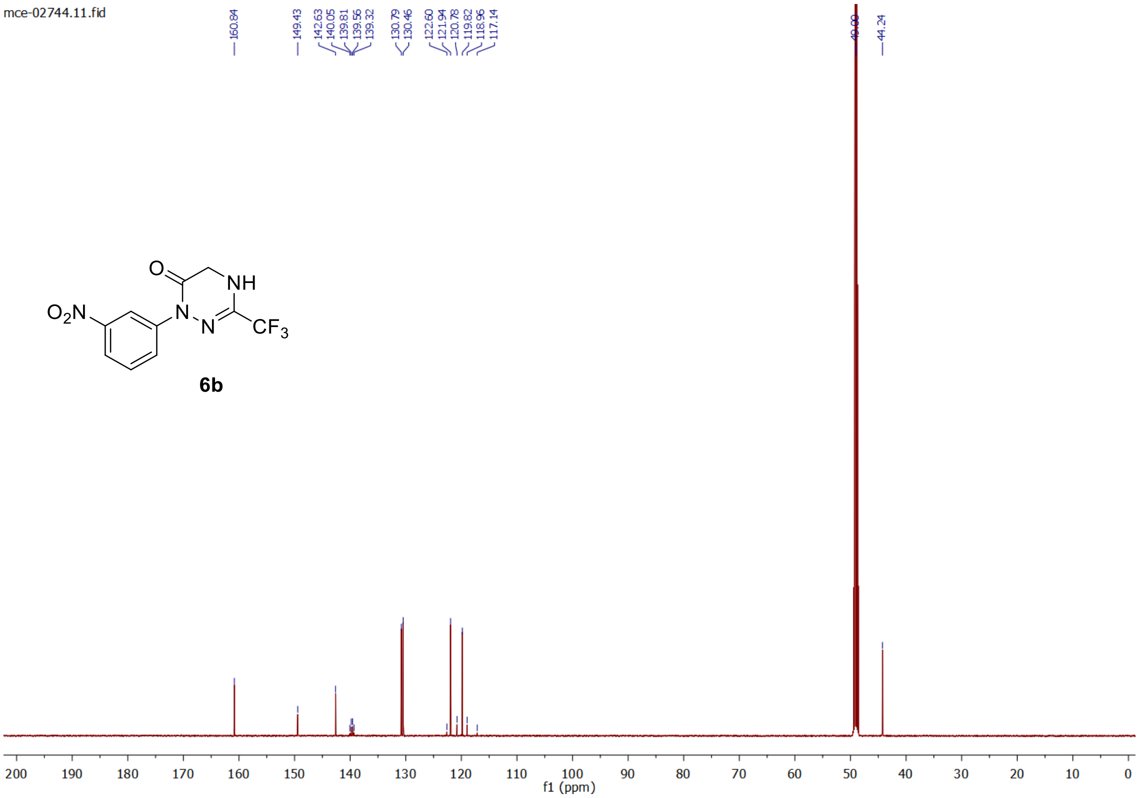

Figure S2. ^{1}H NMR (600 MHz, CD_3OD) and ^{13}C NMR (151 MHz, CD_3OD) spectra for compound **6b**.

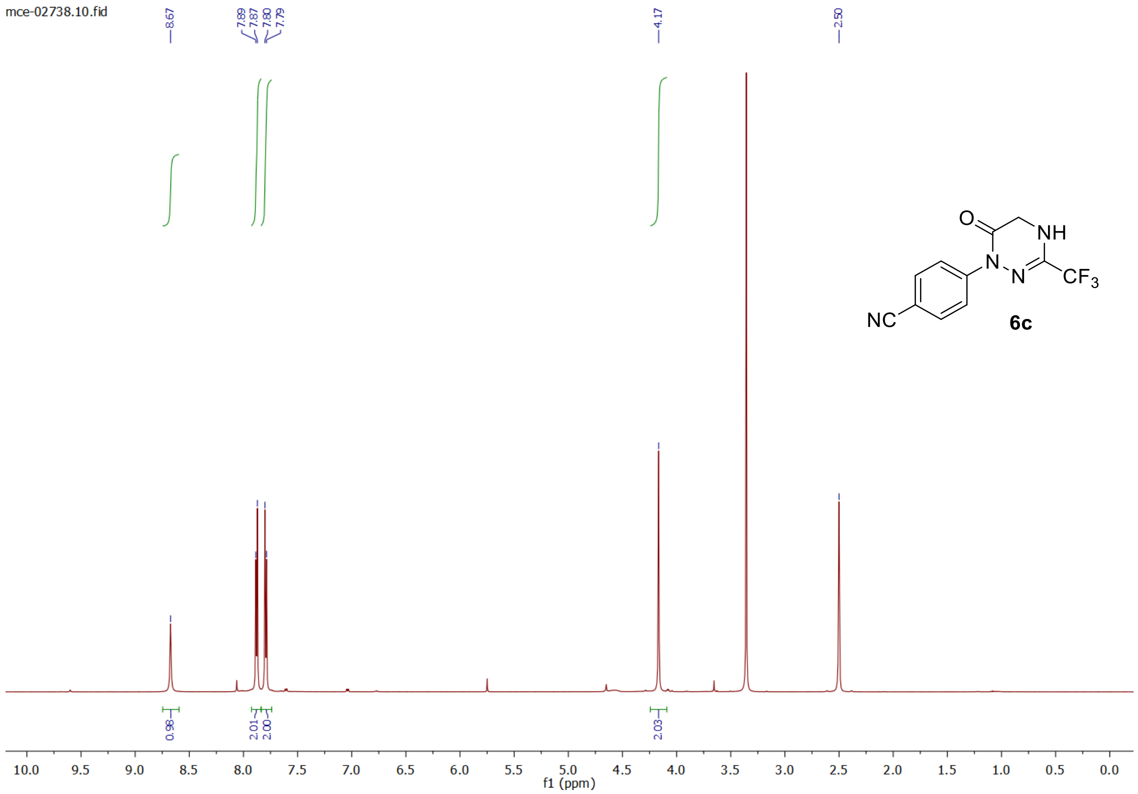

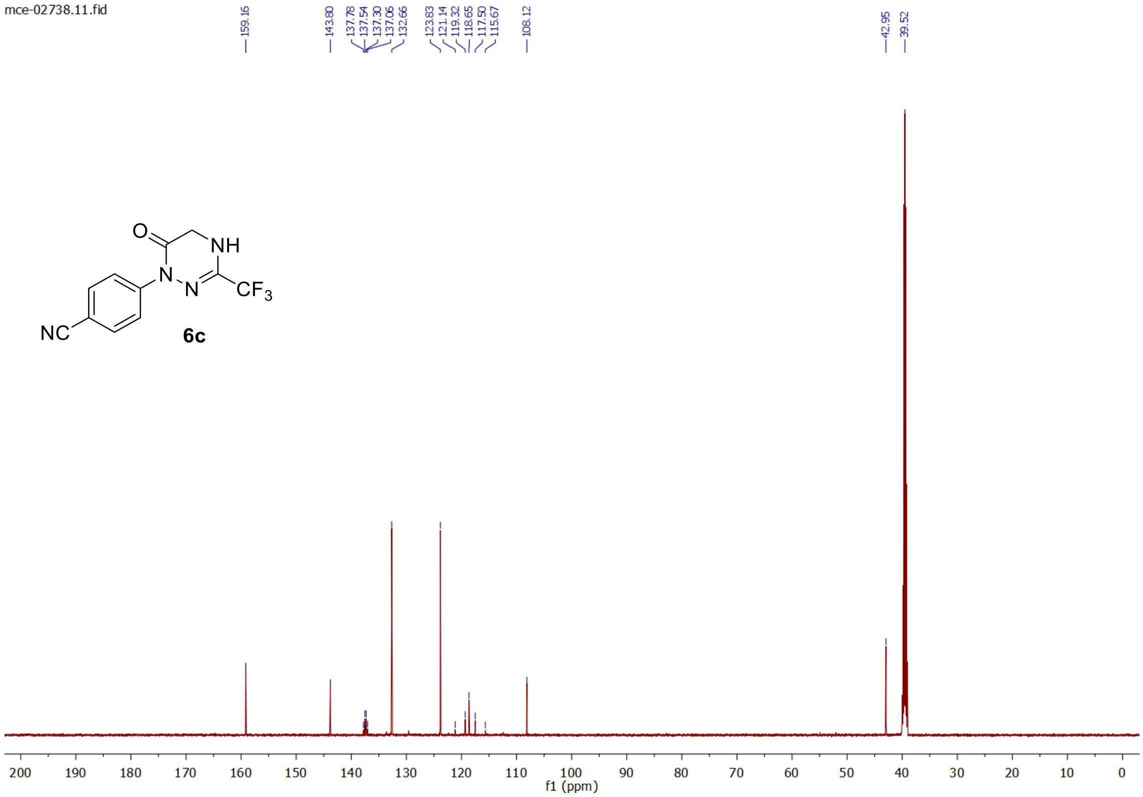

Figure S3. ^{1}H NMR (600 MHz, DMSO-d_6) and ^{13}C NMR (151 MHz, DMSO-d_6) spectra for compound **6c**.

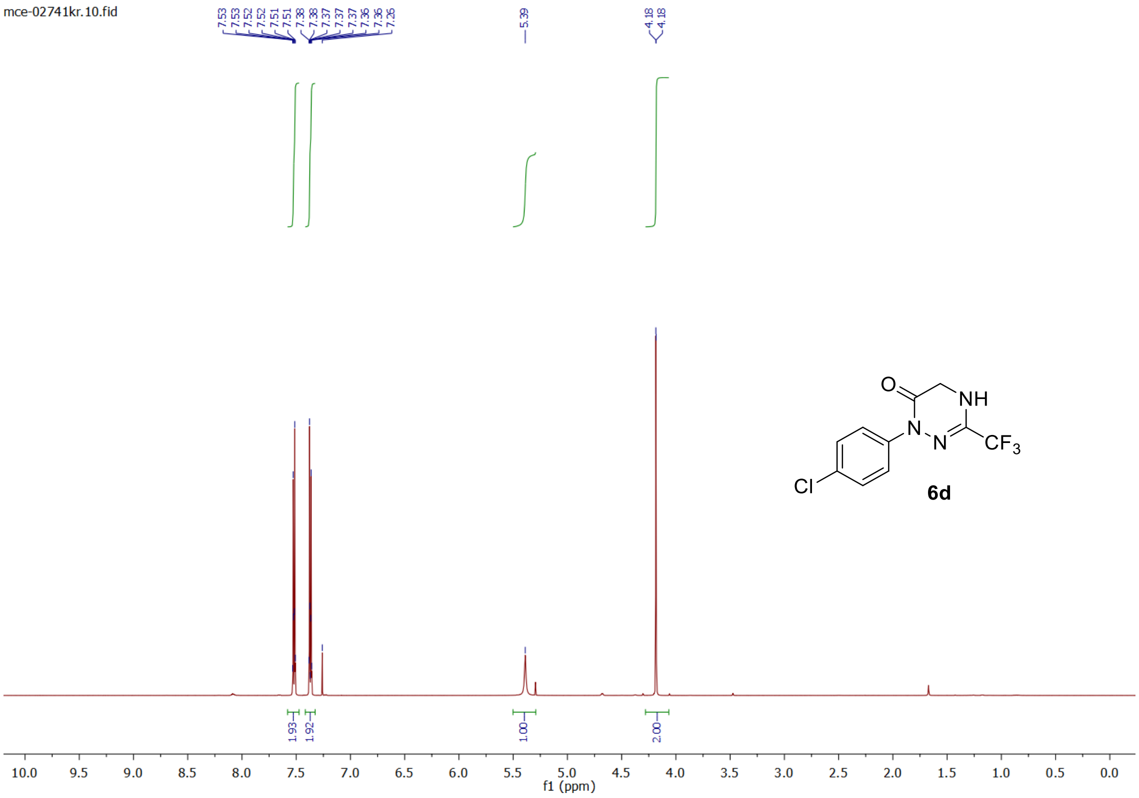

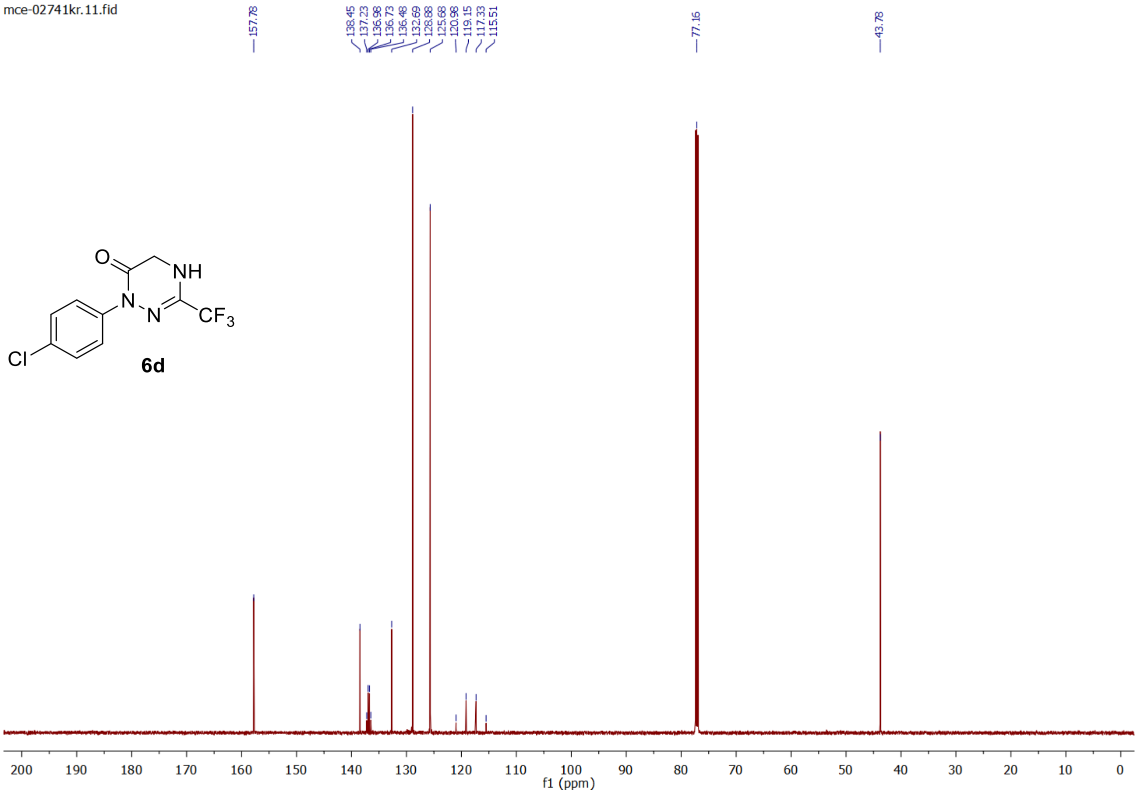

Figure S4. ^{1}H NMR (600 MHz, $CDCl_3$) and ^{13}C NMR (151 MHz, $CDCl_3$) spectra for compound **6d**.

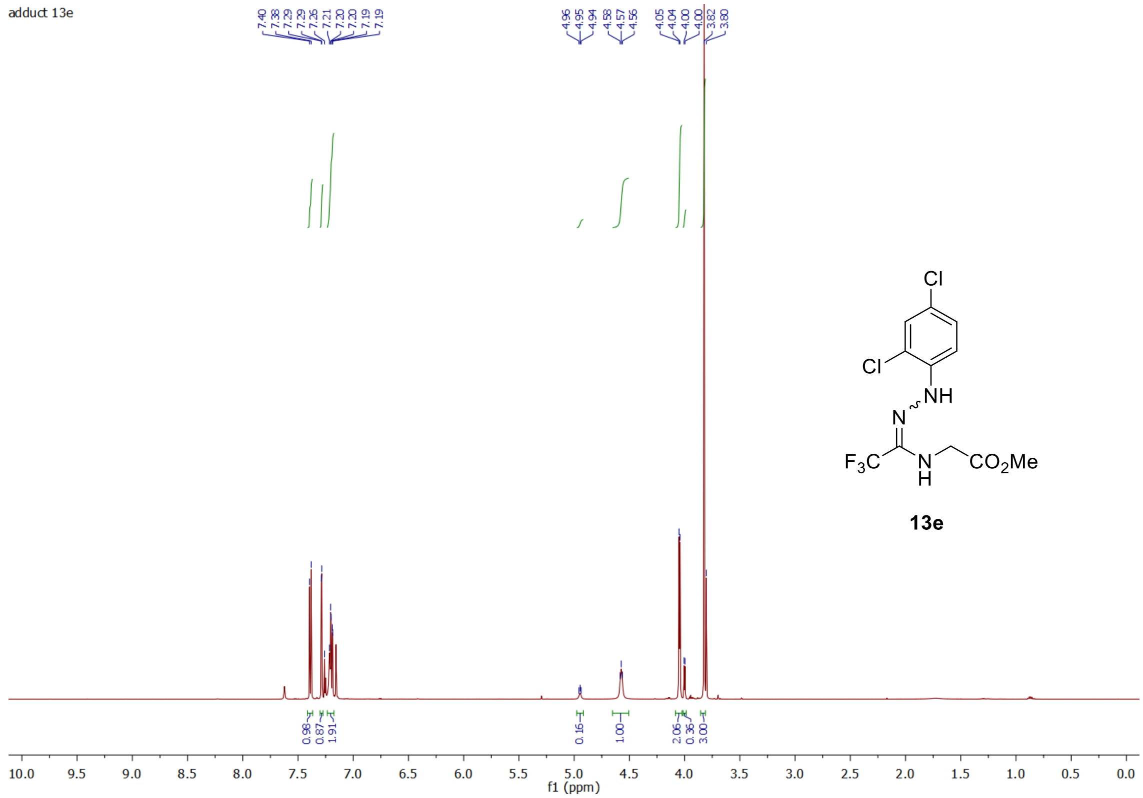

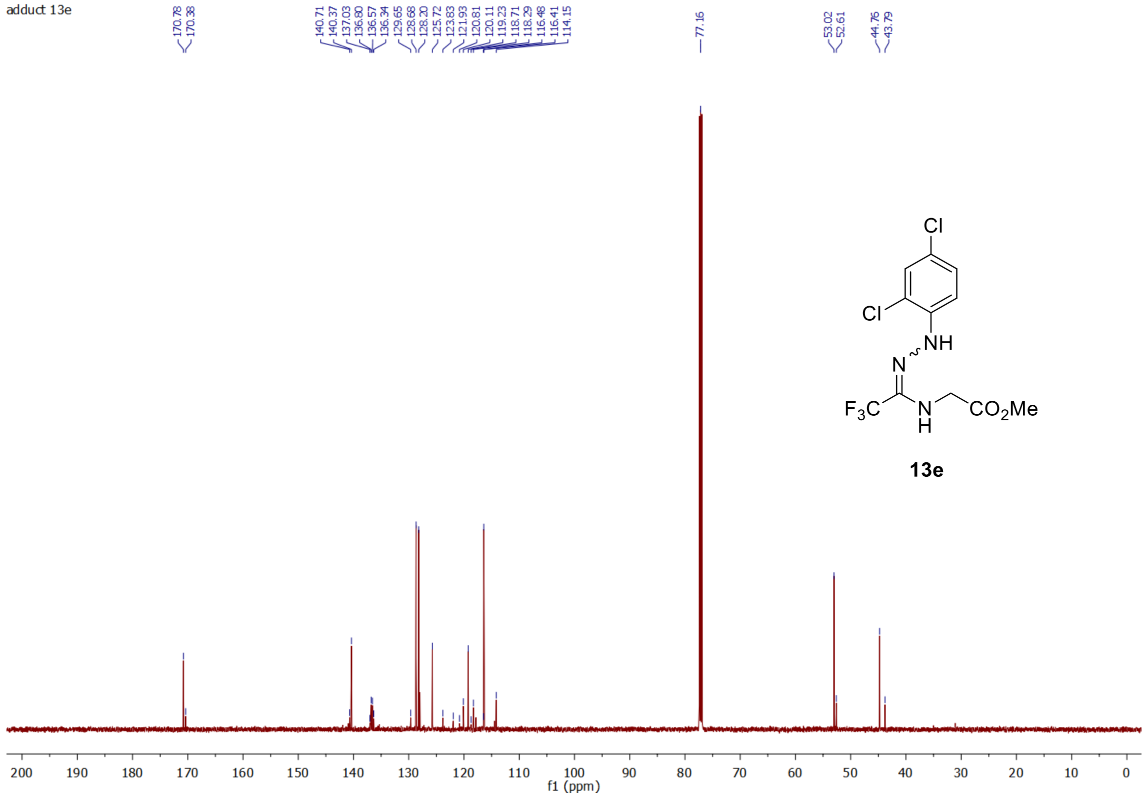

Figure S5. ^{1}H NMR (600 MHz, $CDCl_3$) and ^{13}C NMR (151 MHz, $CDCl_3$) spectra for compound **13e**.

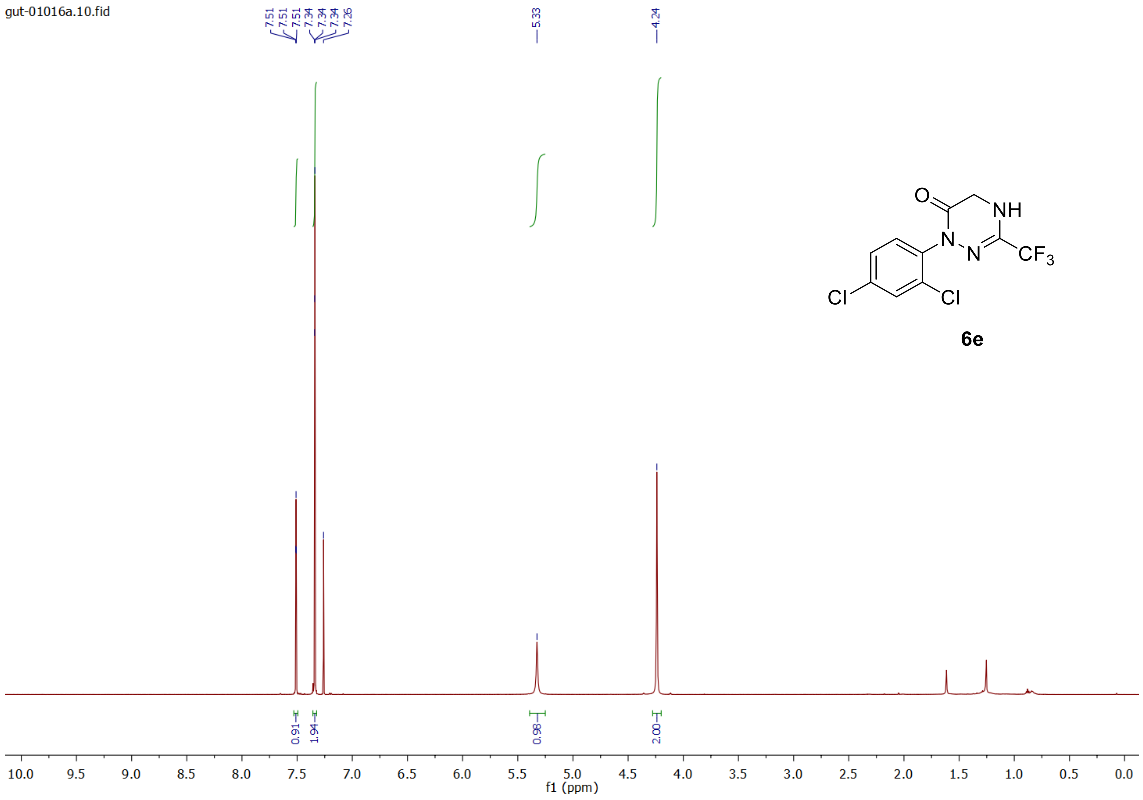

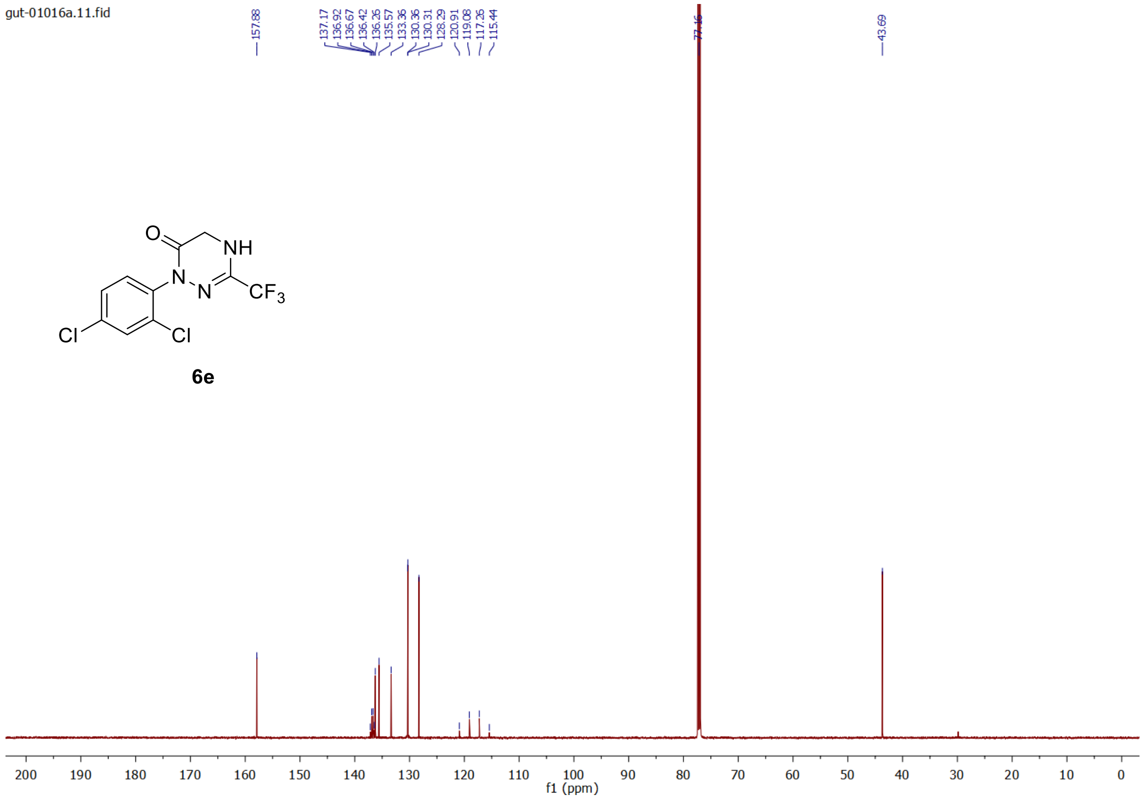

Figure S6. ^{1}H NMR (600 MHz, $CDCl_3$) and ^{13}C NMR (151 MHz, $CDCl_3$) spectra for compound **6e**.

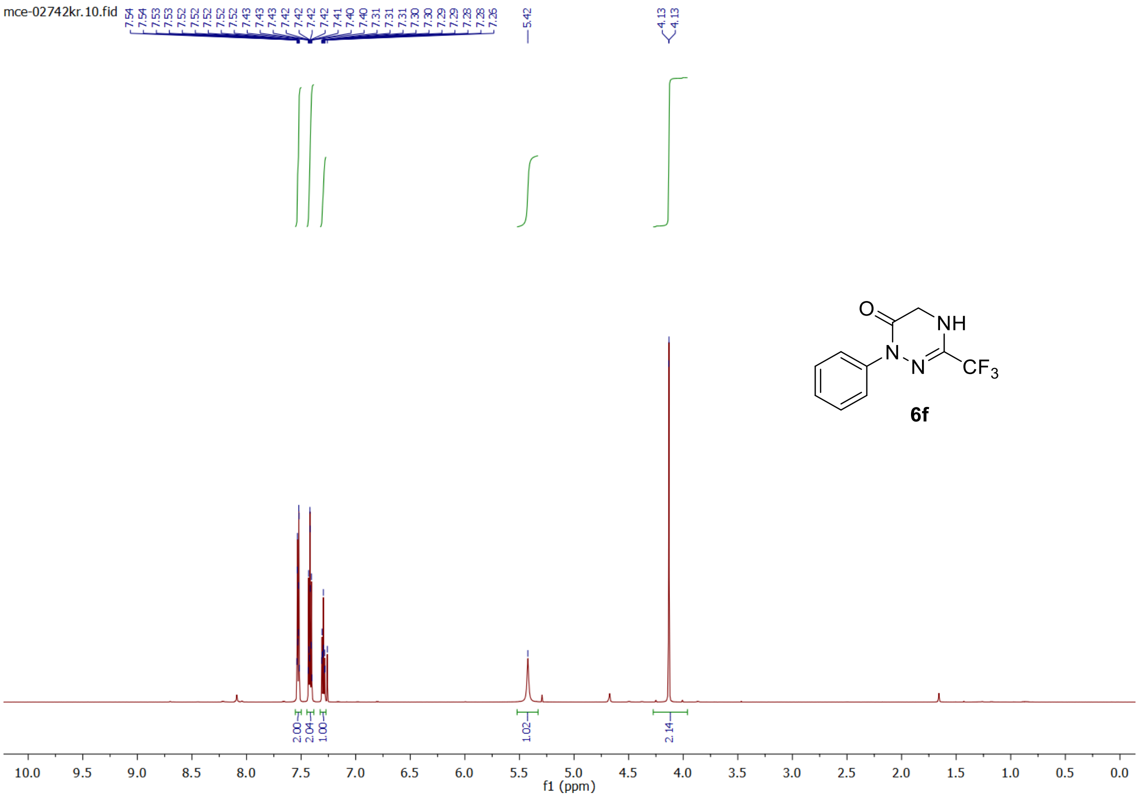

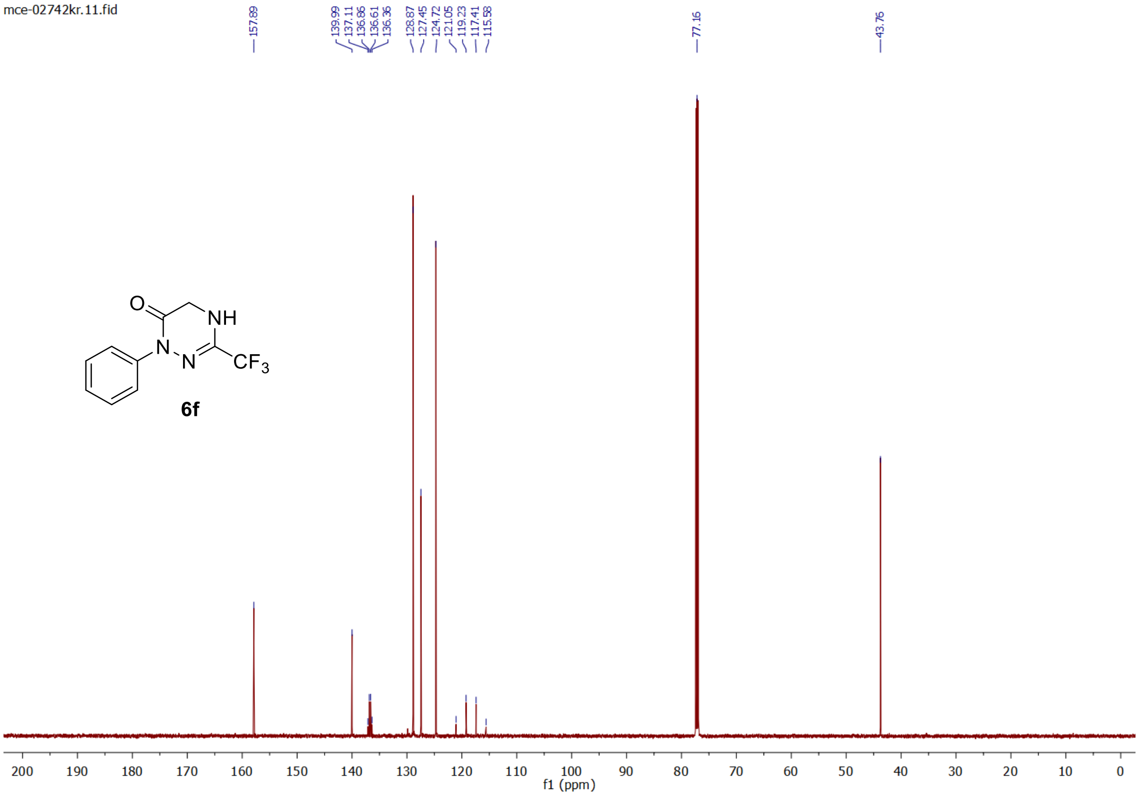

Figure S7. ^{1}H NMR (600 MHz, $CDCl_3$) and ^{13}C NMR (151 MHz, $CDCl_3$) spectra for compound **6f**.

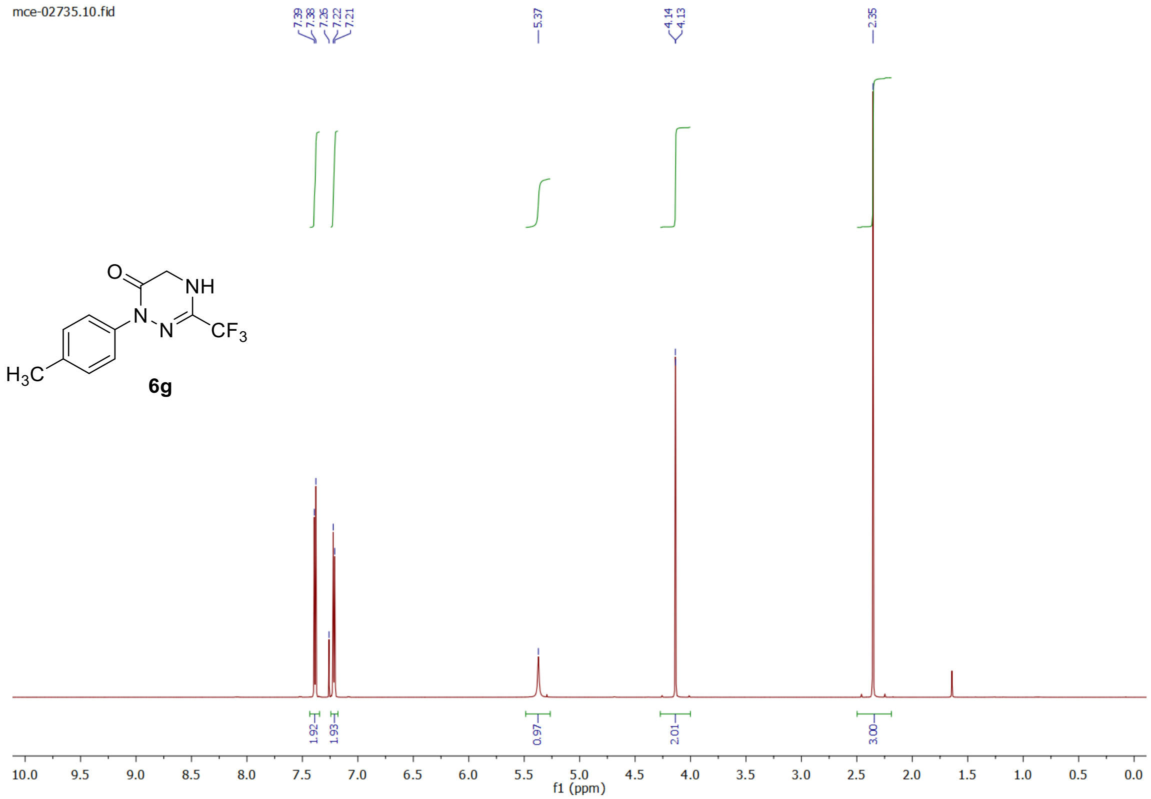

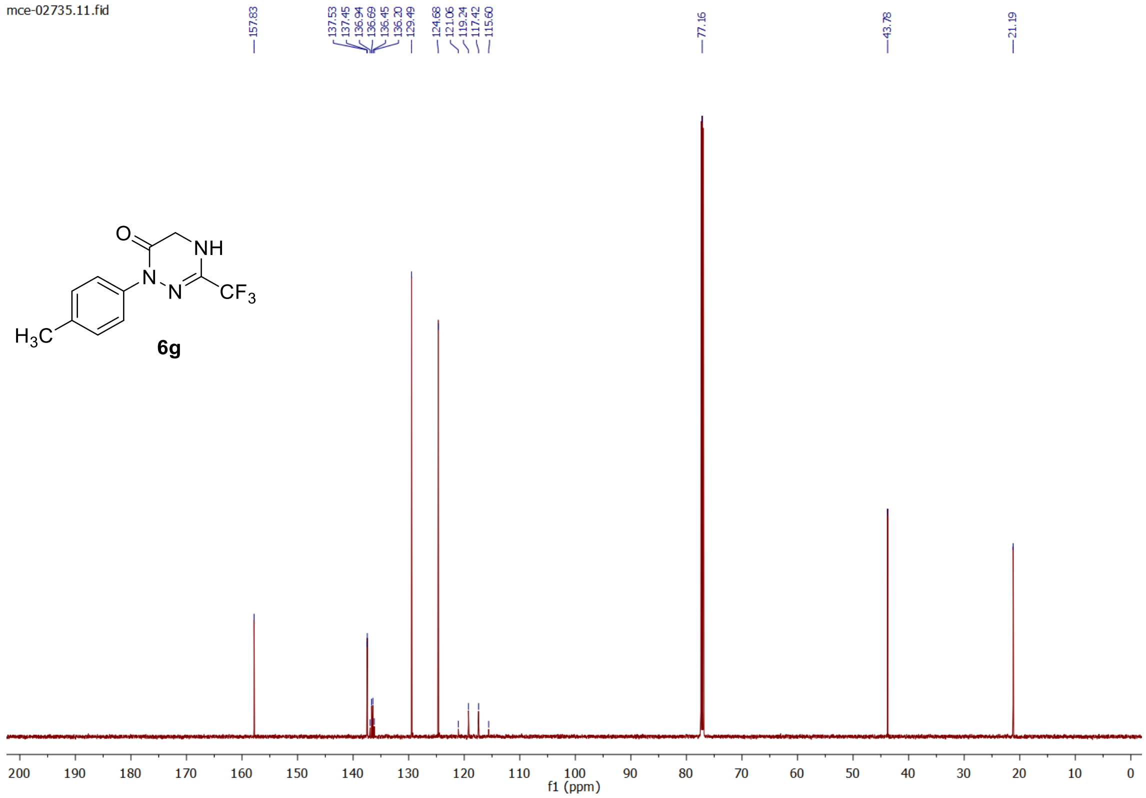

Figure S8. ^{1}H NMR (600 MHz, $CDCl_3$) and ^{13}C NMR (151 MHz, $CDCl_3$) spectra for compound **6g**.

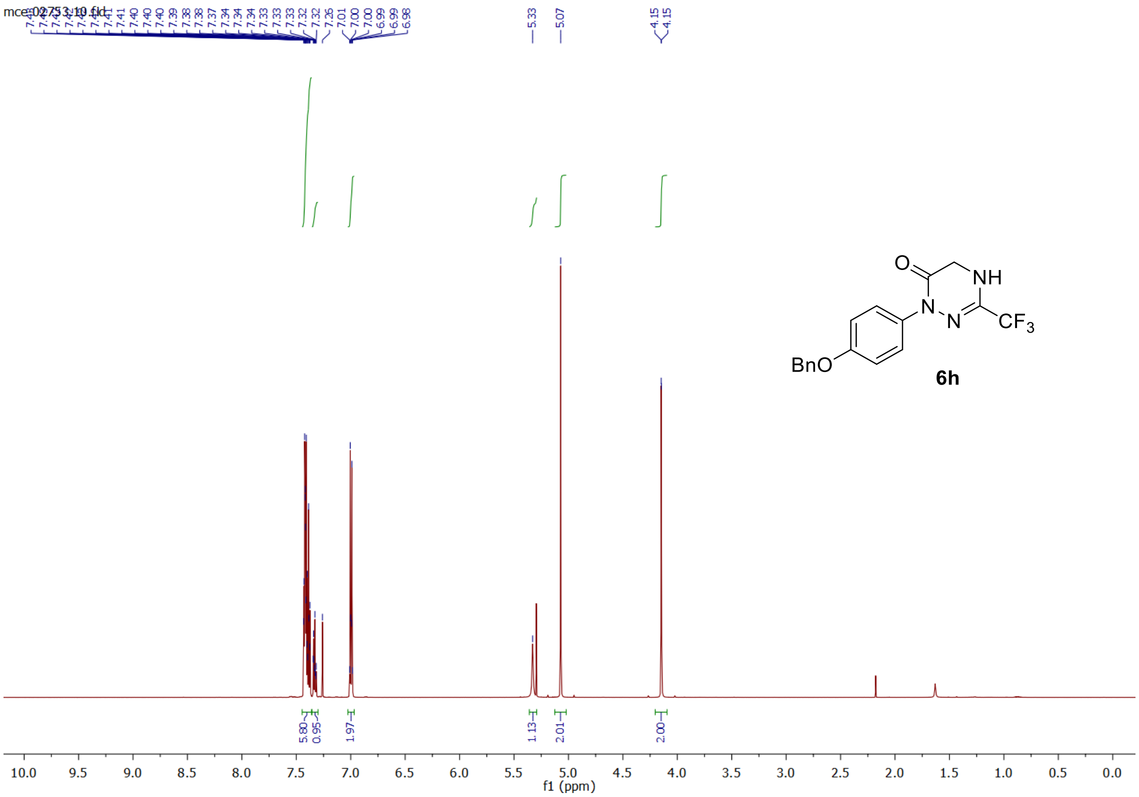

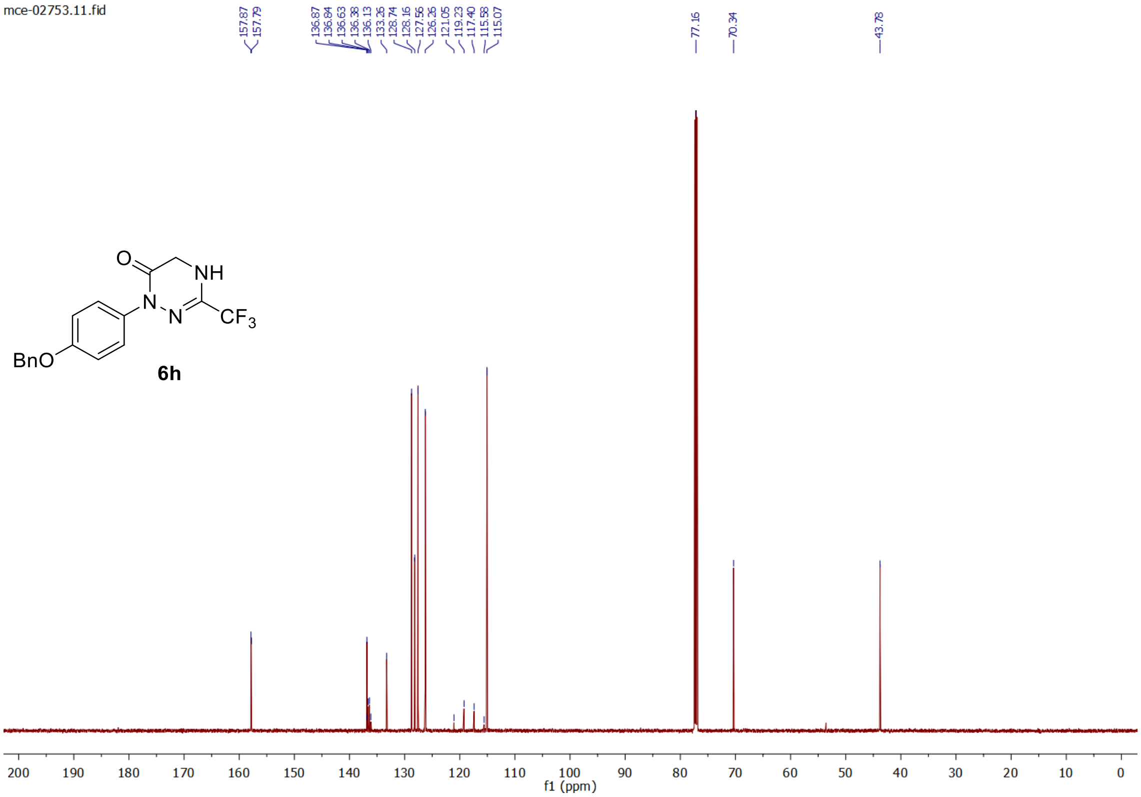

Figure S9. ^{1}H NMR (600 MHz, $CDCl_3$) and ^{13}C NMR (151 MHz, $CDCl_3$) spectra for compound **6h**.

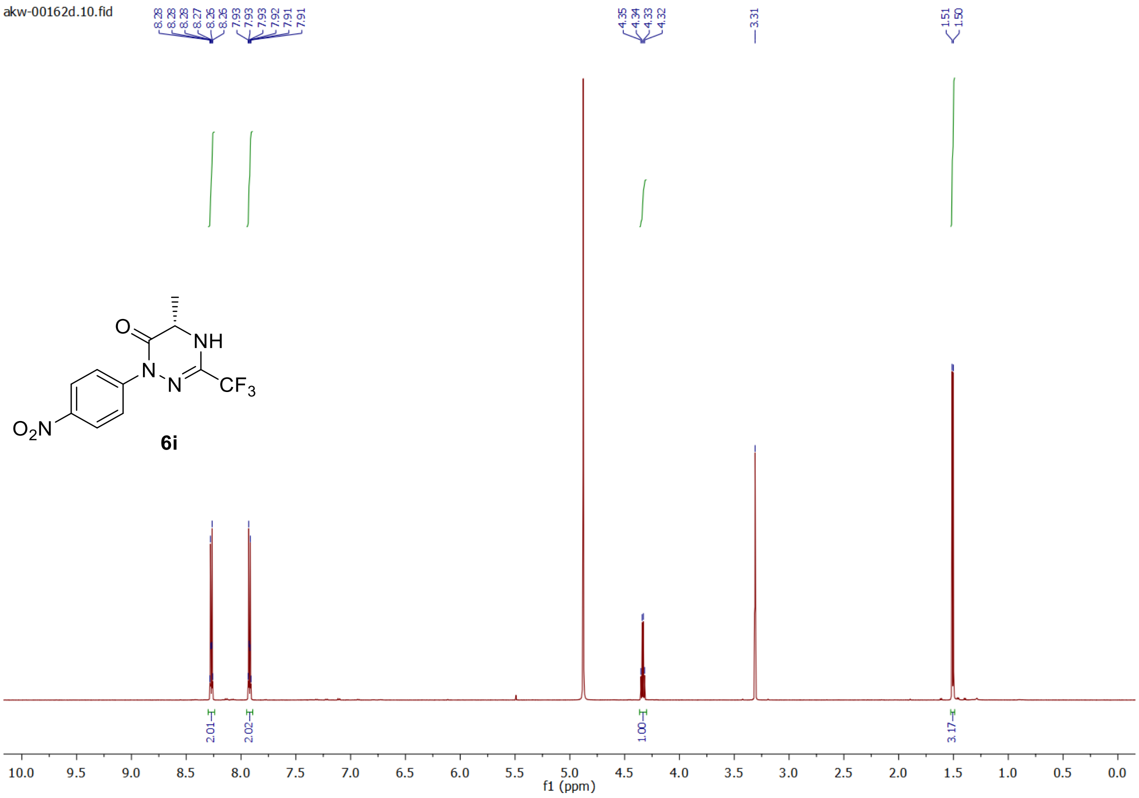

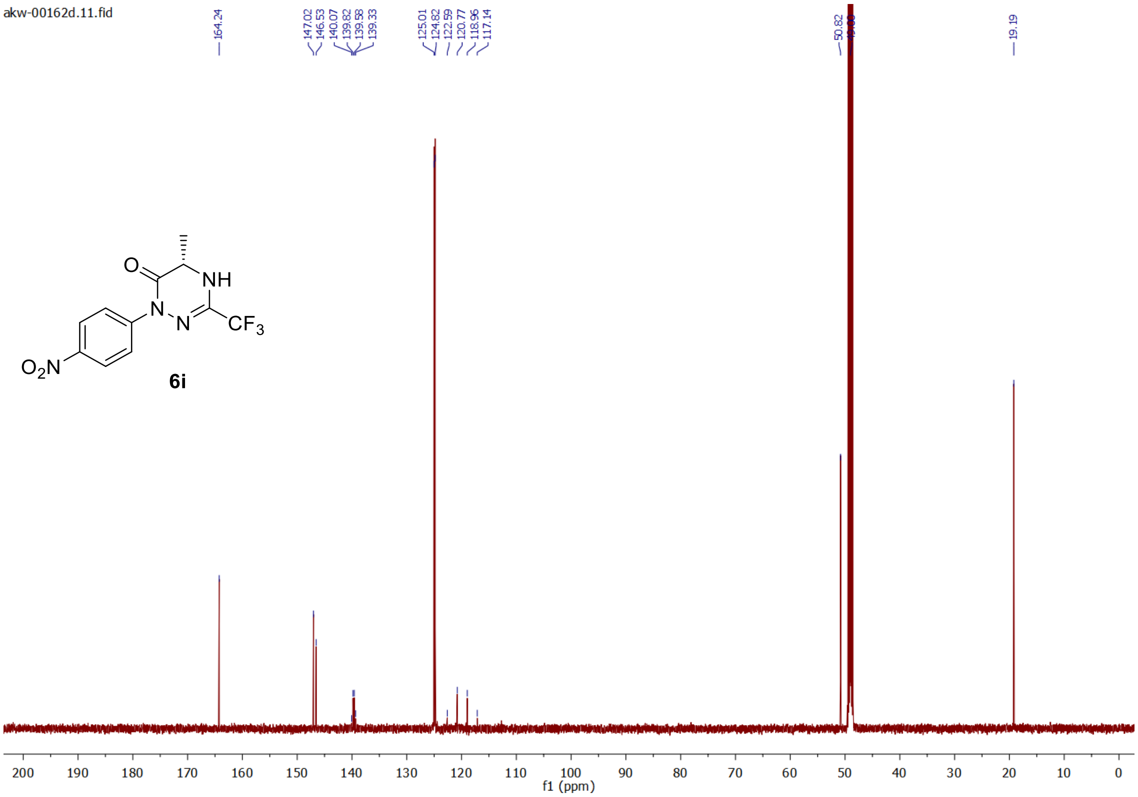

Figure S10. ^{1}H NMR (600 MHz, CD_3OD) and ^{13}C NMR (151 MHz, CD_3OD) spectra for compound **6i**.

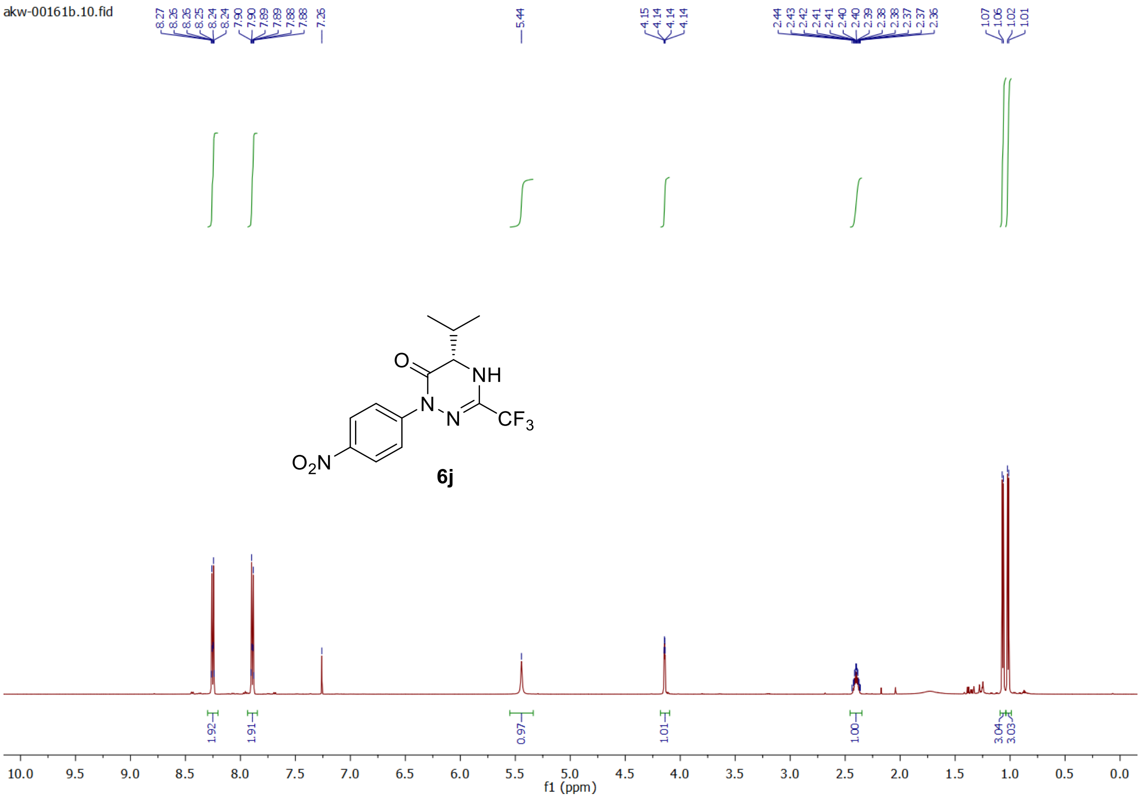

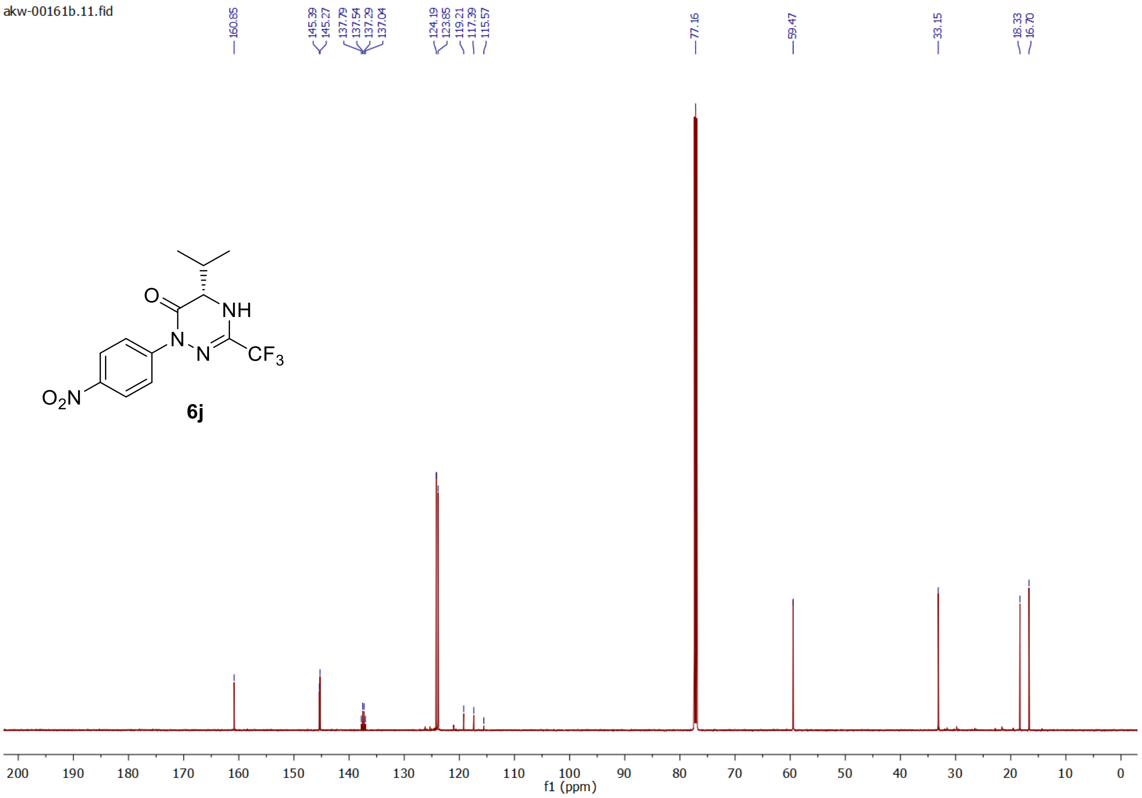

Figure S11. ^{1}H NMR (600 MHz, $CDCl_3$) and ^{13}C NMR (151 MHz, $CDCl_3$) spectra for compound **6j**.

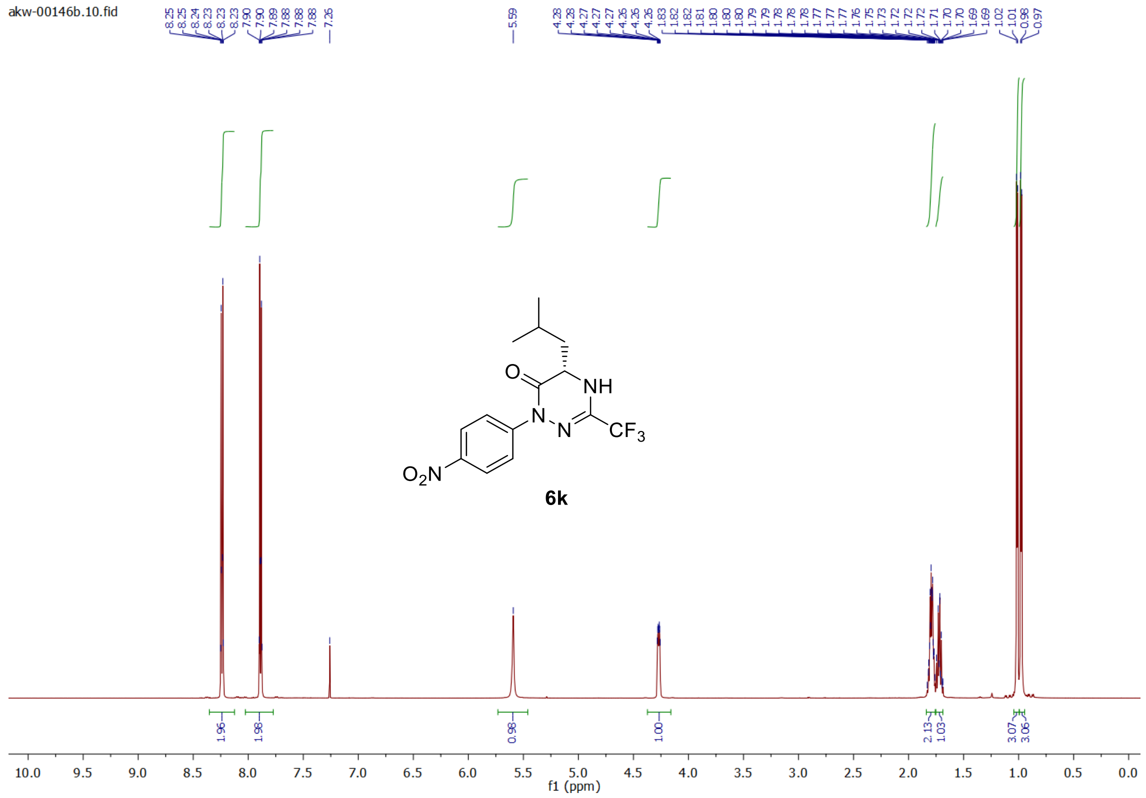

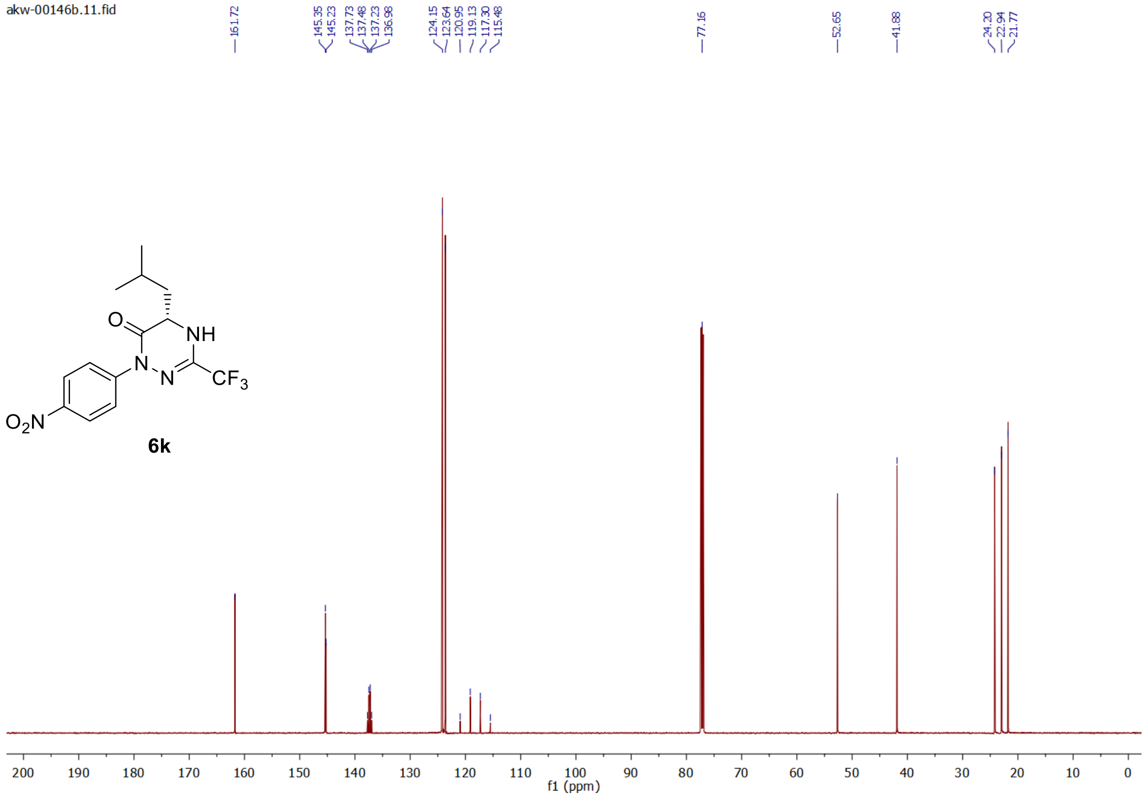

Figure S12. ^{1}H NMR (600 MHz, $CDCl_3$) and ^{13}C NMR (151 MHz, $CDCl_3$) spectra for compound **6k**.

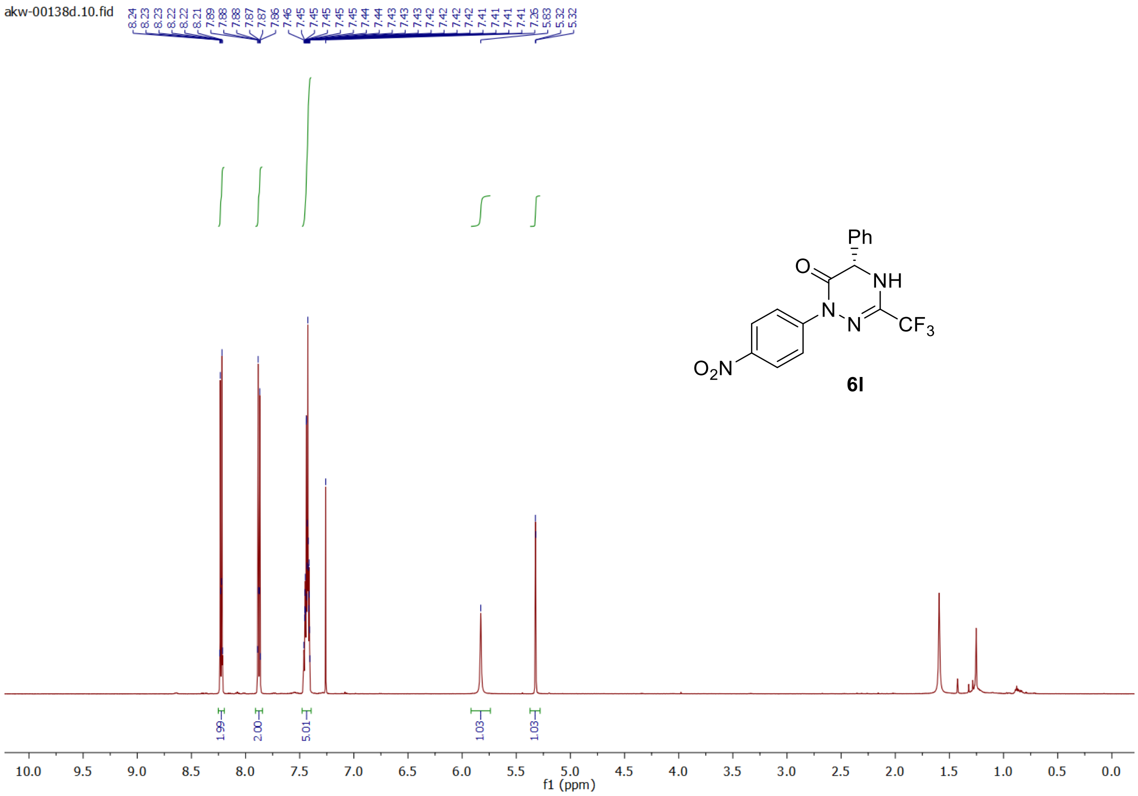

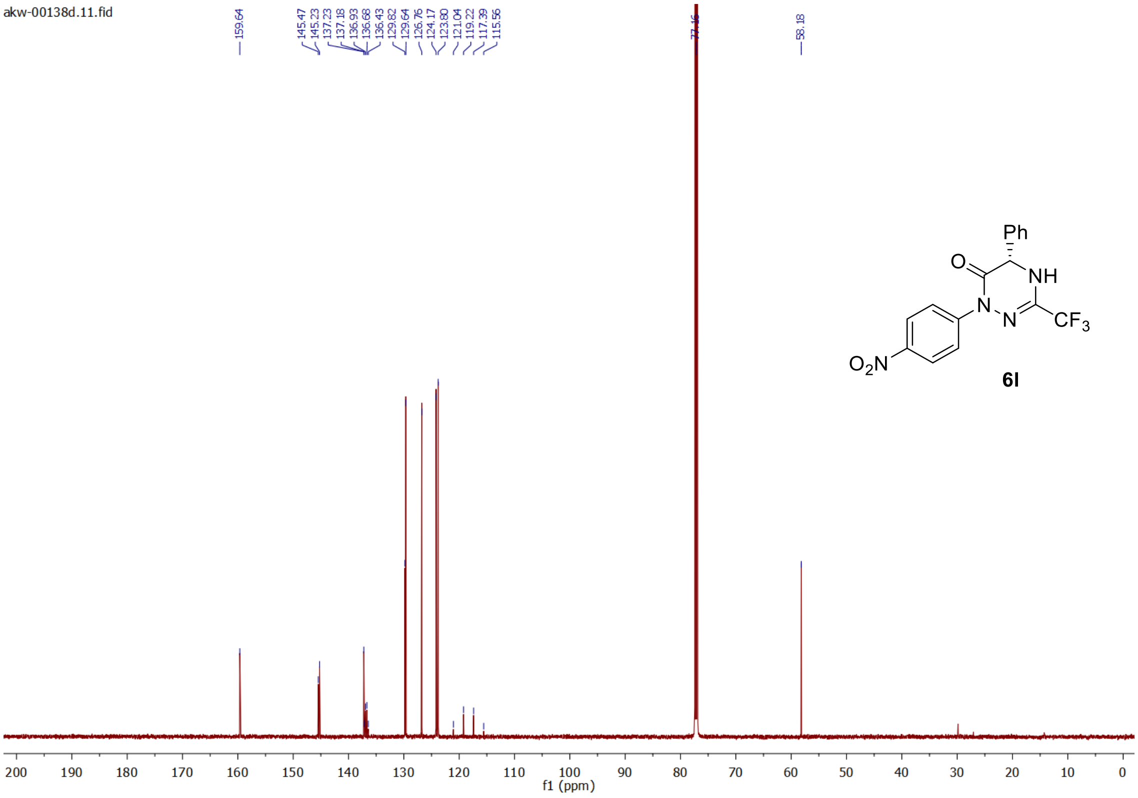

Figure S13. ^{1}H NMR (600 MHz, $CDCl_3$) and ^{13}C NMR (151 MHz, $CDCl_3$) spectra for compound **6l**.

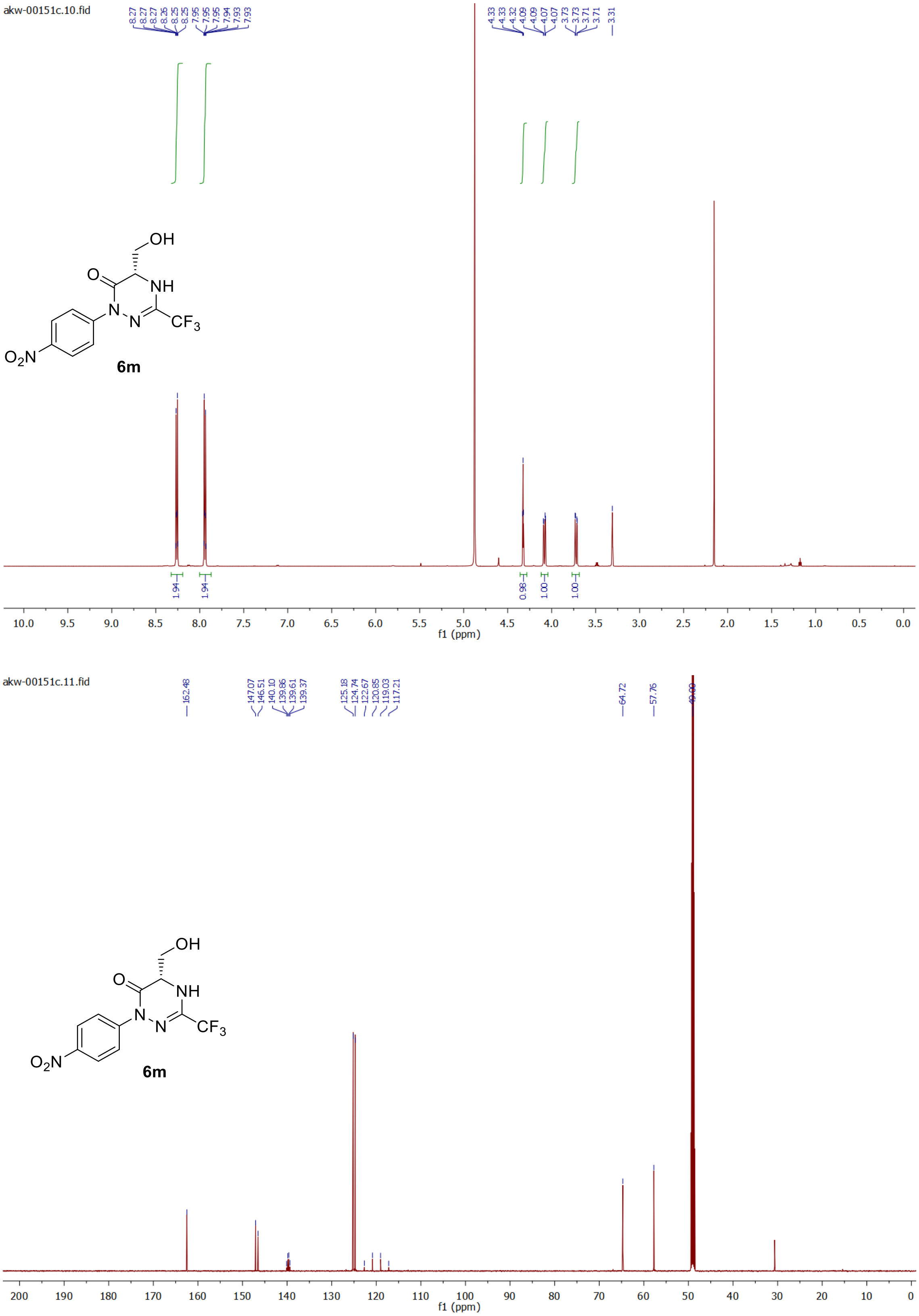

Figure S14. ^{1}H NMR (600 MHz, CD_3OD) and ^{13}C NMR (151 MHz, CD_3OD) spectra for compound **6m**.

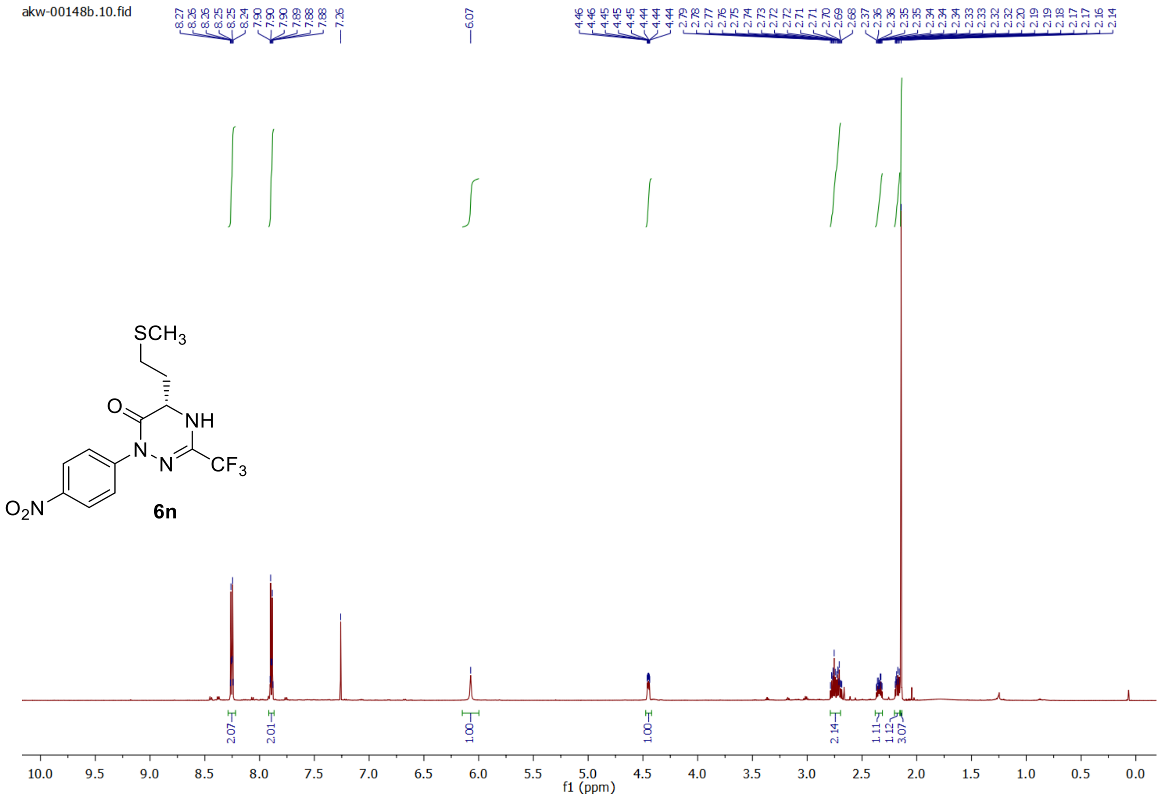

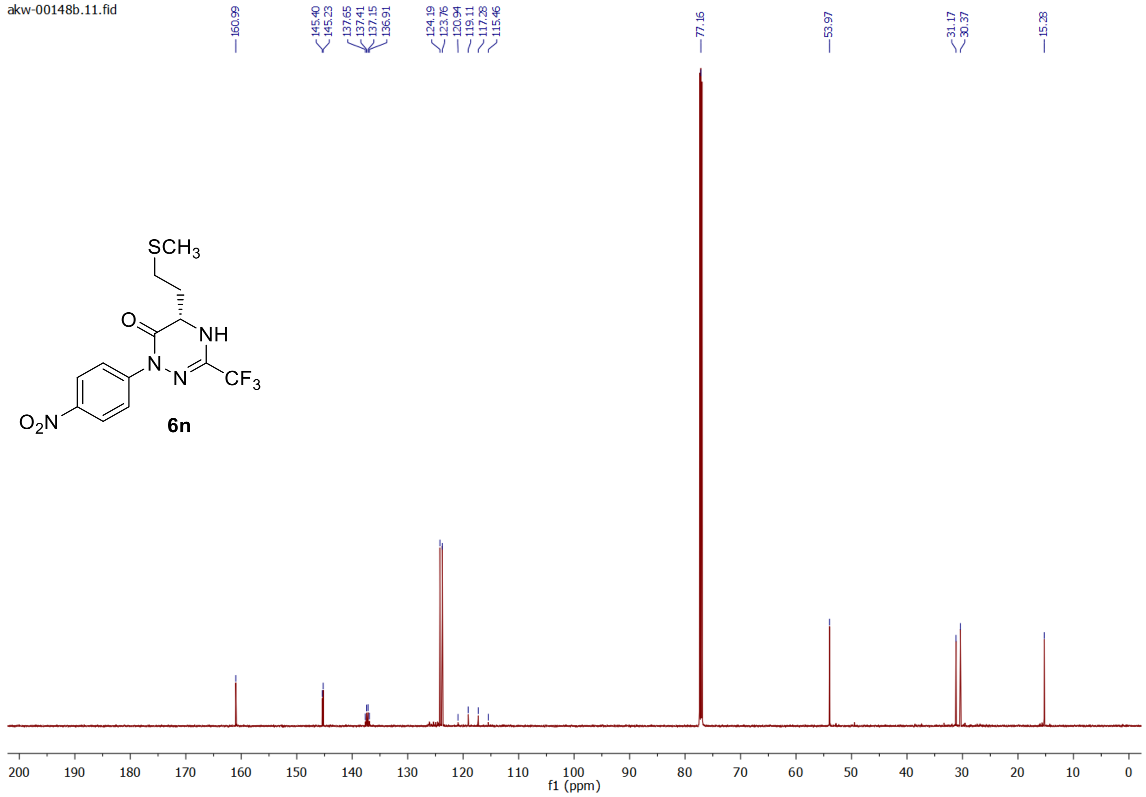

Figure S15. ^1H NMR (600 MHz, $CDCl_3$) and ^{13}C NMR (151 MHz, $CDCl_3$) spectra for compound **6n**.

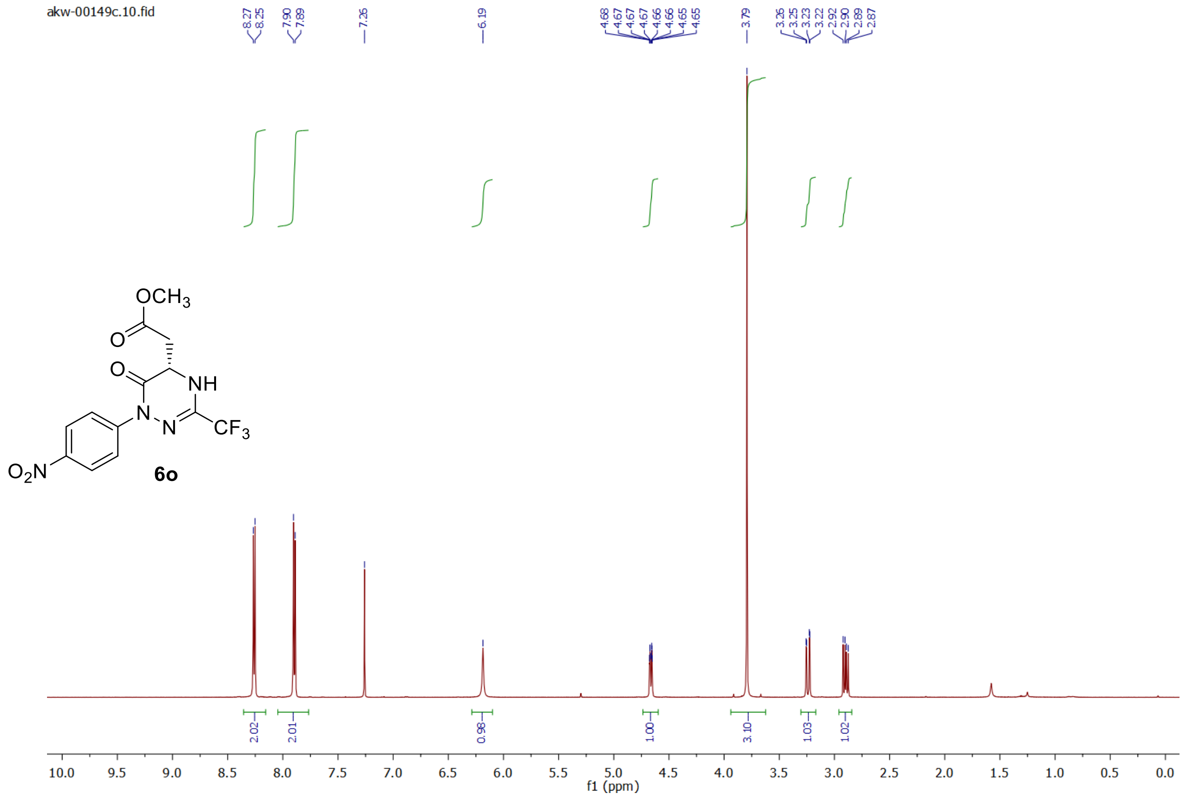

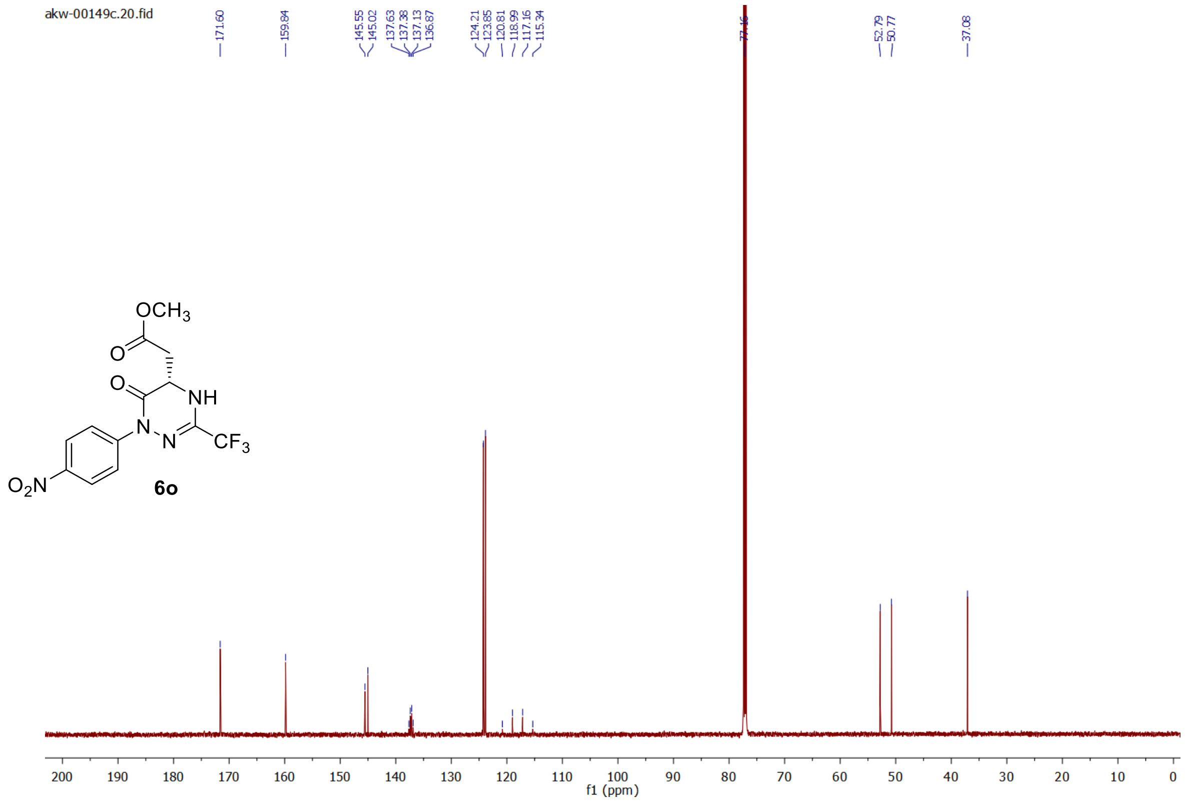

Figure S16. ^{1}H NMR (600 MHz, $CDCl_3$) and ^{13}C NMR (151 MHz, $CDCl_3$) spectra for compound **6o**.

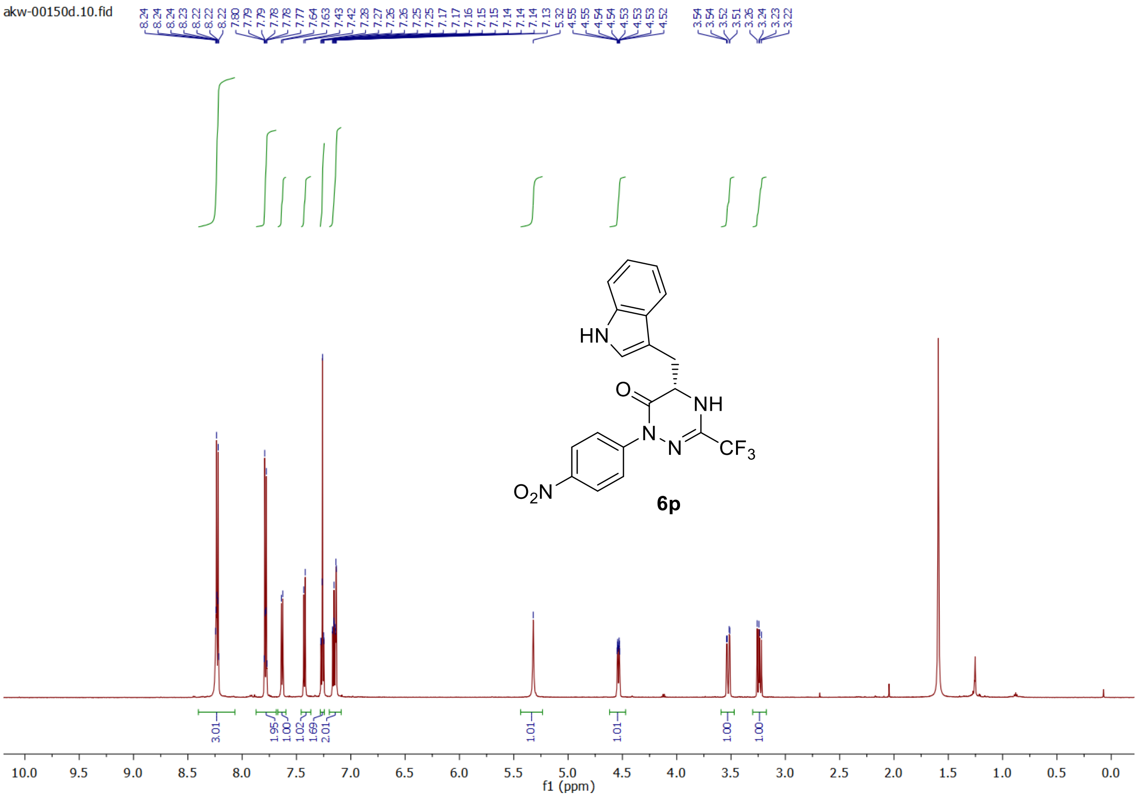

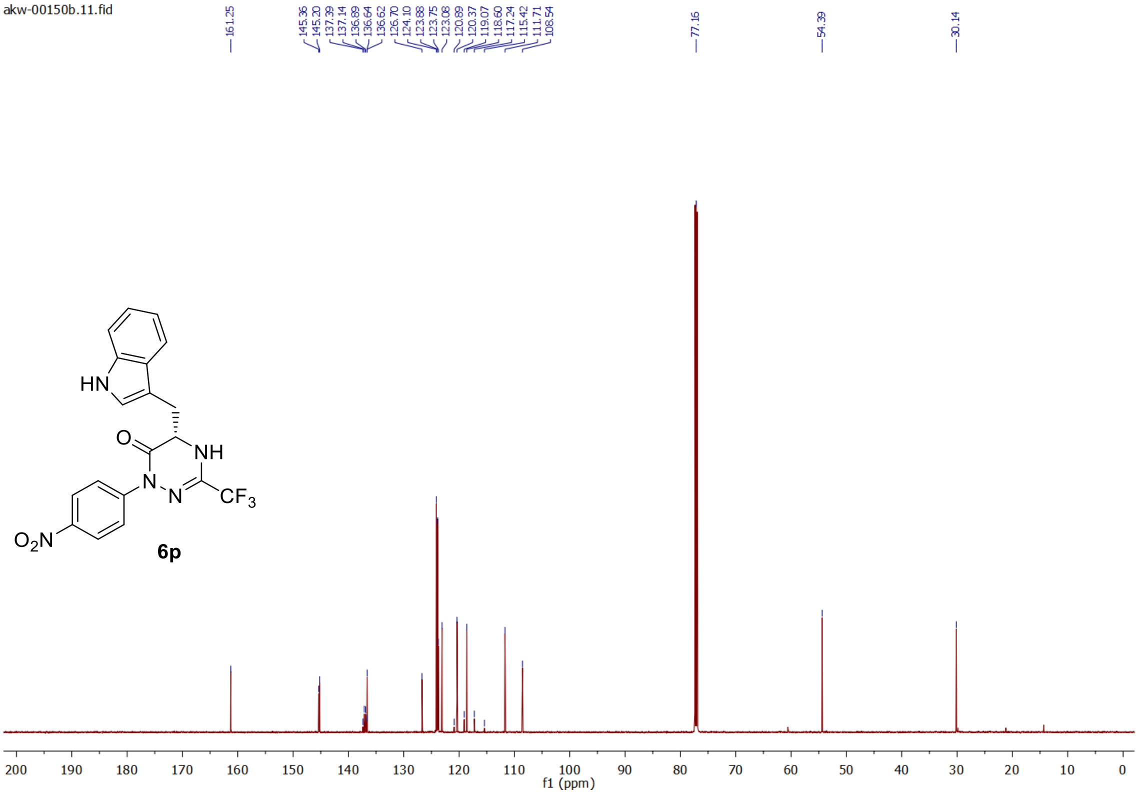

Figure S17. ^{1}H NMR (600 MHz, CDCl$_3$) and ^{13}C NMR (151 MHz, CDCl$_3$) spectra for compound **6p**.

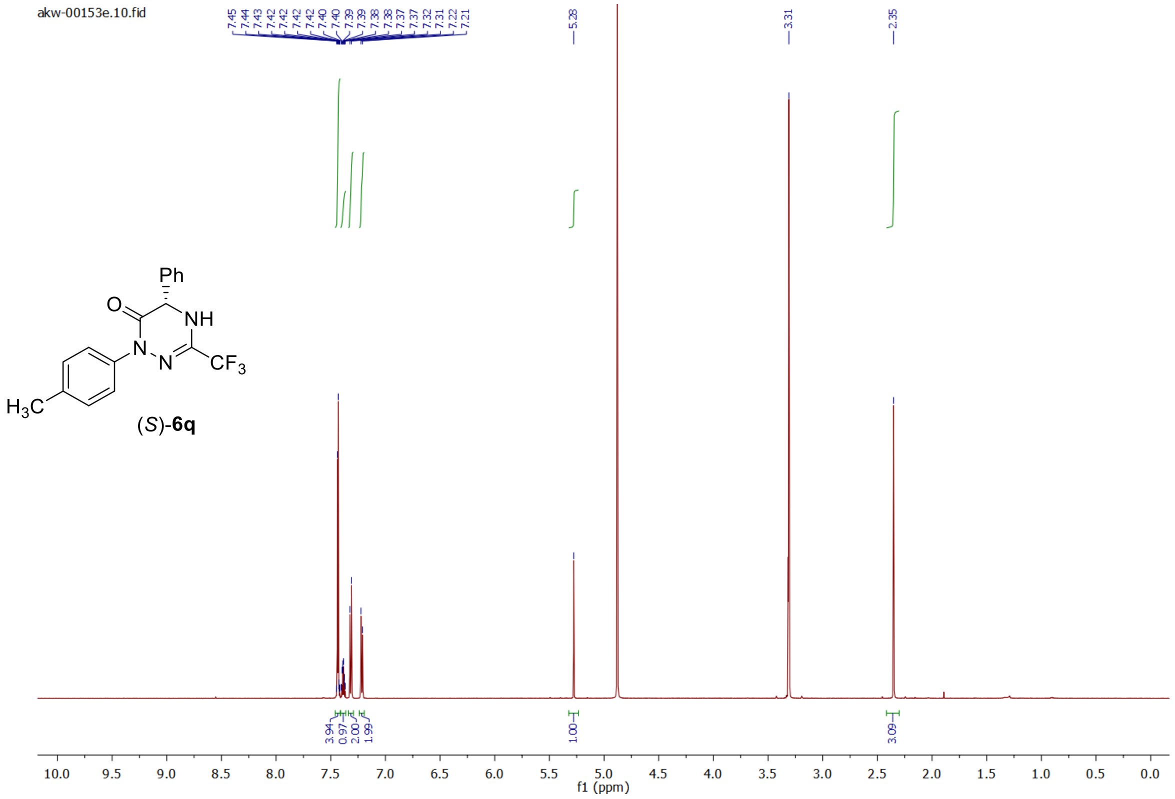

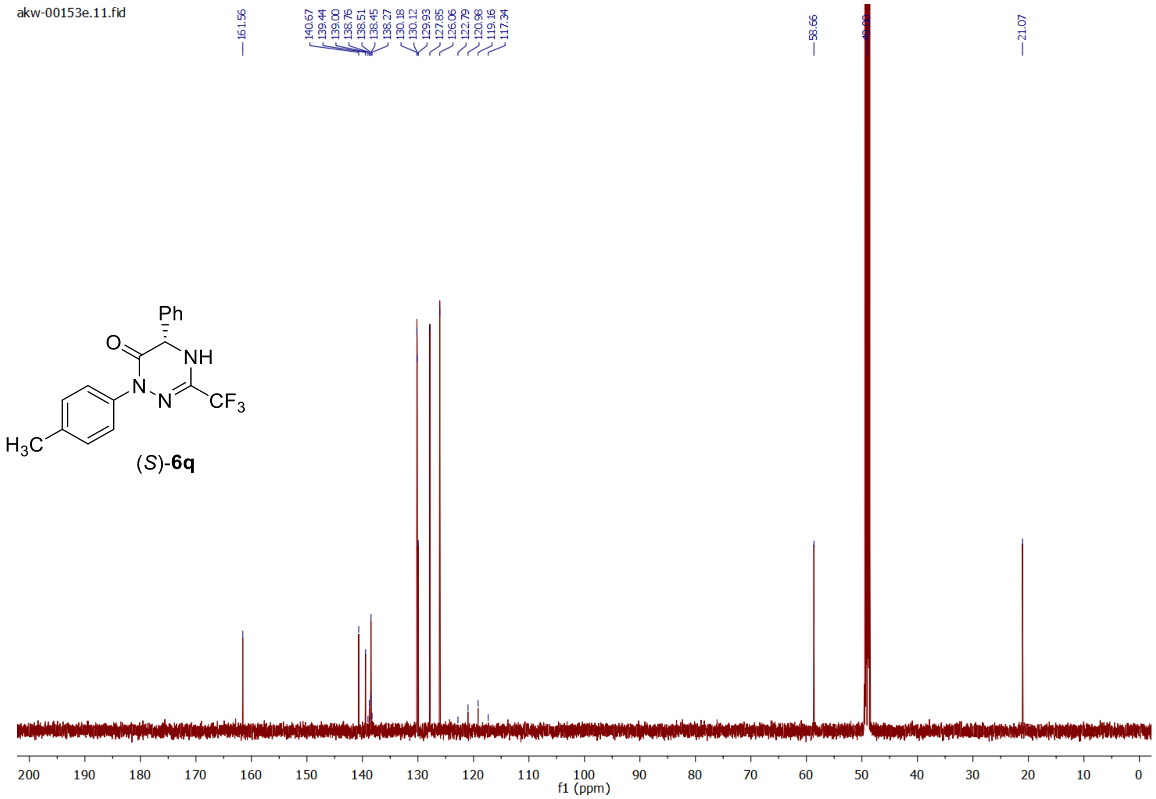

Figure S18. ^{1}H NMR (600 MHz, CD_3OD) and ^{13}C NMR (151 MHz, CD_3OD) spectra for compound (*S*)-**6q**.

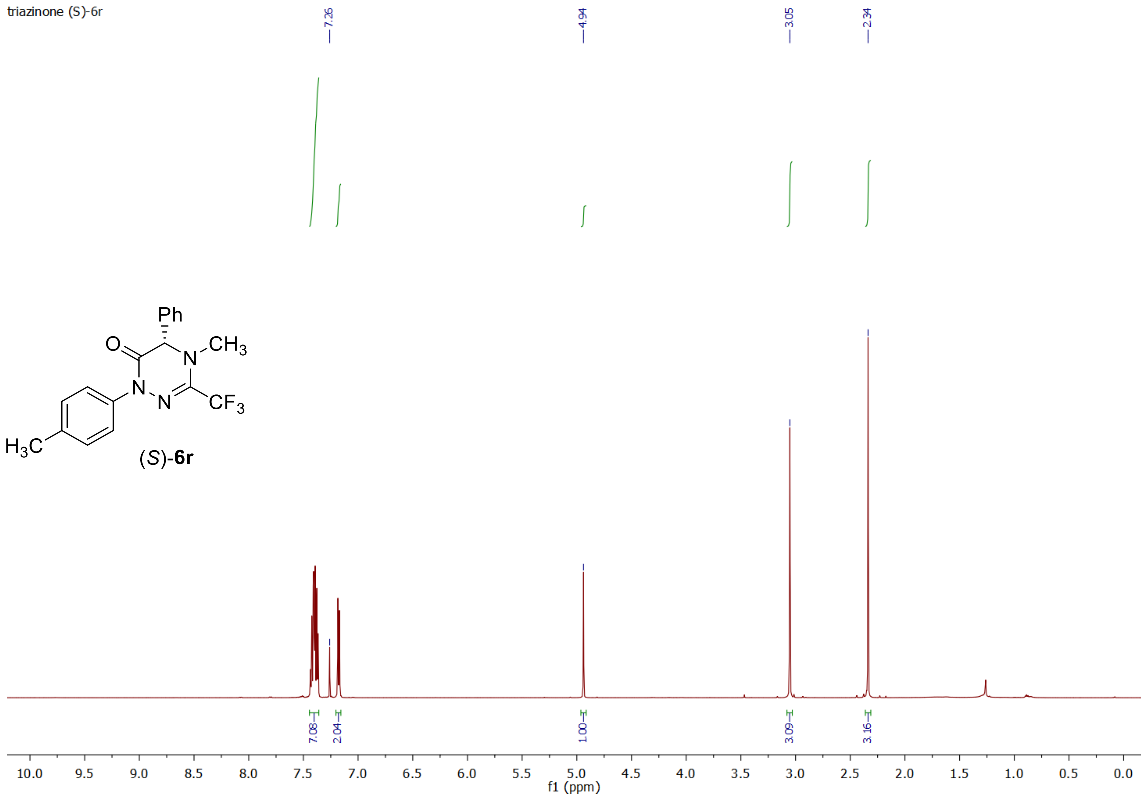

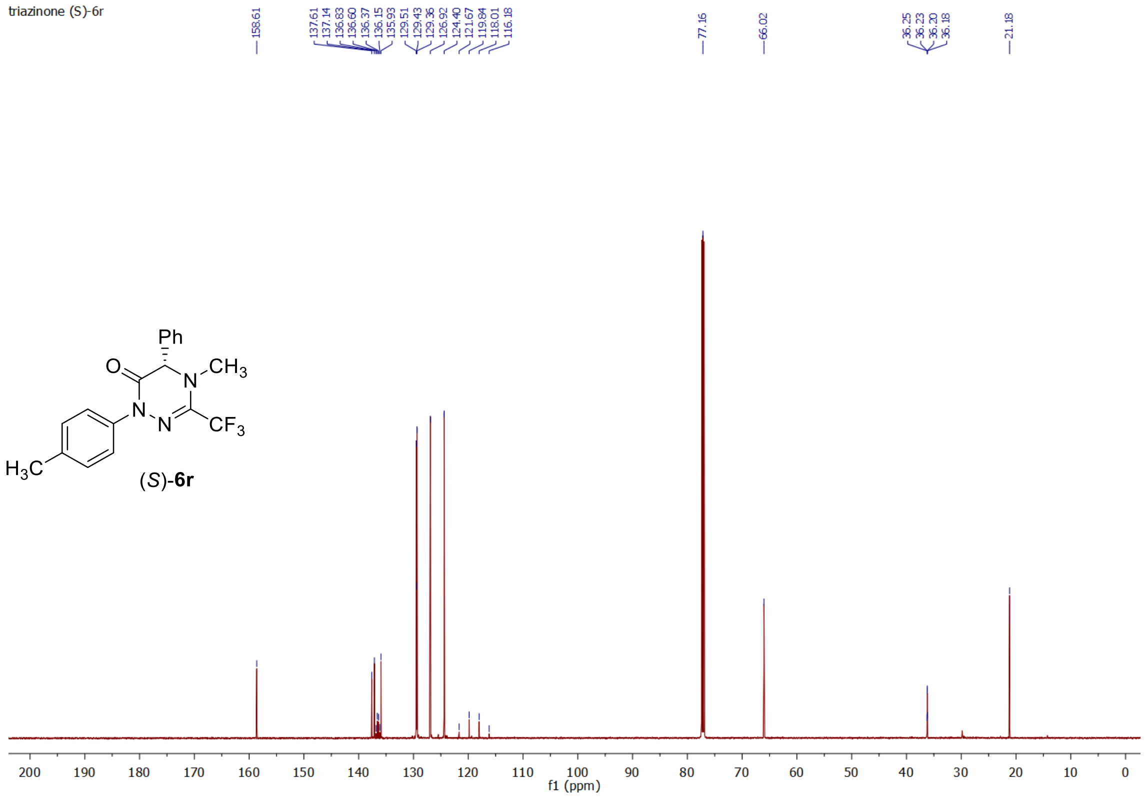

Figure S19. ^{1}H NMR (600 MHz, $CDCl_3$) and ^{13}C NMR (151 MHz, $CDCl_3$) spectra for compound (*S*)-**6r**.

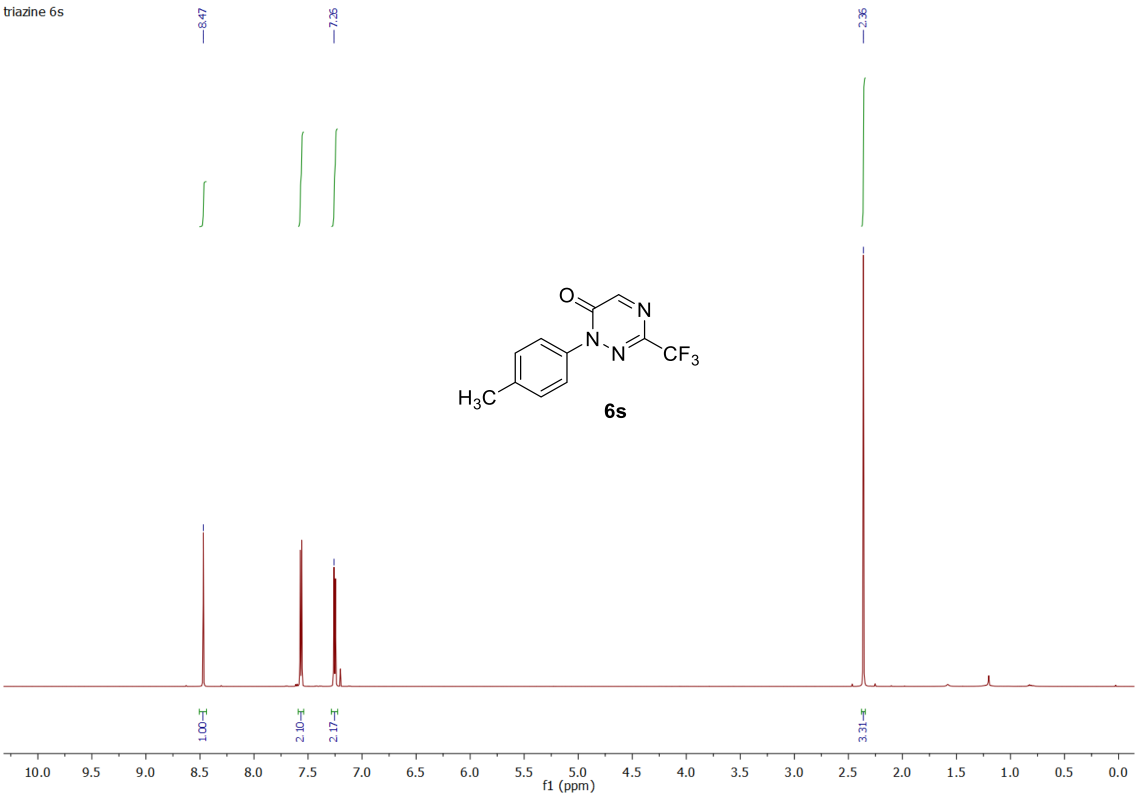

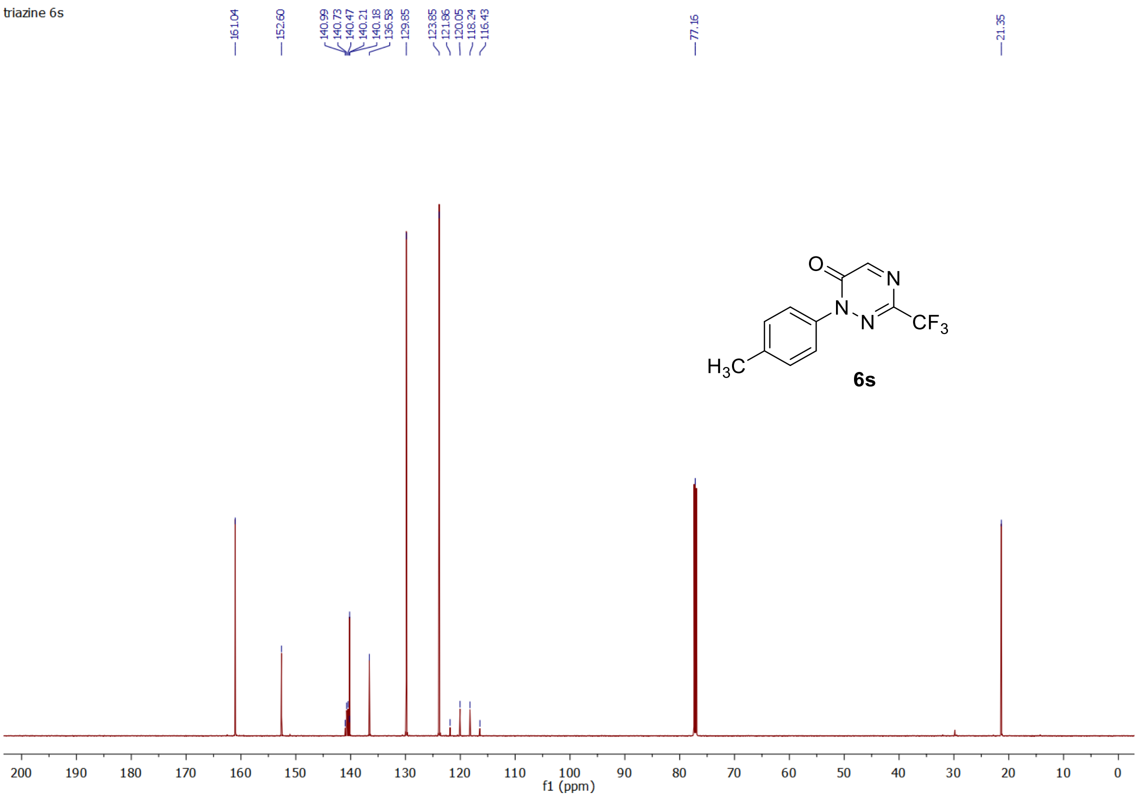

Figure S20. ^{1}H NMR (600 MHz, $CDCl_3$) and ^{13}C NMR (151 MHz, $CDCl_3$) spectra for compound **6s**.

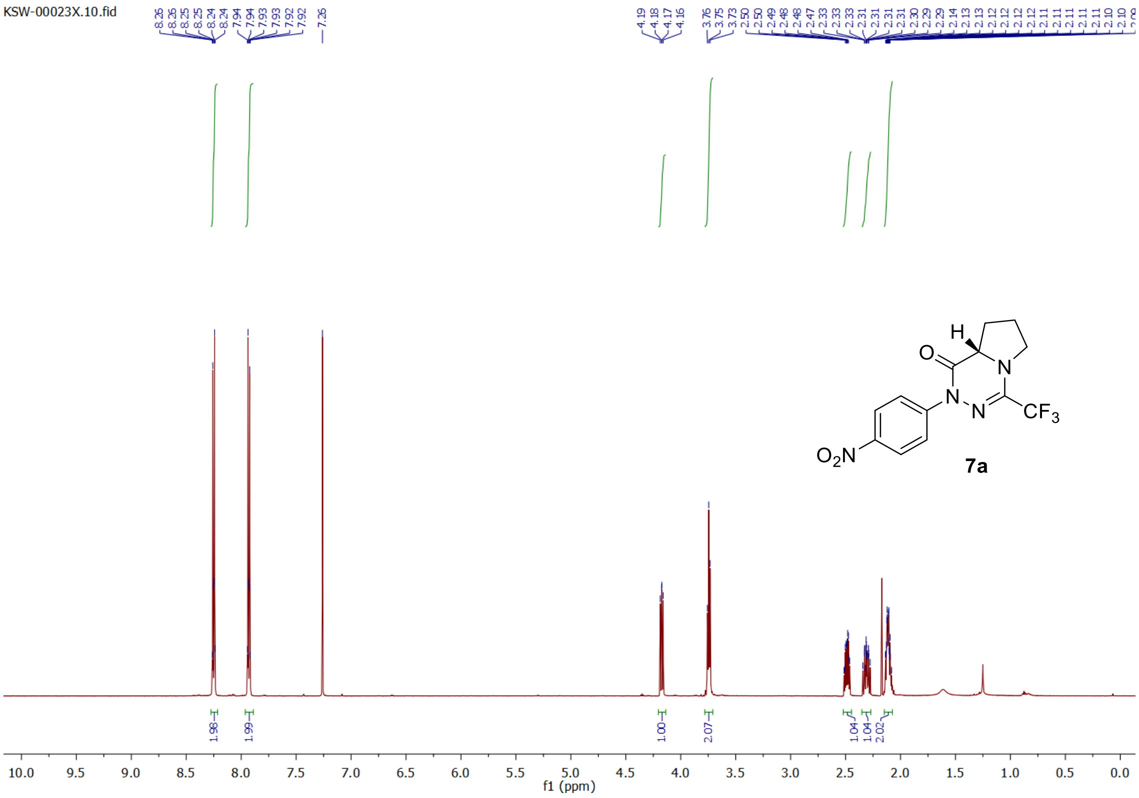

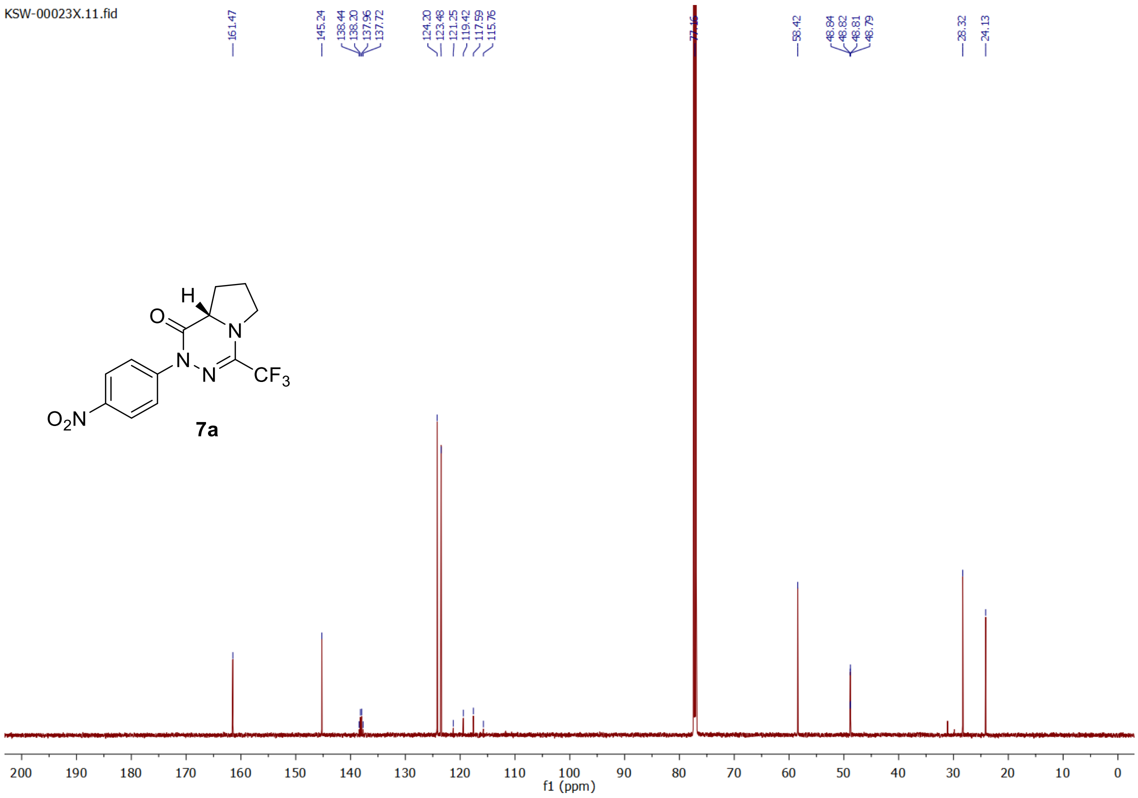

Figure S21. ^{1}H NMR (600 MHz, $CDCl_3$) and ^{13}C NMR (151 MHz, $CDCl_3$) spectra for compound **7a**.

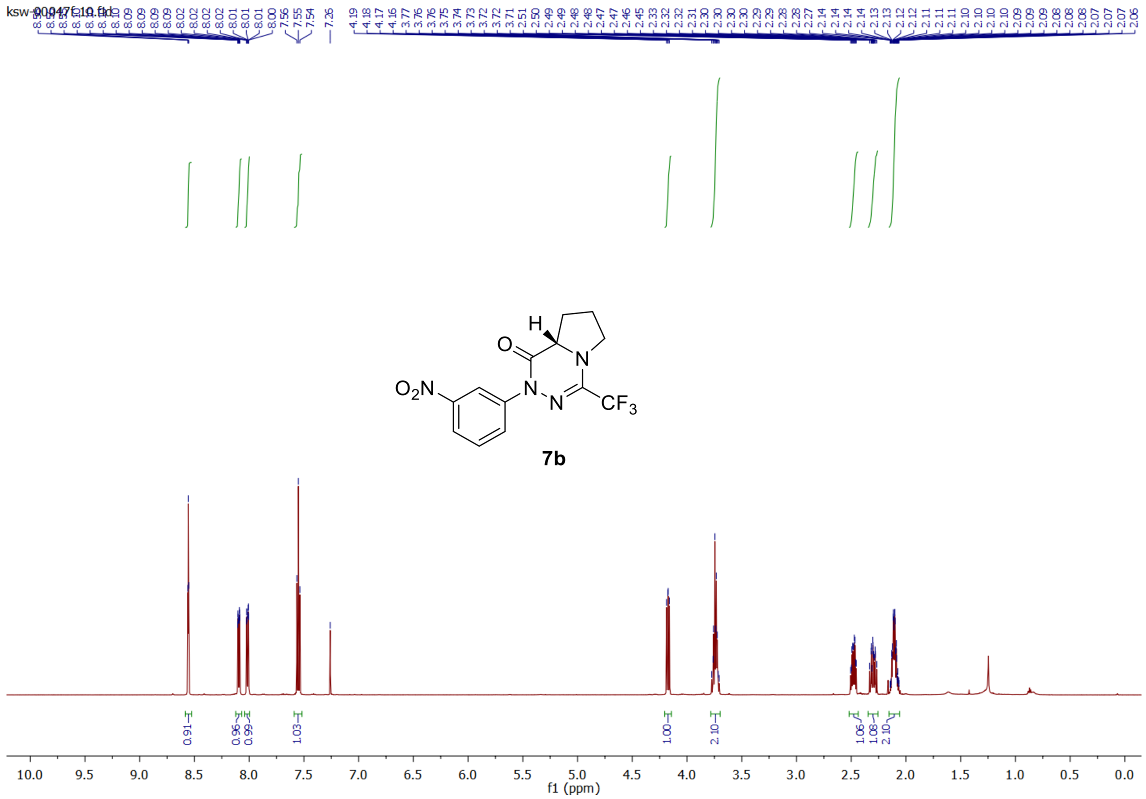

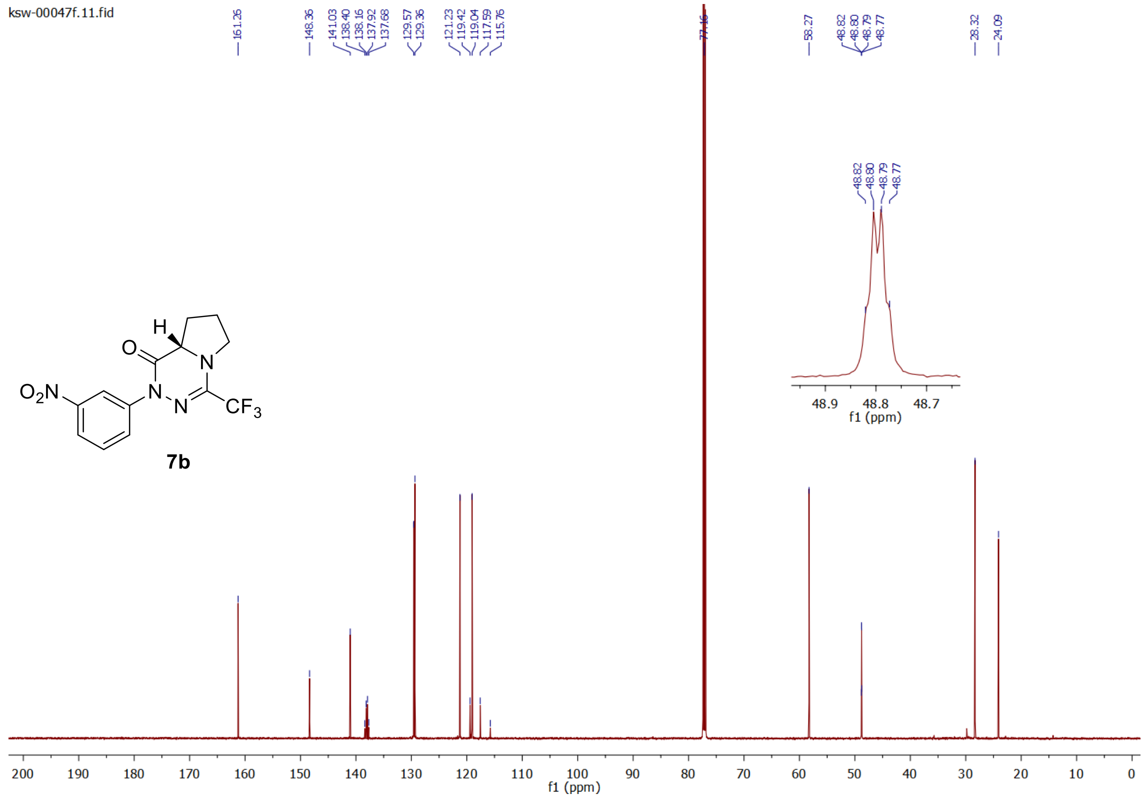

Figure S22. ^{1}H NMR (600 MHz, $CDCl_3$) and ^{13}C NMR (151 MHz, $CDCl_3$) spectra for compound **7b**.

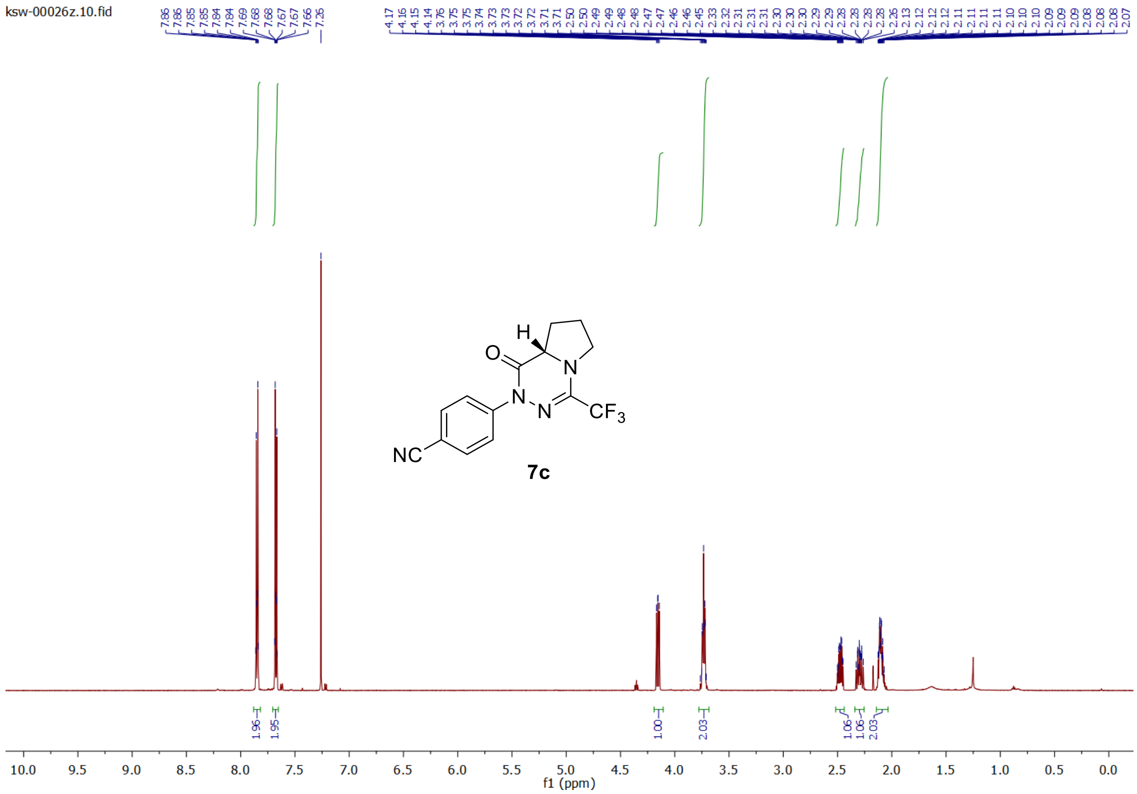

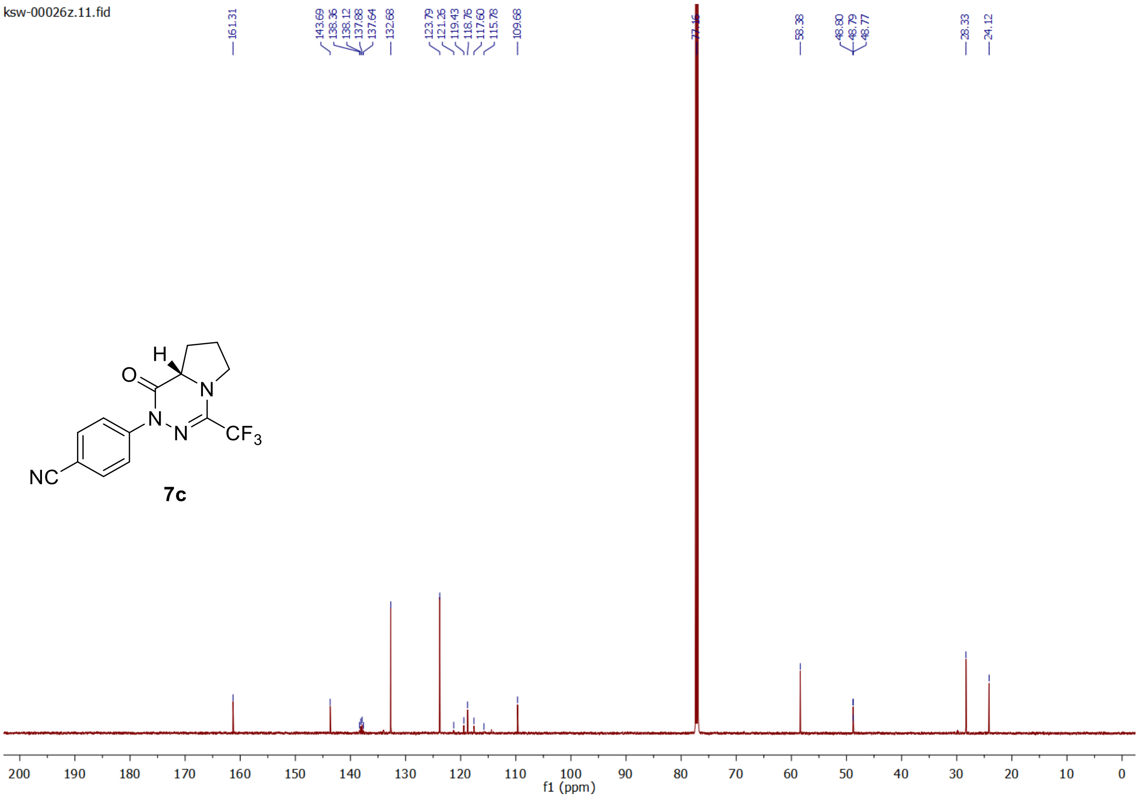

Figure S23. ^{1}H NMR (600 MHz, $CDCl_3$) and ^{13}C NMR (151 MHz, $CDCl_3$) spectra for compound **7c**.

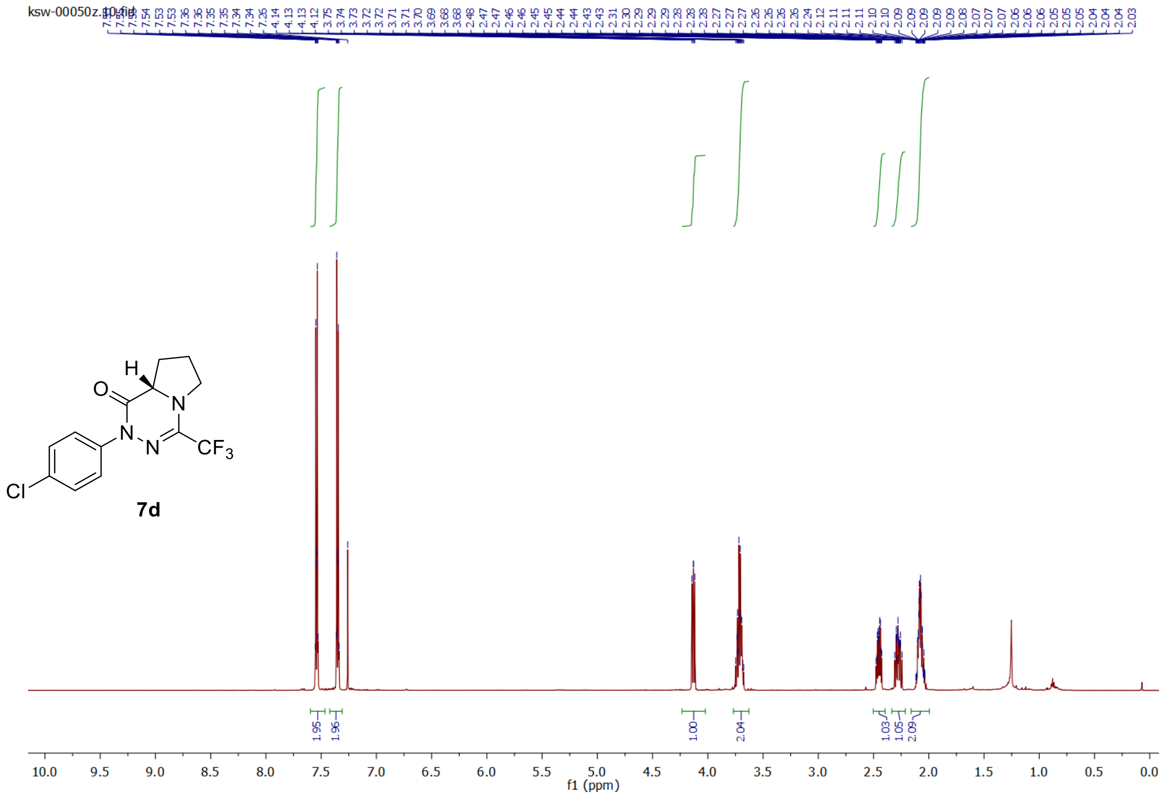

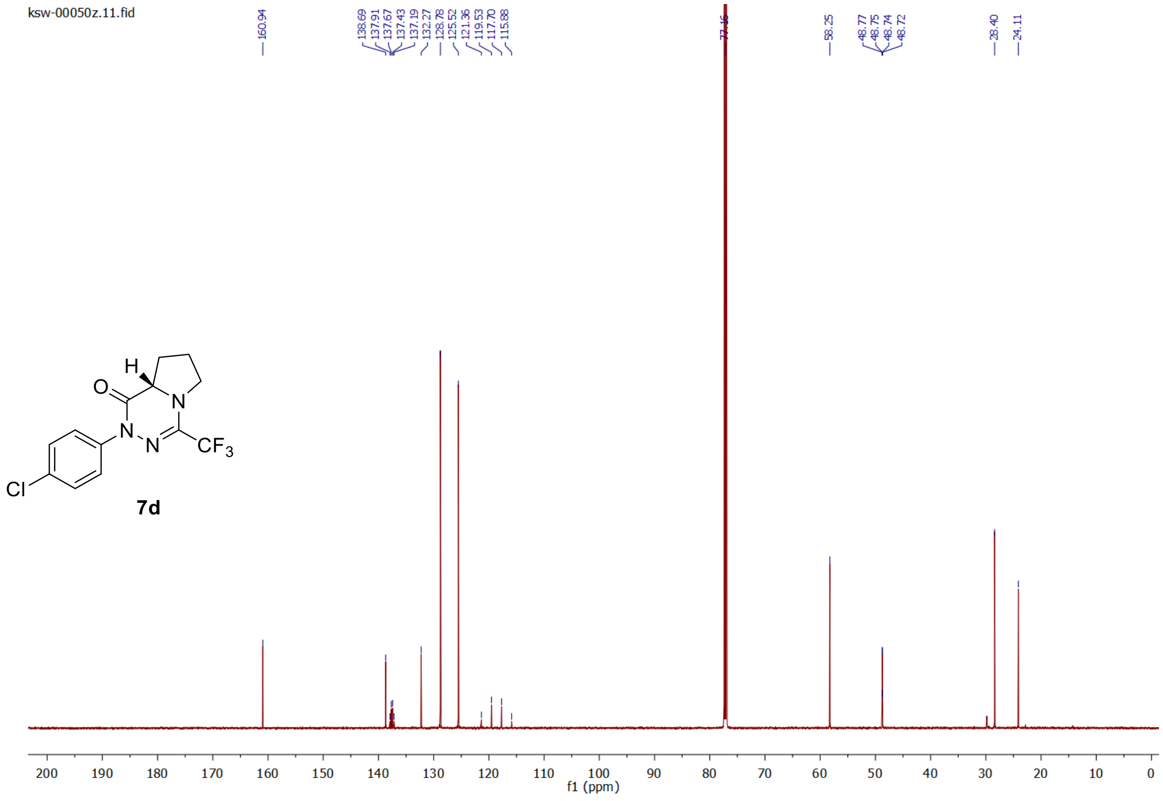

Figure S24. ^{1}H NMR (600 MHz, $CDCl_3$) and ^{13}C NMR (151 MHz, $CDCl_3$) spectra for compound **7d**.

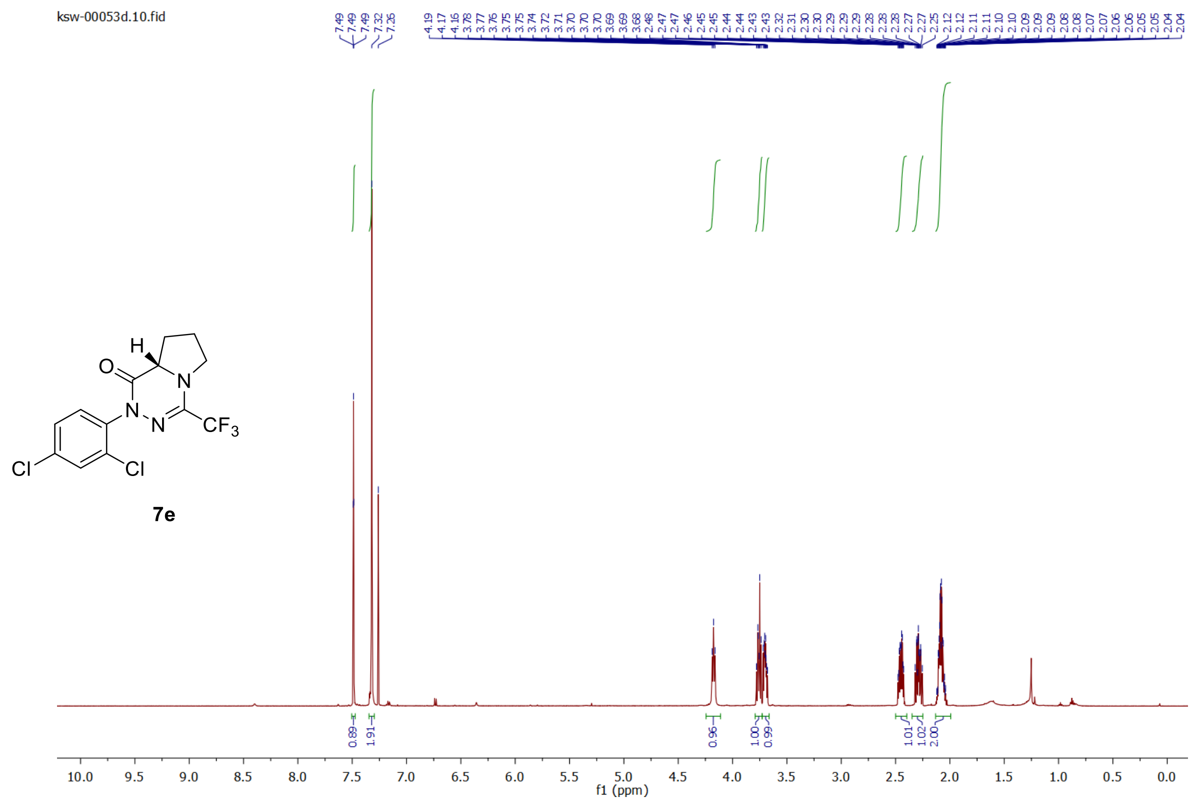

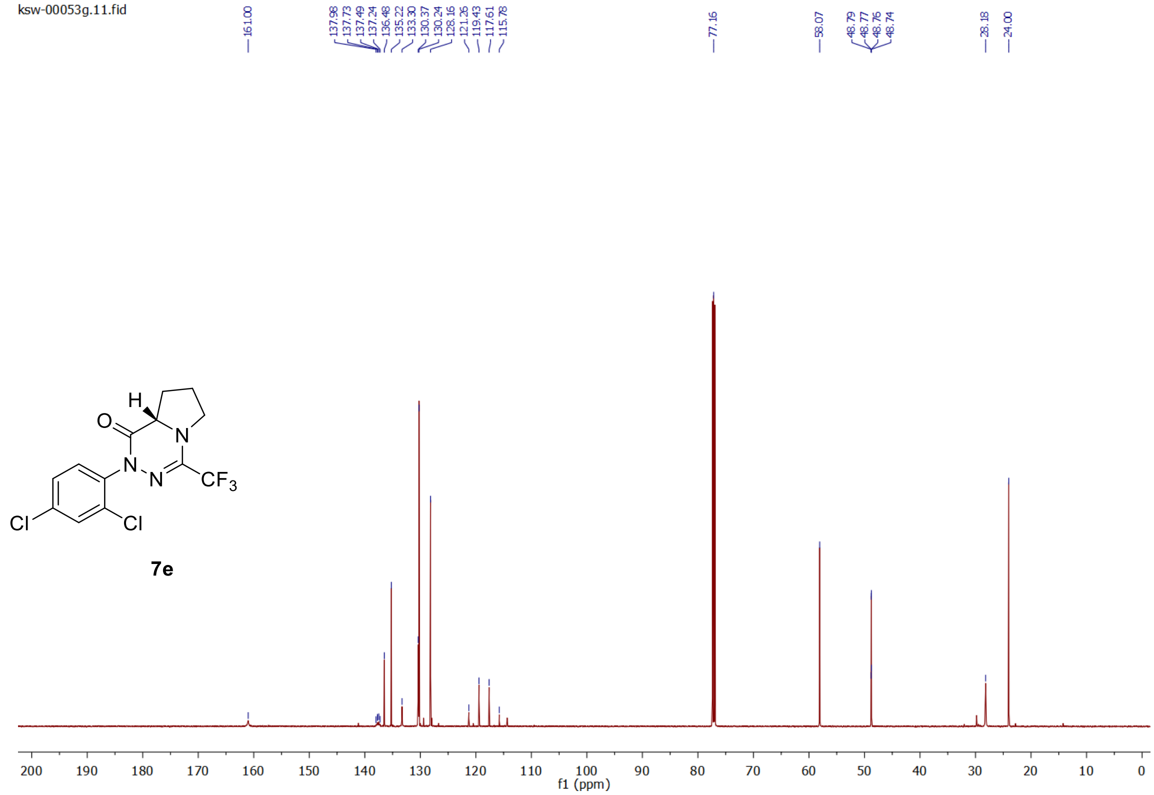

Figure S25. ^{1}H NMR (600 MHz, $CDCl_3$) and ^{13}C NMR (151 MHz, $CDCl_3$) spectra for compound **7e**.

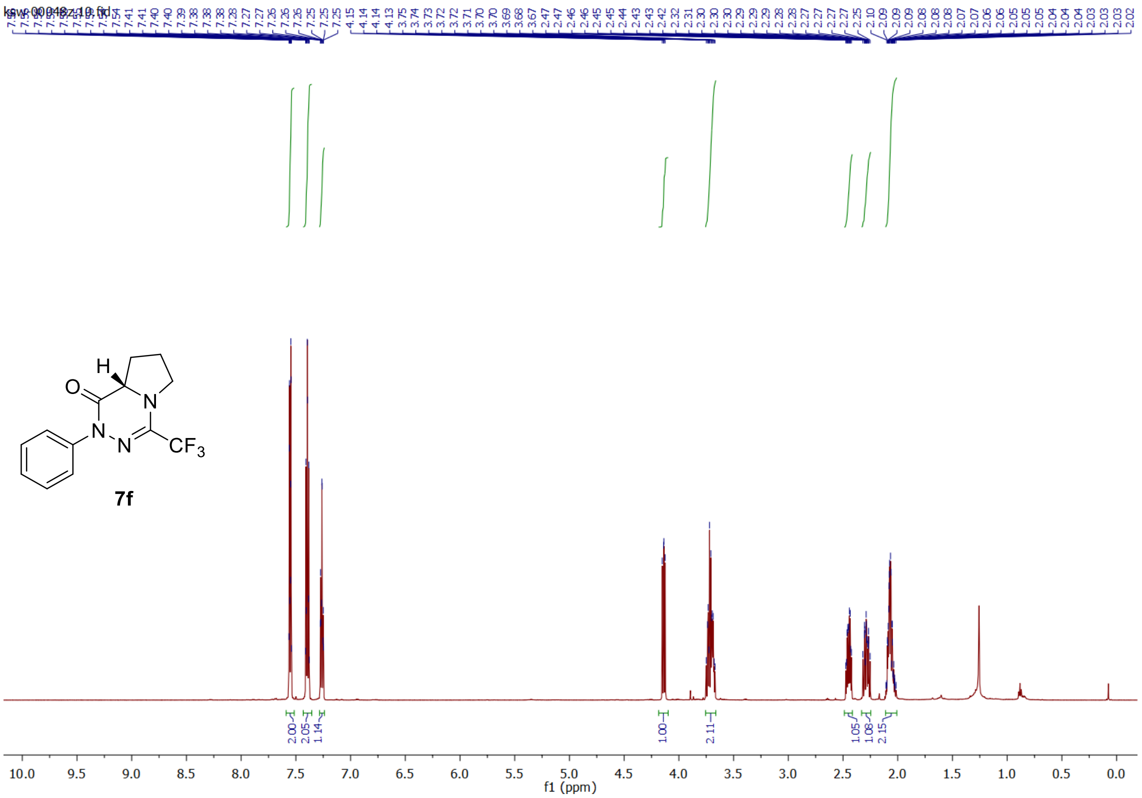

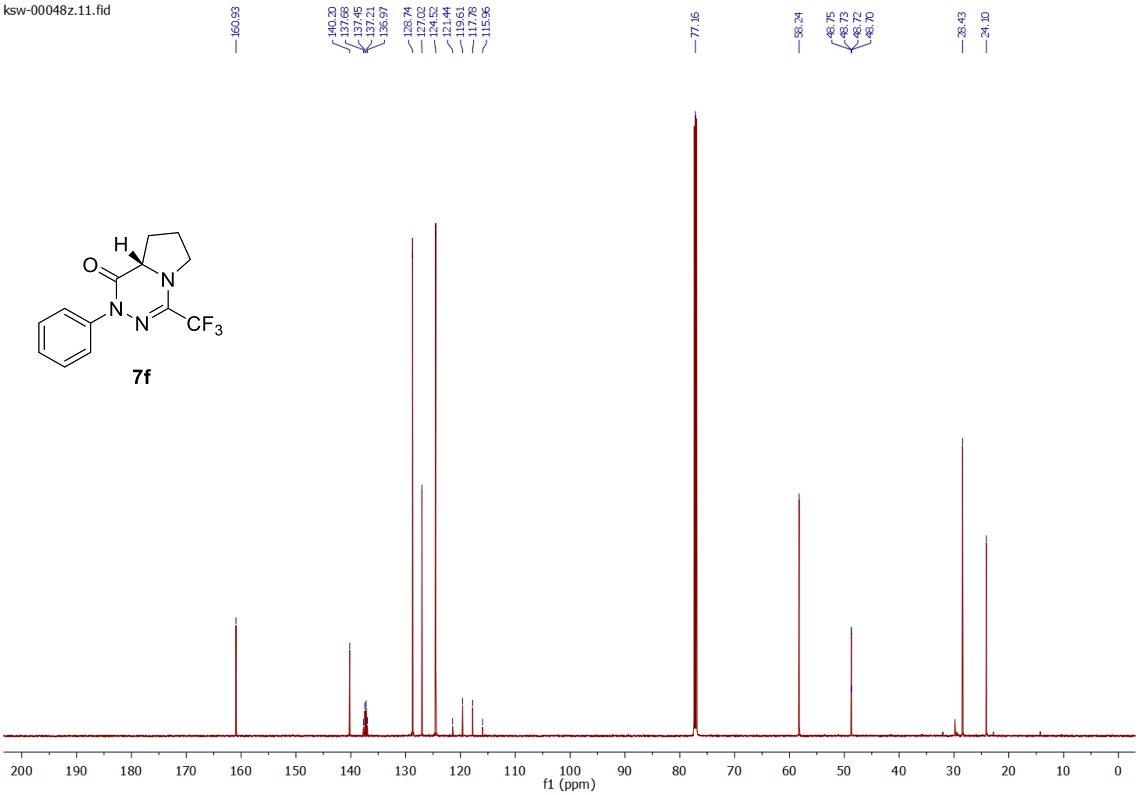

Figure S26. ^{1}H NMR (600 MHz, CDCl₃) and ^{13}C NMR (151 MHz, CDCl₃) spectra for compound **7f**.

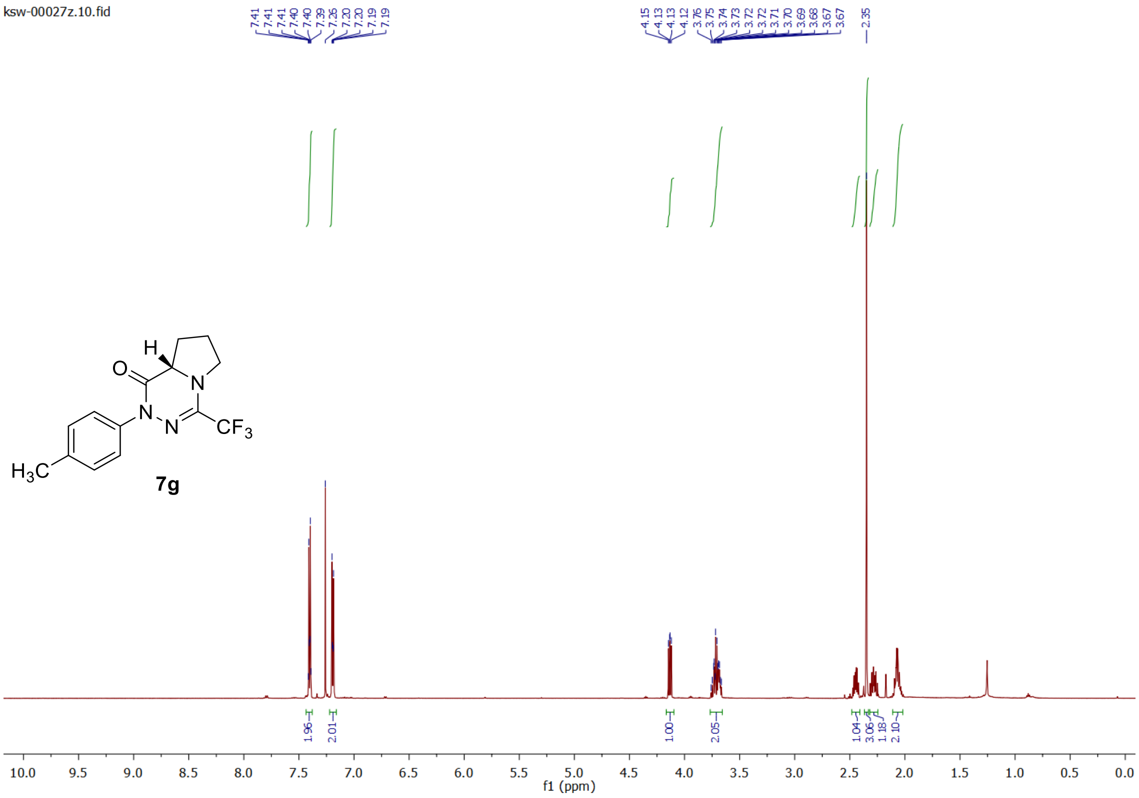

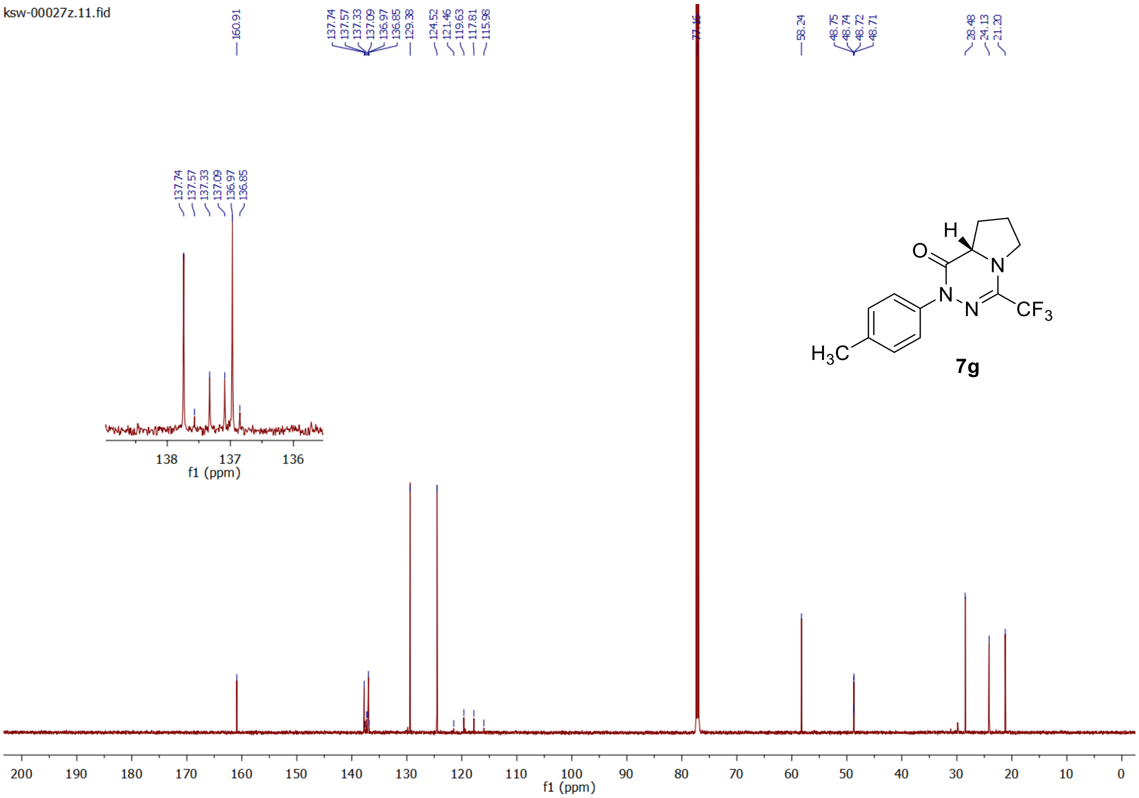

Figure S27. ^{1}H NMR (600 MHz, $CDCl_3$) and ^{13}C NMR (151 MHz, $CDCl_3$) spectra for compound **7g**.

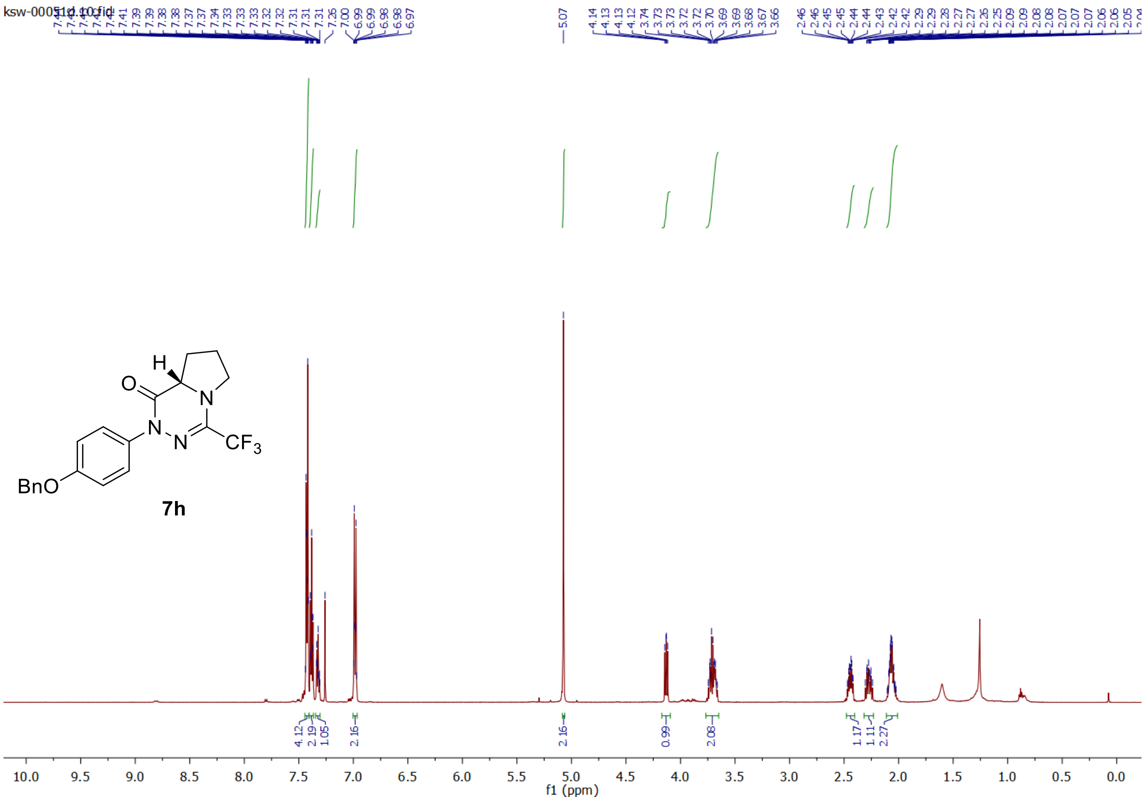

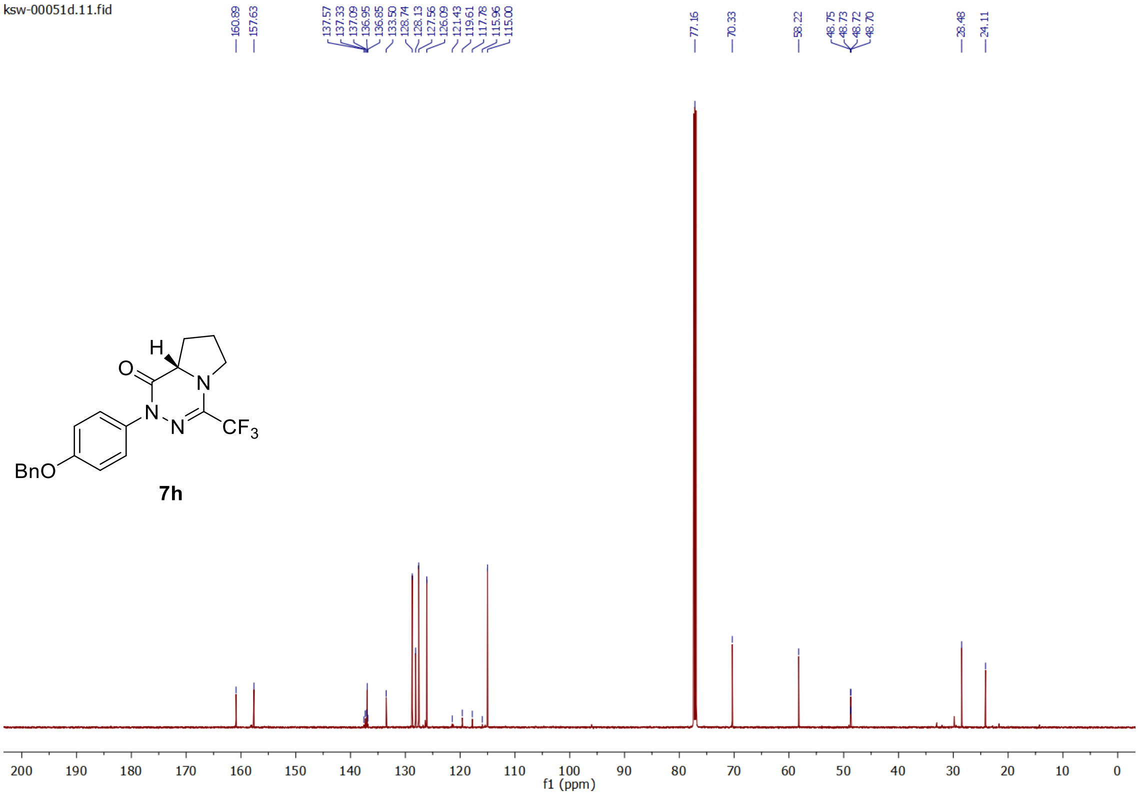

Figure S28. ^{1}H NMR (600 MHz, $CDCl_3$) and ^{13}C NMR (151 MHz, $CDCl_3$) spectra for compound **7h**.

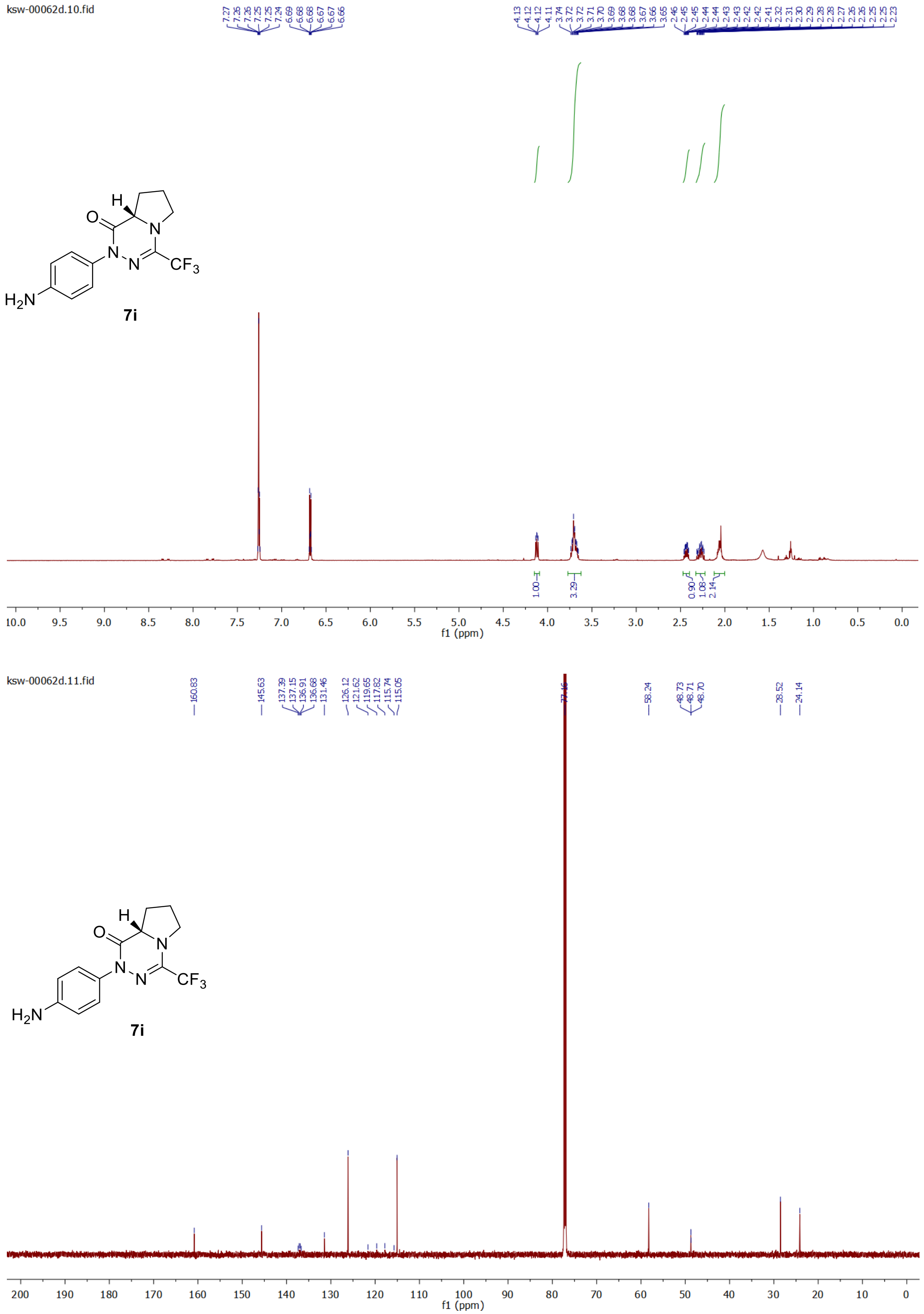

Figure S29. ^{1}H NMR (600 MHz, $CDCl_3$) and ^{13}C NMR (151 MHz, $CDCl_3$) spectra for compound **7i**.

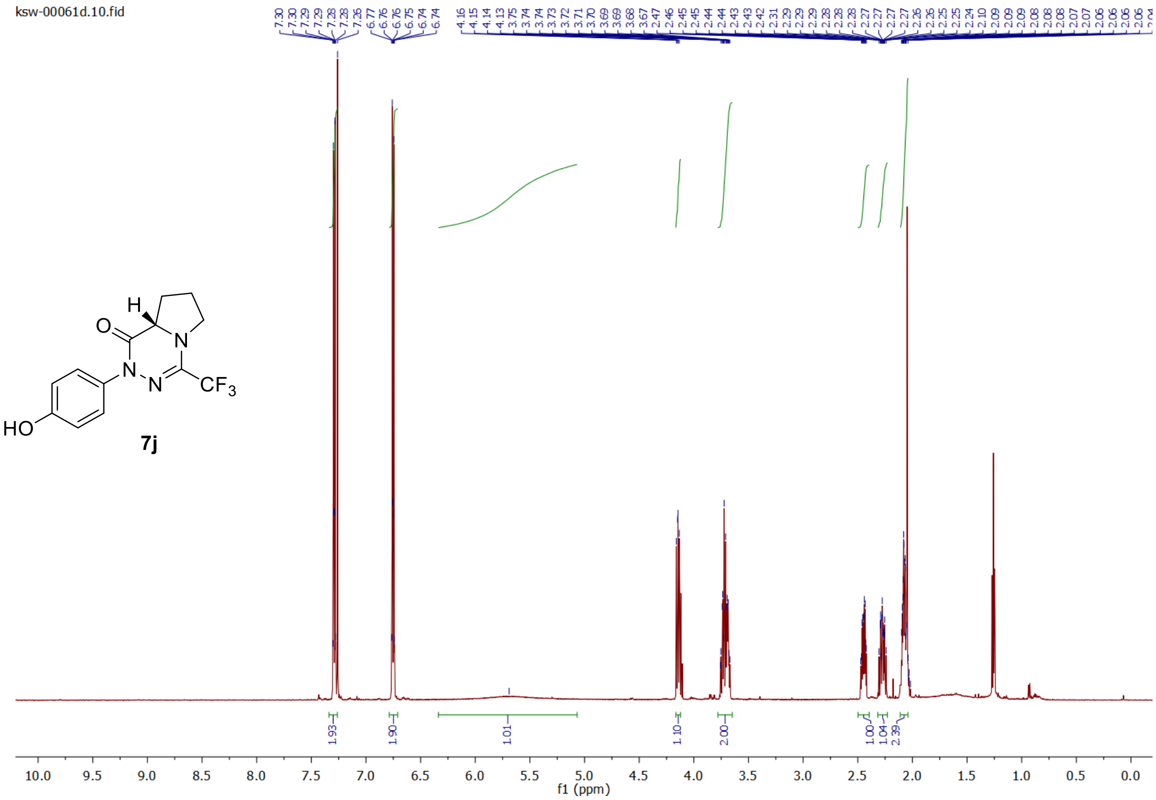

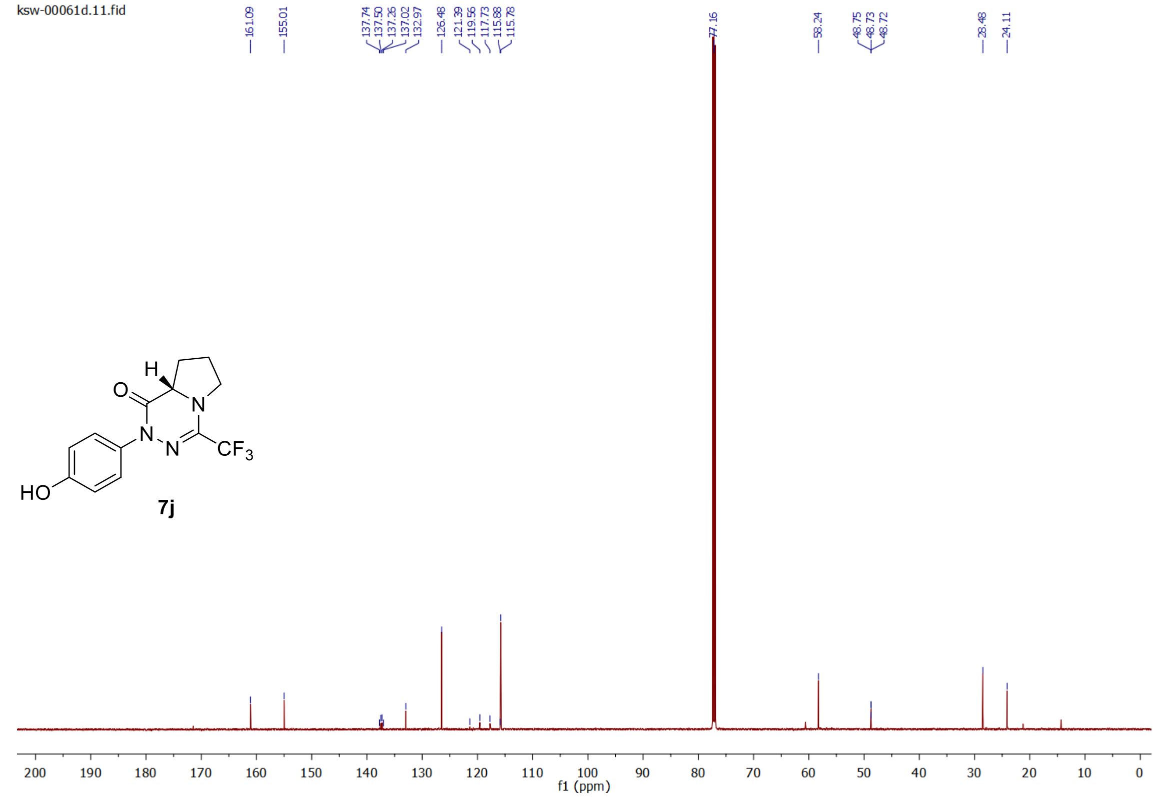

Figure S30. ^{1}H NMR (600 MHz, $CDCl_3$) and ^{13}C NMR (151 MHz, $CDCl_3$) spectra for compound **7j**.

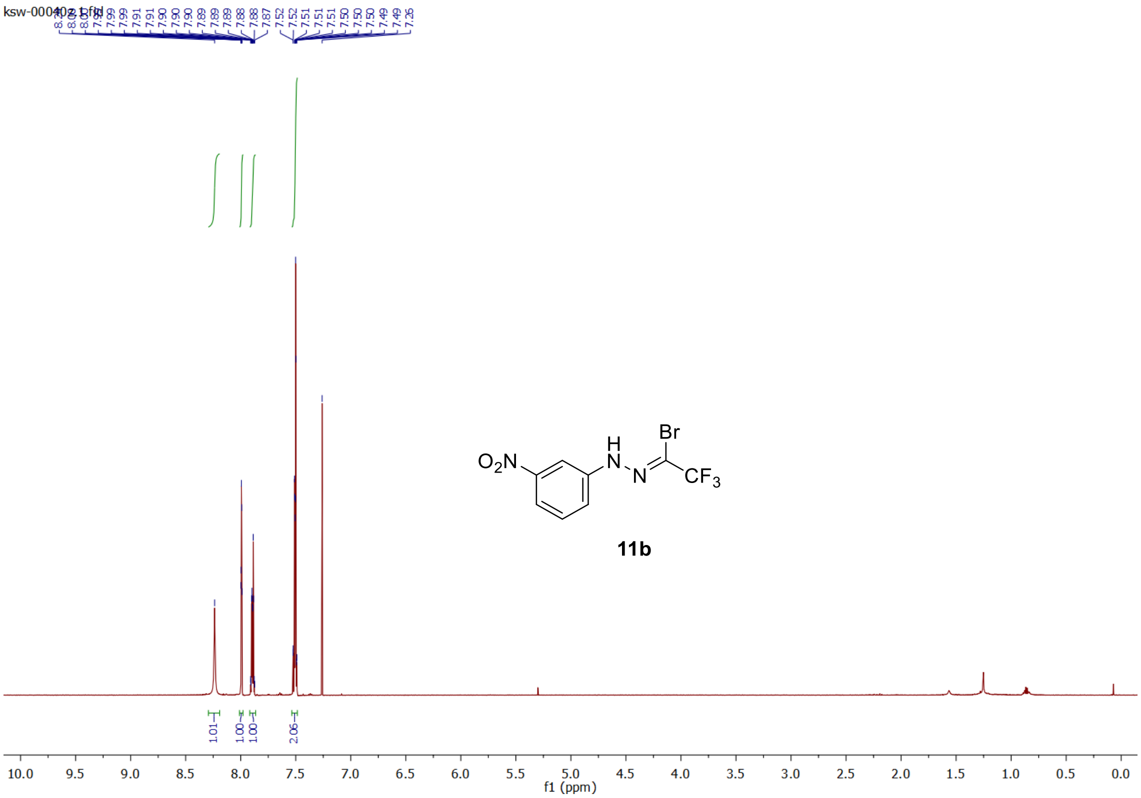

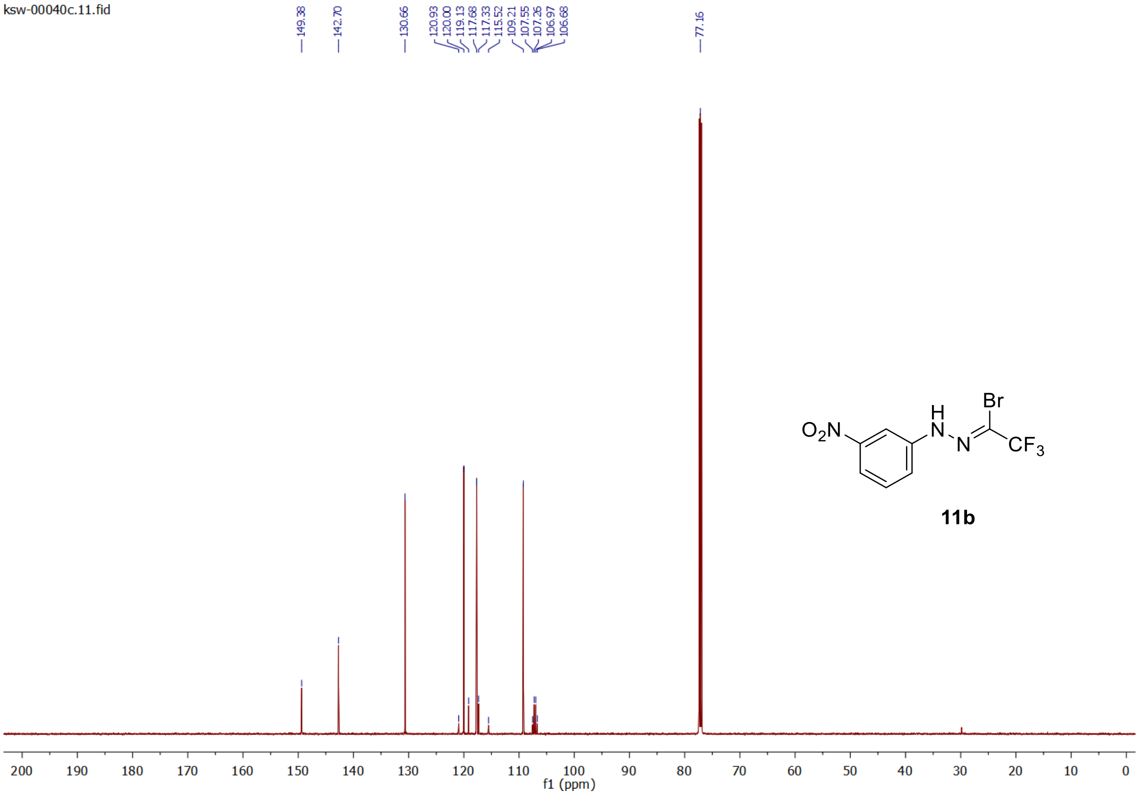

Figure S31. ^{1}H NMR (600 MHz, $CDCl_3$) and ^{13}C NMR (151 MHz, $CDCl_3$) spectra for compound **11b**.

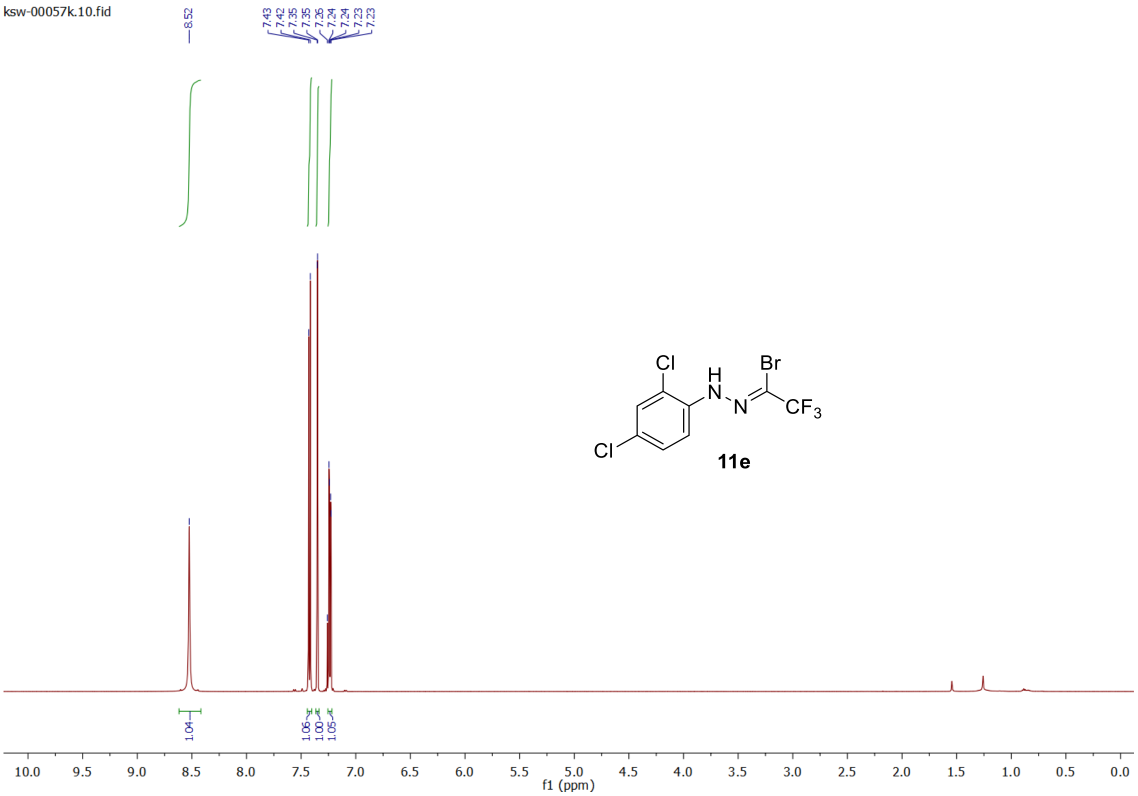

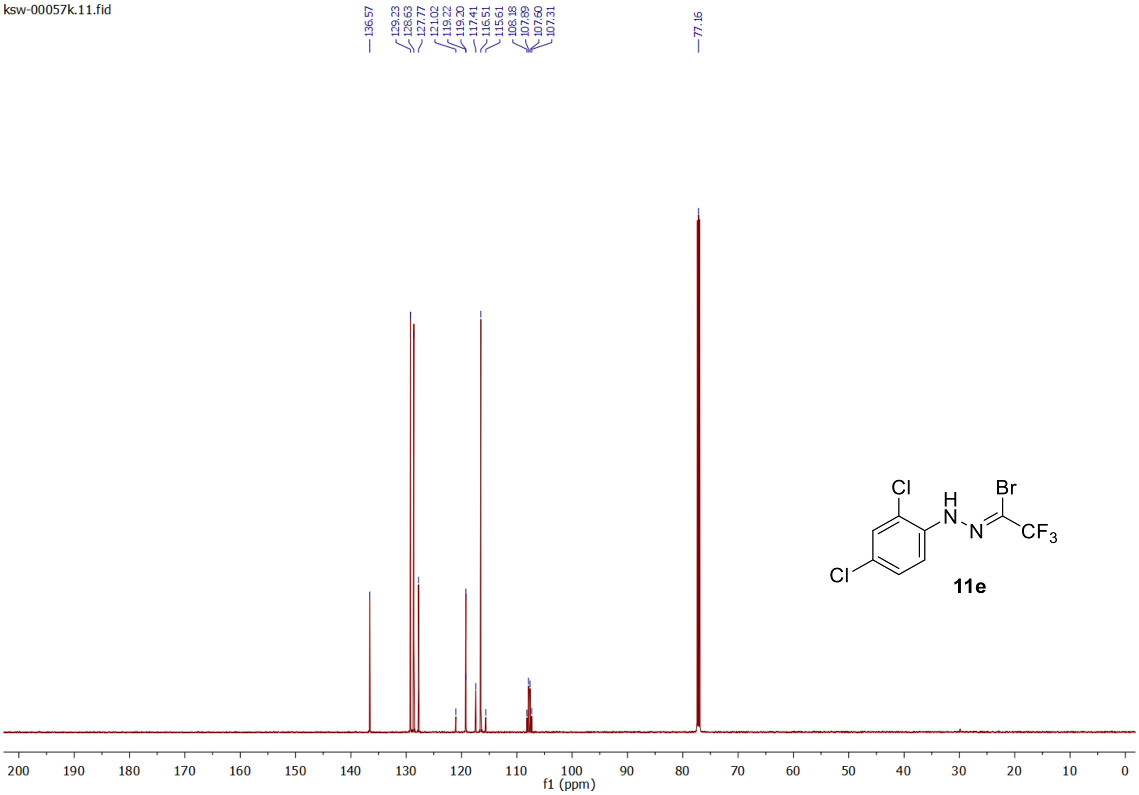

Figure S32. ^{1}H NMR (600 MHz, $CDCl_3$) and ^{13}C NMR (151 MHz, $CDCl_3$) spectra for compound **11e**.

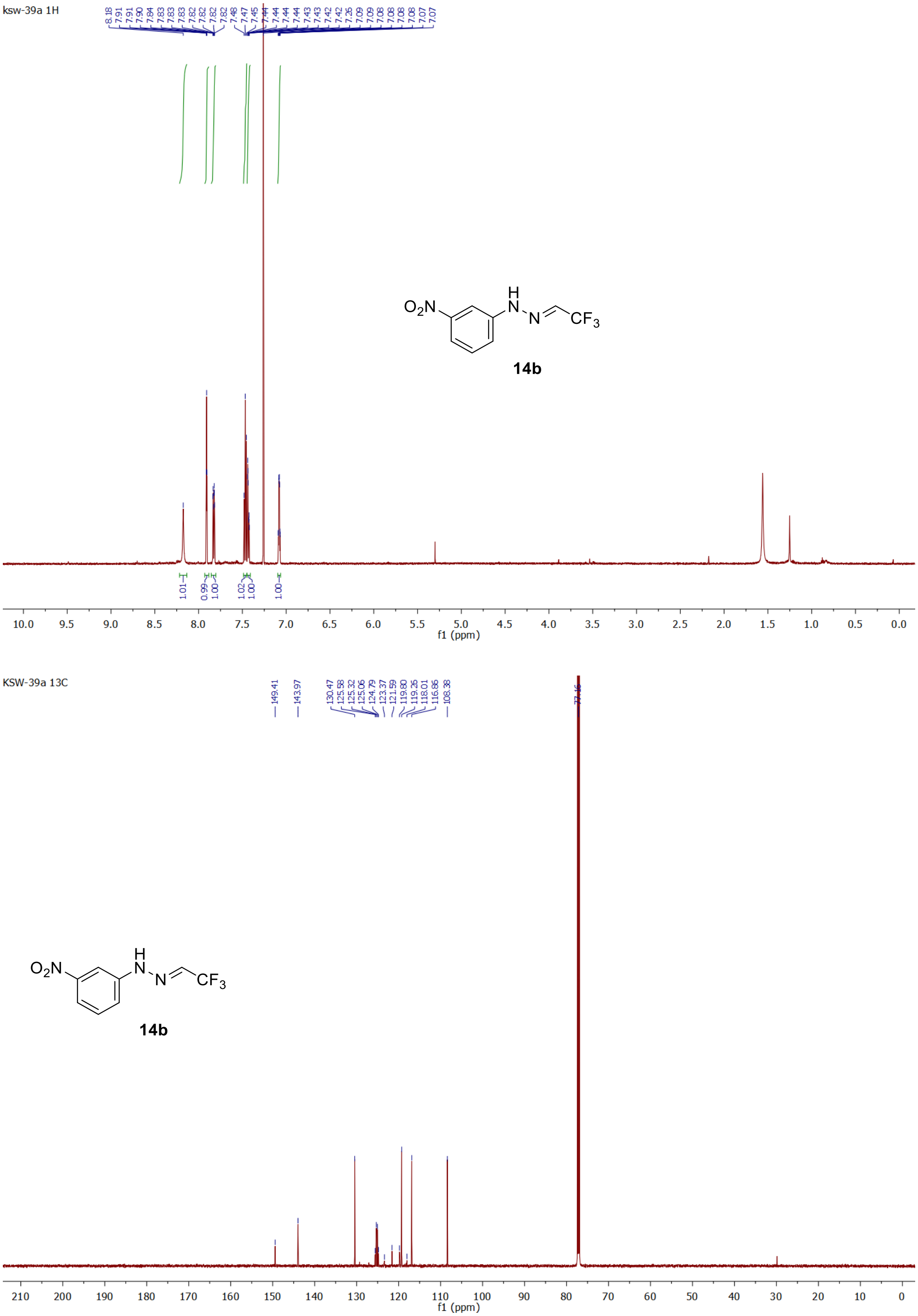

Figure S33. ^{1}H NMR (600 MHz, $CDCl_3$) and ^{13}C NMR (151 MHz, $CDCl_3$) spectra for compound **14b**.

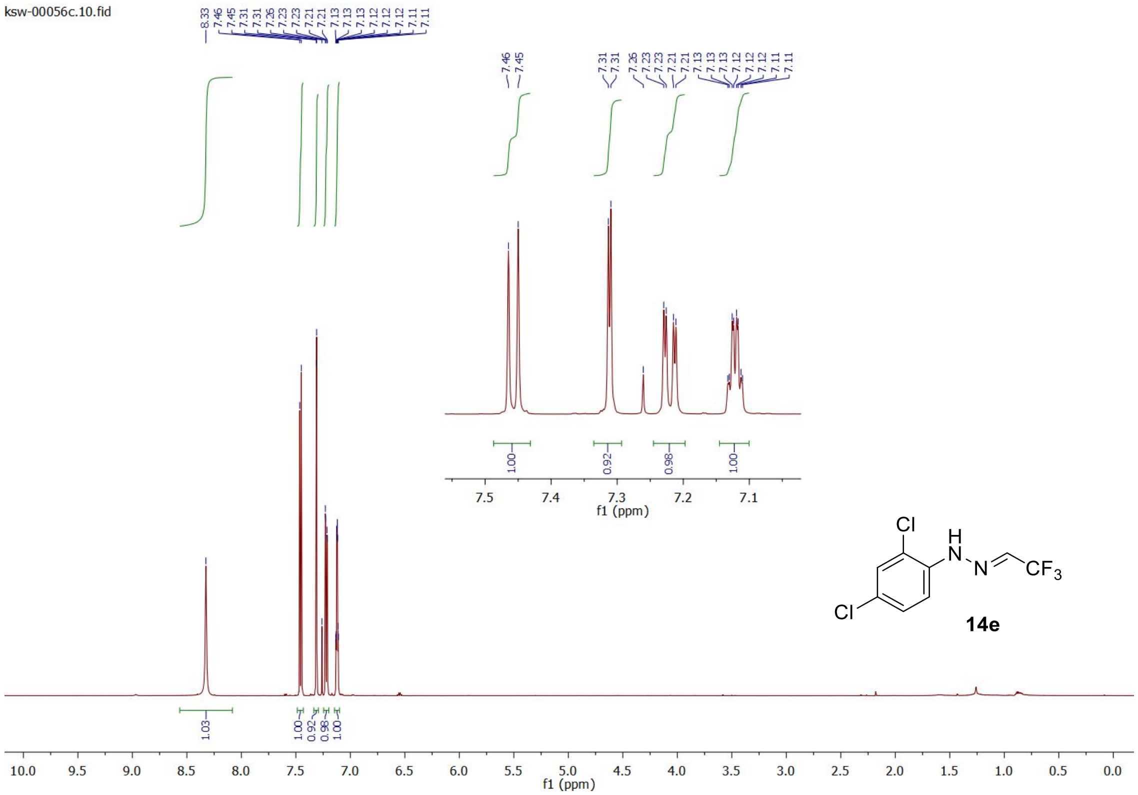

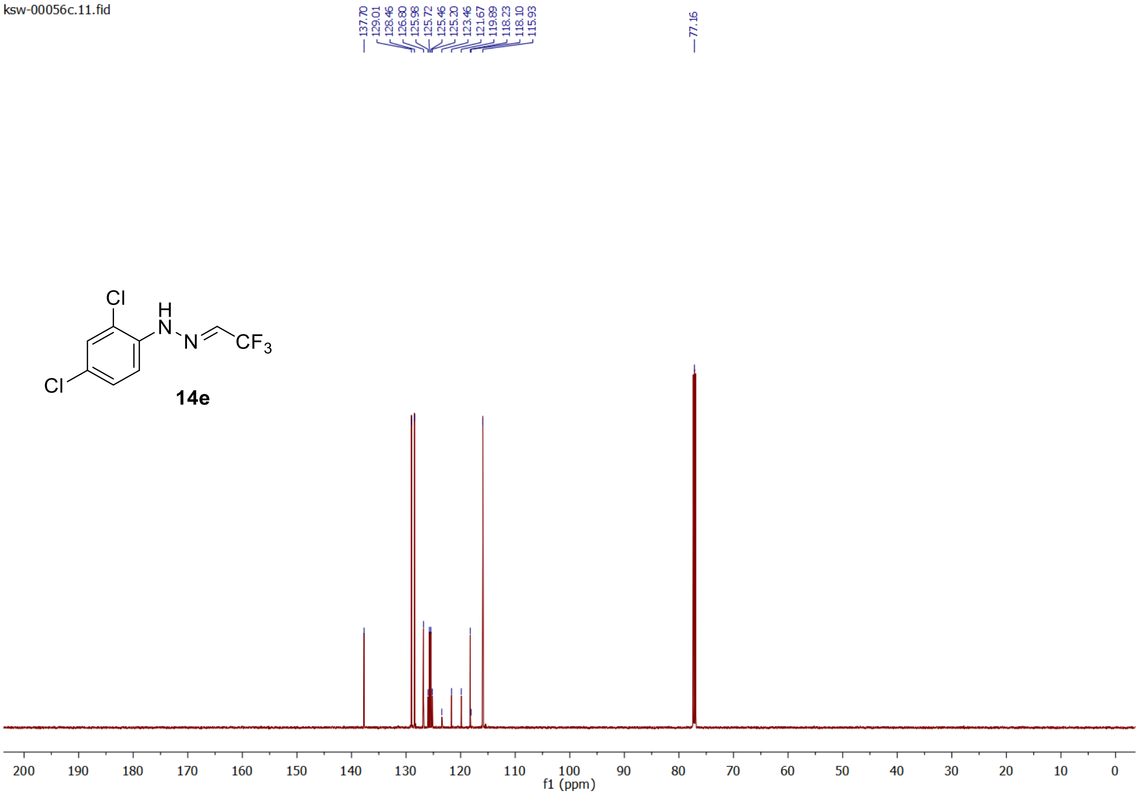

Figure S34. ^{1}H NMR (600 MHz, $CDCl_3$) and ^{13}C NMR (151 MHz, $CDCl_3$) spectra for compound **14e**.

HPLC analyses

HPLC chromatograms of racemic and *S*-configured 4-methyl-5-phenyl-1-(4-tolyl)-3-trifluoromethyl-4,5-dihydro-1*H*-[1,2,4]triazin-6-one (**6r**): CHIRALPAK® AD-H column {amylose *tris*(3,5-dimethylphenyl-carbamate) coated on 5 µm silica-gel}; hexane:^{i}PrOH = 90:10, flow = 0.5 mL/min, 253 nm.

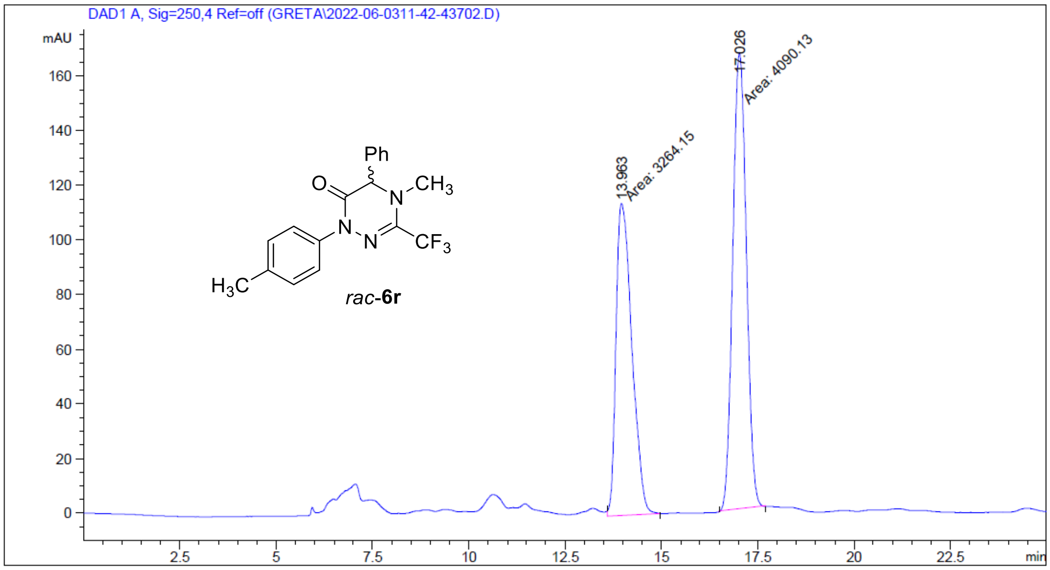

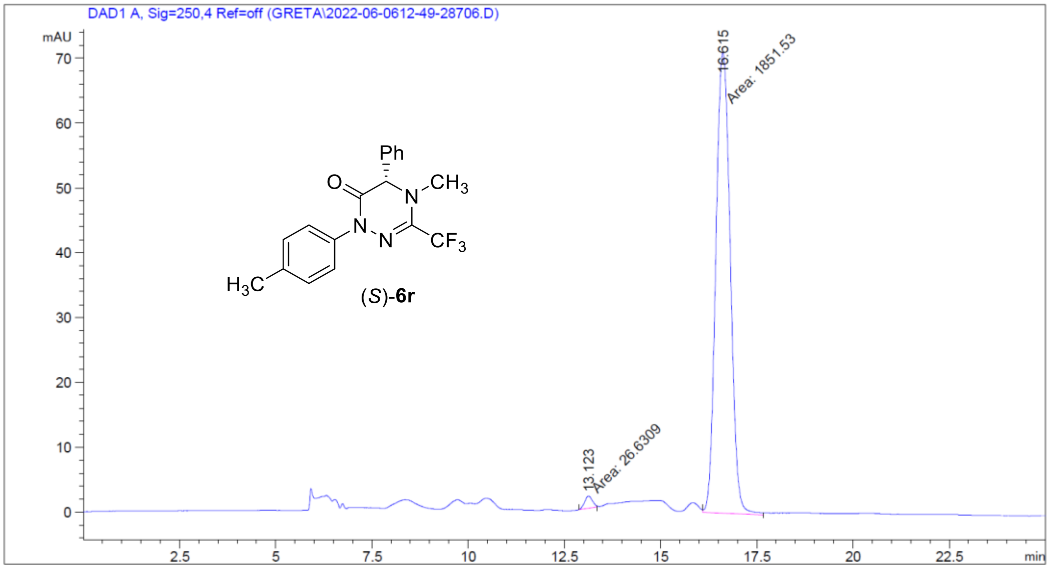

Cytotoxicity tests

Cell culture and treatment: The promyelocytic leukemia (HL-60) and breast cancer adenocarcinoma (MCF-7) cell lines were purchased from the European Collection of Cell Cultures (ECACC). Leukemia cells were cultured in RPMI 1640 plus GlutaMax I medium (Gibco/Life Technologies, Carlsbad, CA, USA). MCF-7 cells were maintained in Minimum Essential Medium Eagle (Sigma Aldrich, St. Louis, MO, USA) supplemented with 2 mM glutamine and Men Non-essential amino acid solution (Sigma Aldrich, St. Louis, MO, USA). Both media were supplemented with 10% heat-inactivated fetal bovine serum (Biological Industries, Beit-Haemek, Israel) and antibiotics (100 U/mL penicillin and 100 µg/mL streptomycin) (Sigma-Aldrich, St. Louis, MO, USA). Cells were maintained at 37°C in 5% CO_2 atmosphere and grown until 80% confluent.
The tested compounds were dissolved in DMSO and further diluted with culture medium. The final concentration of DMSO in cell cultures was less than 0.1% v/v.

In vitro cytotoxicity assay: The MTT [3-(4, 5-dimethylthiazol-2-yl)-2, 5-diphenyltetrazolium bromide] assay was performed according to the known procedure [1]. Cells were seeded into 24-well plates at a density of 8 × 10^4/mL and left to grow for 24 h. After being cultured for 48 h with various concentrations of the tested compounds, cells were incubated with MTT solution (100 µL, 5 mg/mL in phosphate buffered saline) for 2 h. Then, the plates were centrifuged and the supernatant was discarded. DMSO (1 mL) was added to each well to dissolve the blue formazan product, whose absorbance was measured at 560 nm using FlexStation 3 Multi-Mode Microplate Reader (Molecular Devices, LLC, CA, USA). The untreated cells were used as control. The data were expressed as mean ± SEM of three independent experiments.

Table S1. *In vitro* cytotoxic activity of 1,2,4-triazinones **6** and **7** on selected cancer cell lines.

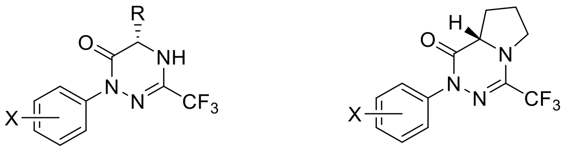

6a–6q **7a–7h**

	X	R	IC_{50} [µM] [1]	
			HL-60	MCF-7
**6a**	4-NO_2	H	>100	>100
**6b**	3-NO_2	H	>100	>100
**6c**	4-CN	H	>100	>100
**6d**	4-Cl	H	>100	>100
**6e**	2,4-Cl_2	H	>100	>100
**6f**	H	H	>100	>100
**6g**	4-CH_3	H	>100	>100
**6h**	4-BnO	H	>100	>100
**6i**	4-NO_2	CH_3	>100	>100
**6j**	4-NO_2	$CH(CH_3)_2$	93.04±4.52	>100
**6k**	4-NO_2	$CH_2CH(CH_3)_2$	58.10±3.78	97.50±3.54

**6l**	4-NO_2	Ph	75.94±5.50	>100
**6m**	4-NO_2	CH_2CH_2OH	23.04±1.85	>100
**6n**	4-NO_2	$CH_2CH_2SCH_3$	>100	>100
**6o**	4-NO_2	CH_2COOCH_3	>100	>100
**6p**	4-NO_2	1*H*-indol-3-yl	86.33±2.16	99.25±2.05
**6q**	4-CH_3	Ph	>100	>100
**7a**	4-NO_2	–	>100	>100
**7b**	3-NO_2	–	90.58±4.32	>100
**7c**	4-CN	–	45.44±3.67	>100
**7d**	4-Cl	–	>100	>100
**7e**	2,4-Cl_2	–	>100	>100
**7f**	H	–	>100	>100
**7g**	4-CH_3	–	>100	>100
**7h**	4-BnO	–	56.23±1.23	>100

[1] Compound concentration required to inhibit metabolic activity by 50%. Values are expressed as mean ± SEM from concentration-response curves of at least three experiments using a nonlinear estimation (quasi-Newton algorithm) method.

References

[1] Mosmann T. Rapid colorimetric assay for cellular growth and survival: application to proliferation and cytotoxicity assays. *J. Immunol. Methods* **1983**, *65*, 55–63. [doi: 10.1016/0022-1759(83)90303-4]
